# Biosynthesis of the Enterotoxic Pyrrolobenzodiazepine Natural Product Tilivalline

**DOI:** 10.1002/anie.201707737

**Published:** 2017-10-18

**Authors:** Elisabeth Dornisch, Jakob Pletz, Ronald A. Glabonjat, Florian Martin, Christian Lembacher‐Fadum, Margit Neger, Christoph Högenauer, Kevin Francesconi, Wolfgang Kroutil, Klaus Zangger, Rolf Breinbauer, Ellen L. Zechner

**Affiliations:** ^1^ Institute of Molecular Biosciences University of Graz Humboldtstrasse 50/I 8010 Graz Austria; ^2^ Institute of Organic Chemistry Graz University of Technology Stremayrgasse 9 8010 Graz Austria; ^3^ Institute of Chemistry University of Graz Heinrichstrasse 28 & Universitätsplatz 1 8010 Graz Austria; ^4^ Division of Gastroenterology and Hepatology Department of Internal Medicine Medical University of Graz Auenbruggerplatz 15 8036 Graz Austria; ^5^ BioTechMed-Graz Austria

**Keywords:** biosynthesis, gut bacteria, natural products, nonribosomal peptides, pyrrolobenzodiazepines

## Abstract

The nonribosomal enterotoxin tilivalline was the first naturally occurring pyrrolobenzodiazepine to be linked to disease in the human intestine. Since the producing organism *Klebsiella oxytoca* is part of the intestinal microbiota and the pyrrolobenzodiazepine causes the pathogenesis of colitis it is important to understand the biosynthesis and regulation of tilivalline activity. Here we report the biosynthesis of tilivalline and show that this nonribosomal peptide assembly pathway initially generates tilimycin, a simple pyrrolobenzodiazepine with cytotoxic properties. Tilivalline results from the non‐enzymatic spontaneous reaction of tilimycin with biogenetically generated indole. Through a chemical total synthesis of tilimycin we could corroborate the predictions made about the biosynthesis. Production of two cytotoxic pyrrolobenzodiazepines with distinct functionalities by human gut resident *Klebsiella oxytoca* has important implications for intestinal disease.

Antibiotic therapy disrupts the human intestinal microbiota. Shifts in microbial communities have been correlated to the pathogenesis of many disorders including metabolic diseases, cancer, and disorders of the liver, bowel, and lung.^1^, ^2^ Given the vast number of potentially bioactive substances produced by the gut microbiota, one of the greatest challenges facing researchers is to link a specific organism and its metabolites to a particular pathological outcome for the host.^3^, ^4^ However, only a few microbial small molecules have been shown to cause some of these clinically relevant phenotypes.^2^, ^3^, ^4^, ^5^, ^6^ The enteric bacterium *Klebsiella oxytoca* presents a striking example. In some patients treated with penicillin antibiotics, rapid proliferation of this organism results in antibiotic‐associated hemorrhagic colitis (AAHC).^7^ We showed in previous work that the indol‐3‐yl‐substituted pyrrolobenzodiazepine (PBD) tilivalline (**1**) produced by *K. oxytoca* was required for the pathogenesis of colitis in an animal model of AAHC.^8^ Naturally occurring PBDs form a family of antitumor antibiotics produced by Gram‐positive soil bacteria.^9^ Synthesis of a natural PBD by a Gram‐negative resident of the gut is thus surprising. We identified the toxin biosynthetic gene cluster by genetic mutation and localized the region to a unique pathogenicity island on the genome of cytotoxic *K. oxytoca* strains.^8^

Here we report the biosynthesis of tilivalline and characterize a second PBD monomer generated by this pathway, tilimycin, which has stronger cytotoxic properties. The toxin biosynthetic genes carried by the pathogenicity island are organized in two operons (Figure S1). BLAST analysis identified genes involved in synthesis of aromatic amino acids and related aromatic compounds: a 4‐hydroxyphenyl acetate‐3‐monoxygenase (*hmoX*), a 2‐amino‐2‐deoxyisochorismate synthase (*adsX*), an isochorismatase (*icmX*), a 2,3‐dihydro‐2,3‐dihydroxybenzoate dehydrogenase (*dhbX*), and a 2‐keto‐3‐deoxy‐d‐arabino‐heptolosonate phosphate synthase (*aroX*). The NRPS operon contains tilivalline‐specific nonribosomal peptide synthases *npsA*, *thdA*, and *npsB*. Our earlier mutagenesis studies showed that cluster genes *aroX*, *npsA+thdA*, and *npsB,* and a 3‐dehydroquinate synthase (*aroB*) at a distant genomic locus are essential for tilivalline biosynthesis.^8^ However, no genes involved in forming the indole ring at the C11 position of the tilivalline structure were apparent. *K. oxytoca* are indole producers predicted to use a tryptophanase to catalyze cleavage of l‐tryptophan to indole, pyruvate, and ammonium.^10^ We inactivated the putative tryptophanase gene (*tnaA*) of *K. oxytoca* AHC‐6. Wild‐type (WT) AHC‐6 and the Δ*tnaA* mutant were cultivated in vitro, and the presence of tilivalline in culture broth sampled during 48 h of growth was monitored by HPLC‐MS. In contrast to the *K. oxytoca* WT culture, no tilivalline was detected for the Δ*tnaA* mutant (Figure 1 A). Unexpectedly, however, spent medium of the mutant Δ*tnaA* showed cytotoxic activity on HeLa cells comparable to that of the tilivalline‐producing WT strain (Figure 1 B), which suggested that the Δ*tnaA* mutant might produce a different cytotoxic secondary metabolite. By extraction of the culture broth of the Δ*tnaA* mutant with butanol and subsequent purification via preparative HPLC, two previously unidentified small‐molecule metabolites were isolated and spectroscopically characterized. The two metabolites have identical molecular weight (234.3 g mol^−1^; C_12_H_14_N_2_O_3_). The first metabolite, which we named tilimycin (**2**), shares with tilivalline the same pyrrolo[2,1‐*c*][1,4]benzodiazepine motif but has an hydroxyl group instead of the indole ring at the C11 position resulting in a hemiaminal moiety, which is also responsible for the low stability of **2** (Figure 1 C). Tilimycin (**2**)^11^ is more cytotoxic to HeLa cells (IC_50_=2.6 μm) than tilivalline (**1**) (IC_50_=14.5 μm; Figure S6). The second new metabolite was isolated from culture broth of both WT and Δ*tnaA* mutant strains after incubation for >24 h. This product, which we named culdesacin (**3**), belongs to the class of pyrrolo[1,2‐*b*]isoquinolin‐5(1*H*)‐ones. Unlike **1** and **2**, **3** shows no cytotoxic activity (Figure S6).

**Figure 1:**
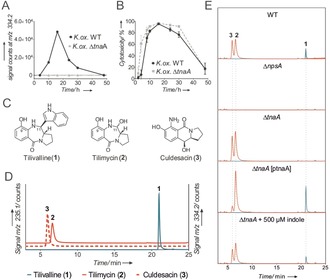
A) Time‐dependent formation of **1** in conditioned medium of *K*. *oxytoca* WT and *K*. *oxytoca* Δ*tnaA*, as measured by HPLC‐ESI‐MS (signal counts at *m*/*z* 334.2). B) Cytotoxicity to HeLa cells of medium conditioned by *K*. *oxytoca* AHC‐6 WT and *K*. *oxytoca* AHC‐6 Δ*tnaA* over time as indicated. C) Chemical structures of tilivalline (**1**), tilimycin (**2**), and culdesacin (**3**). D) HPLC‐ESI‐MS chromatograms of synthetic standards of tilivalline (**1**)—*m*/*z* 334.2, tilimycin (**2**), and culdesacin (**3**)—*m*/*z* 235.1. E) HPLC‐ESI‐MS chromatograms (red: *m*/*z* 235.1 and blue: *m*/*z* 334.2) of butanol extracts of 24 h cultures as indicated.

The three metabolites **1**–**3** were detected by HPLC‐ESI‐MS in butanol extracts of conditioned medium from *K. oxytoca* WT but not from the toxin‐negative *K. oxytoca* Δ*npsA*, where the specific nonribosomal peptide synthase NpsA is absent. The Δ*tnaA* mutant produced only **2** and **3**. Importantly, tilivalline biosynthesis was restored in the mutant by genetic complementation (*K*. *oxytoca* AHC‐6 Δ*tnaA* [pTnaA]) as well as by addition of 500 μm indole (Figure 1 E).

These findings led us to propose that tilivalline (**1**) may form spontaneously by nucleophilic addition of indole made available by tryptophanase at an imine intermediate in situ generated from **2** (Scheme 1).

**Scheme 1:**
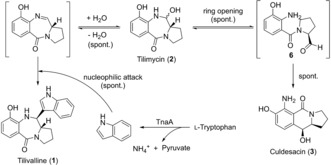
Proposed chemical reactions of tilimycin (**2**) to culdesacin (**3**) via spontaneous ring‐opening and to tilivalline (**1**) via a nucleophilic attack of free indole, released by the tryptophanase (TnaA)‐catalyzed cleavage of l‐tryptophan.

This could be verified by HPLC detection of tilivalline after synthetic tilimycin and indole had been added to medium lacking bacteria or enzyme activity (Figure S4). These experiments showed that spontaneous conversion of tilimycin (**2**)^12^ to culdesacin (**3**) occurs and that **2** reacts with indole to form tilivalline (**1**) as the natural *trans* isomer in the absence of enzyme activities (see Figure S8).

Having identified tilimycin (**2**) as the precursor for tilivalline (**1**), we next addressed the biosynthesis of **2**. The adenylation (A) domains of NpsA and NpsB were analyzed for substrate‐activating specificity. The NpsA/ThdA module is predicted to accept anthranilate substrates and NpsB l‐proline.^13^ The *aroX‐dhbX‐icmX‐adsX‐hmoX* operon may provide the anthranilic substrate via enzymes related to the shikimate and chorismate pathways (Scheme 2) similar to the biosynthesis of the analogous PBDs anthramycin and tomaymycin.^14^ The 2‐keto‐3‐deoxy‐d‐arabino‐heptolosonate phosphate synthase AroX is independent of amino acid feedback regulation like its counterpart TomC in tomaymycin synthesis.^14^, ^15^ AroX and the 3‐dehydroquinate synthase AroB are involved in chorismate synthesis and are essential for tilivalline biosynthesis.^8^ Chorismate can be converted by AdsX to 2‐amino‐2‐deoxyisochorismate, as was shown for analogues TomD in tomaymycin biosynthesis and PhzE in phenazine biosynthesis.^15^, ^16^ We wondered whether 3‐hydroxyanthranilic acid (3HAA) is the substrate of NpsA/ThdA. Two pathways for production of this precursor from 2‐amino‐2‐deoxyisochorismate (ADIC) are conceivable (Scheme 2). In the first pathway, based on phenazine biosynthesis, ADIC is converted to *trans*‐2,3‐dihydro‐3‐hydroxyanthranilic acid (DHHA) by the isochorismatase IcmX, which is 40 % identical to isochorismatase PhzD of *Pseudomonas aeruginosa* PAO1 (NP_252903.1).^17^ The oxidation of DHHA to 3HAA could be catalyzed by 2,3‐dihydro‐2,3‐dihydroxybenzoate dehydrogenase DhbX (Scheme 2, route a). The second pathway is based on tomaymycin biosynthesis where chorismate conversion to anthranilic acid (AA) requires the 2‐amino‐2‐deoxyisochorismate synthase (TomD) and the anthranilate synthase (TomP).^15^

**Scheme 2:**
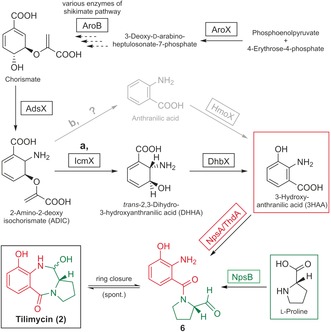
Proposed biosynthetic pathways of tilimycin (**2**) via 3‐hydroxyanthranilic acid (substrate of NpsA/ThdA) and l‐proline (substrate of NpsB). AroX: 2‐keto‐3‐deoxy‐d‐arabino‐heptolosonate phosphate (DHAP) synthase; AroB: 3‐dehydroquinate synthase; AdsX: 2‐amino‐2‐deoxyisochorismatase; IcmX: isochorismatase; DhbX: 2,3‐dihydro‐2,3‐dihydroxybenzoate dehydrogenase; HmoX: 4‐hydroxyphenyl acetate‐3‐monoxygenase; NpsA/ThdA: nonribosomal peptide synthase; NpsB: nonribosomal peptide synthase.

Hydroxylation of AA to 3HAA could be catalyzed by a 4‐hydroxyphenylacetate‐3‐monoxygenase as it was shown for GTNG 3160 in *Geobacillus thermodenitrificans* NG80‐2 (Scheme 2, route b).^18^ *hmoX* and a second homologue on the AHC‐6 genome encode 4‐hydroxyphenylacetate‐3‐monoxygenases 32 % identical to GTNG_3160. To date, the *K. oxytoca* genome lacks an annotated anthranilate synthase.

To test these pathways, *K. oxytoca* AHC‐6 single‐gene mutants Δ*aroB,* Δ*aroX,* Δ*adsX,* Δ*icmX,* Δ*dhbX* were cultured in CASO medium or medium supplemented with either 3HAA or AA. *K. oxytoca* WT and *K. oxytoca* Δ*npsA* were included as controls. The conditioned medium of these experiments was then extracted and analyzed by HPLC for the amounts of **1**–**3**. Strains lacking NpsA, AroB, AroX, AdsX, IcmX, and DhbX were functionally deficient for the biosynthesis of all three metabolites. By contrast, deletion of *hmoX* and the genomic homologue (ΔΔ*hmoX*) had no effect. The observed deficiencies were complemented chemically by addition of 3HAA to culture medium in every case, except for the Δ*npsA* control strain. The addition of AA to mutant cultures did not restore production of **2** or **1** but instead their respective deoxy derivatives 9‐deoxytilimycin (**4**) and 9‐deoxytilivalline (**5**) (Figure 2 A,B). All five metabolites could be detected after addition of AA to the WT strain, whereas none were observed for control strain *K. oxytoca* Δ*npsA* in all cases. Deoxyculdesacin was not detected, presumably due to the lack of the aromatic hydroxyl group, which can be considered essential for nucleophilic attack of the arene at the aldehyde electrophile.

**Figure 2:**
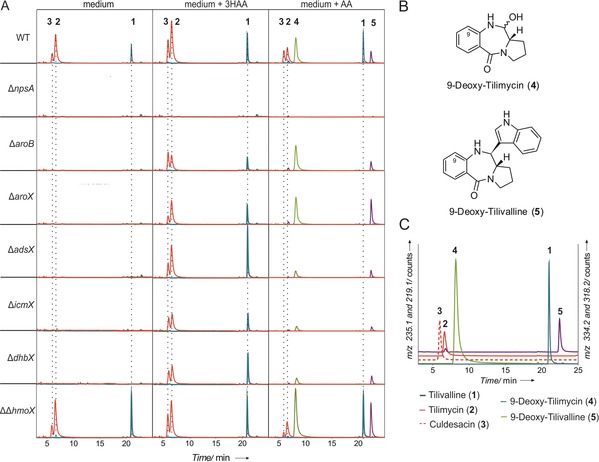
Feeding experiments with *K*. *oxytoca* mutant strains confirm synthesis of tilimycin (**2**) via 3‐hydroxyanthranilic acid (3HAA). The addition of synthetic anthranilic acid (AA) led to the mutasynthesis of 9‐deoxytilimycin (**4**) and 9‐deoxytilivalline (**5**). A) HPLC‐ESI‐MS chromatograms (red: *m*/*z* 235.1, blue: *m*/*z* 334.2, green: *m*/*z* 219.1, violet: *m*/*z* 318.2) of *n*‐butanol extracts of conditioned medium (24 h culture) from *K*. *oxytoca* AHC‐6 WT, Δ*npsA*, Δ*aroB*, Δ*adsX*, Δ*icmX*, Δ*dhbX*, ΔΔ*hmoX* grown in medium (left column), and in medium supplemented with 3HAA (middle column) or with AA (right column). B) Chemical structures of **4** and **5**. C) HPLC‐ESI‐MS chromatograms of synthetic standards of (**1**)—*m*/*z* 334.2 (blue), (**2**) and (**3**)—*m*/*z* 235.1 (red), (**4**)—*m*/*z* 219.1 (green), (**5**)—*m*/*z* 318.2 (violet).

In conclusion, by a combination of metabolite profiling, genetic deletion experiments, and complementation experiments with synthetically prepared intermediates, we established the biosynthetic pathway of tilimycin (**2**) and tilivalline (**1**) (Scheme 3). 3HAA, synthesized from chorismate by enzymes from the pathogenicity island, is processed by a nonribosomal peptide synthase to *N*‐acylprolinal **6**. The subsequent ring closure to tilimycin (**1**) as well as the introduction of the indole moiety do not require a specific enzyme but can occur spontaneously through the intrinsic reactivities of the pertinent reaction intermediates. We could show that the biosynthetic pathway can be exploited for mutasynthesis of unnatural pyrrolobenzodiazepine through the addition of anthranilic acid and indole derivatives.

**Scheme 3:**
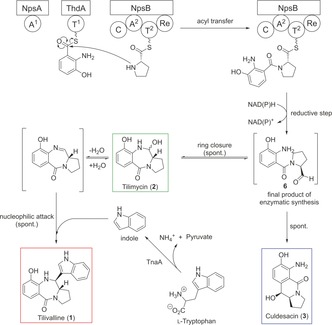
Complete biosynthesis of tilivalline (**1**) via tilimycin (**2**). After binding and activation of 3‐hydroxanthranilic acid and l‐proline to the nonribosomal peptide synthases NpsA/ThdA and NpsB, the reductive release to an open *N*‐acylprolinal (**6**) occurs. The final product of enzymatic synthesis leads to either tilimycin (**2**) or culdesacin (**3**). Tilimycin (**2**) can be further converted to tilivalline (**1**) after the nucleophilic attack of free indole, which is released by the bacterial tryptophanase (TnaA) after enzymatic cleavage of l‐tryptophan.

The PBD family of potent cytotoxic agents has been extensively investigated for use in systemic chemotherapy.^5^ PBD monomers target purine–guanine–purine motifs in the minor groove of DNA. Once situated in the minor groove, an aminal bond is formed between the C11 position of the PBD and the N2 of guanine. The presence of the indole ring at the C11 position of tilivalline (**1**) blocks this activity, but tilimycin (**2**) is expected to belong to the DNA‐interacting antitumor agents. Thus *K. oxytoca* is able to produce two PBD cytotoxins with distinct functionalities depending on the availability of indole.

Hydroxylation at position C9 has been associated with cardiotoxicity for the model antitumor agents anthramycin and sibiromycin.^19^ The capacity for *K. oxytoca* to produce 9‐deoxytilimycin (**4**) when cultivated with AA reveals a promising approach to introduce structural modifications that enhance the biological activity and potency of anticancer analogues.

Finally, the results of this study are particularly significant in a physiological context. Since the feces of healthy humans usually contain 1.0 mm to 4.0 mm indole,^20^ we anticipate that both **1** and **2** are present in the intestine and exert distinct cytotoxic functionalities that contribute specifically to AAHC and possibly other disorders.

## Conflict of interest

The authors declare no conflict of interest.

## Supporting information

As a service to our authors and readers, this journal provides supporting information supplied by the authors. Such materials are peer reviewed and may be re‐organized for online delivery, but are not copy‐edited or typeset. Technical support issues arising from supporting information (other than missing files) should be addressed to the authors.

Supplementary
