# Supplementary material for: Biosynthesis of the Enterotoxic Pyrrolobenzodiazepine Natural Product Tilivalline
Source: Angew Chem Int Ed Engl. 2017 Oct 18;56(46):14753–7. doi: 10.1002/anie.201707737 (PMC5698749; doi:10.1002/anie.201707737)
Supplement: Supplementary file 1 — Supplementary [file ANIE-56-14753-s001.pdf]

Angewandte Chemie
Eine Zeitschrift der Gesellschaft Deutscher Chemiker

Supporting Information

Biosynthesis of the Enterotoxic Pyrrolobenzodiazepine Natural Product Tilivalline

Elisabeth Dornisch, Jakob Pletz, Ronald A. Glabonjat, Florian Martin, Christian Lembacher-Fadum, Margit Neger, Christoph Högenauer, Kevin Francesconi, Wolfgang Kroutil, Klaus Zangger, Rolf Breinbauer, and Ellen L. Zechner**

anie_201707737_sm_miscellaneous_information.pdf

Supporting Information

TABLE OF CONTENTS

TABLE OF CONTENTS 1

SI-1. Bacterial genetics 2

SI-2. Isolation and characterization of metabolites of *K.oxytoca* AHC-6 strains 6

SI-3. Total Synthesis of *cis*/*trans*-tilimycin (2) 12

SI-4. Total Synthesis of tilivalline (1) 19

SI-5. Total Synthesis of *cis*/*trans*-deoxy-tilimycin (4) 29

SI-6. Feeding experiments for confirmation of biosynthetic pathway 33

SI-7. Cell culture assay 39

SI-8. NMR and ATR-IR spectra 40

References 58

SI-1. Bacterial genetics

Bacterial culture techniques

E. coli and *K. oxytoca* strains were grown in LB- and CASO-bouillon, respectively, at 37 °C under aerobic conditions with shaking at 180 rpm. Plasmid-containing strains were grown with antibiotic selection using kanamycin (50 µg/mL), tetracycline (8 µg/mL) or chloramphenicol (10 or 30 µg/mL for *E. coli* or *K. oxytoca*, respectively).

Table S1: Bacterial strains and plasmids

Strains	Genotype	Reference
E.coli **DH5α**	*endA1 recA1 gyrA96 thi-l hsdR17 supE44 λ- relA1 deoR* ΔU169 ϕ80d*lacZ* Δ (M15)	[1]
E.coli **CSH26Cm::LTL**	CSH26 *galK::cat::loxP-TetRA-loxP*	[2]
K. oxytoca **AHC-6 WT**		[3]
K. oxytoca **AHC-6 *ΔnpsA***	*ΔnpsA*	[4]
K. oxytoca **AHC-6 Mut89**	KanR; *ΔnpsB*::Tn5(Transposon mutant Mut89)	[4]
K. oxytoca **AHC-6 *ΔaroX***	KanR; *ΔaroX*::Tn5 (Transposon mutant Mut2)	[4]
K. oxytoca **AHC-6 *ΔaroB***	Mut614; KanR; *ΔaroB*::Tn5 (Transposon mutant Mut614)	[4]
K. oxytoca **AHC-6 *ΔtnaA***	TetR; *ΔtnaA*::*tetA*/R	This study
K. oxytoca **AHC-6 *ΔadsX***	TetR; *ΔadsX*::*tetA*/R	This study
K. oxytoca **AHC-6 *ΔicmX***	KanR; *ΔicmX*::*aphA*	This study
K. oxytoca **AHC-6 *ΔdhbX***	KanR; *ΔdhbX*::*aphA*	This study
K. oxytoca **AHC-6 *ΔhmoX***	TetR; *ΔhmoX*::tetA/R	This study
K. oxytoca **AHC-6 *ΔΔhmoX***	TetR, KanR; *ΔhmoX*::*tetA*/R; *ΔhmoX*::*aphA*	This study
**Plasmids**	**Description**	**Reference**
**pKOBEG**	λ phage redγβα operon, ts replicon, CmR	[5]
**pBluescript KS(-)**	AmpR, lacZ; (cloning vector for recombination cassettes)	Stratagene
**pACYC184**	CmrR, TetR, p15A origin	[6]
**pACYC184-*tnaA***	CmR, *TetR::tnaA*, p15A origin	This study

Table S2: Oligonucleotides for gene disruption, complementation and sequencing

Olignucleotides	Sequence (5' → 3')
**Gene disruption**	
**HindIII_rrnB_fw**	CGACTAAGCTTGGCTGTTTTGGCGGATG
**SalI_rrnB_rev**	GCTTAGTCGACCAAAAGAGTTTGTAGAAACGCA
**SalI_TetR_fw**	GTATAGTCGACCAAGAATTGCCGGCGGAT
**SalI_TetR_rev**	GCTTAGTCGACGGTATTTCACACCGCATAGC
**SacI_Tryp_up_fw**	GCTTAGAGCTCCATCATTATGAACGTCTTTT
**HindIII_Tryp_up_rev**	ATGCTAAGCTTTTATTTTCTCCATGGGA
**XhoI_Tryp_dw_fw**	GCTTACTCGAGGATATGGAGGCGCTGGCTAT
**KpnI_Tryp_dw_rev**	ATGCTGGTACCTTATTTCACCGGTTTCAGT
**SalI_aphA_fw**	AGTCGTCGACCATGGCGATAGCTAGACTGG
**SalI_aphA_rev**	AGTCGTCGACCGAAGAACTCCAGCATGAG

Olignucleotides	Sequence (5' → 3')
**Gene disruption**	
**KpnI-adsX_dw-fw**	GACATGGTACCTTAAACGGCATCTTGTTGGC
**SalI-adsX_dw-rev**	CATATGTCGACCCTGGTATGACTGTCCGAGC
**HindIII-adsX_up-fw**	GTGTCAAGCTTATCATCATCTCCTGAAGTGC
**SacI-adsX_up-rev**	TATGAGAGCTCGGTCATCGACAACATCTACC
**SacI -IcmX_up -fw**	ATAGAGCTCCAGGATTGGGCGAAAACATTT
**HindIII-IcmX_up- rev**	ATAAAGCTTGTTATTCTCCTTGTACGCTTAGCAGC
**SalI-IcmX_dw- fw**	ATAGTCGACAGCGCTTGAGCTAGCTGCG
**KpnI-IcmX_dw -rev**	ATAGGTACCGTGCAGGTTTCGTGGCTTGA
**SacI-dhbX_up-fw**	ATAGAGCTCCCTGCAAGATTGACGCCAG
**HindIII-dhbX_up- rev**	ATAAAGCTTTCAAAAGTCATGTTGTCCCTCTTTC
**SalI-dhbX_dw-fw**	ATAGTCGACCGTTATTGGTCGACGGTGGT
**KpnI-dhbX_dw-rev**	ATAGGTACCAGAGGAGAGTTAAAGAAGGCGCTG
**SacI-hmoX_up-fw**	GCTAAGAGCTCGGCGATCCAACCAATATACA
**HindIII-hmoX_up-rev**	CGACTAAGCTTGTCAGCTCTCTCCGAATGTC
**SalI-hmoX_dw-fw**	GCTTAGTCGACCAACCTCTTCTGGGCCATAA
**KpnI-hmoX_dw-rev**	GCTAAGGTACCCTAAAGGCCAGTGGTAATCG
**SacI-hmoXg_up-fw**	ATATGAGCTCGCTGCTATTTTCCGACAAC
**HindIII-hmoXg_up-rev**	GATCAAGCTTGGTGACCTCTTCAGTATTAATCG
**SalI-hmoXg_dw-fw**	ATCTGTCGACAATATGTTCTGGGCGCTG
**KpnI-hmoXg_dw-rev**	AGCGGGTACCTTACTTCAGCAGATTATCCAGC
**Complementation**	
**BamHI_Tryp_fw**	CGAATGGATCCTGTAAATTCCCATGGAGAAAATAA
**SalI_Tryp_rev**	ATGTAGTCGACTTATTTCACCGGTTTCAGT
**Sequencing**	
**TrypTrans_out_rev**	CAGCGATGGTGTATTAGTTGAGACCGC
**Ethidium_out_fw**	CGTCACGATTTGCTCGATGA
**Seq_adsXRekomb-fw**	CATTACCGGTAGGTAATGATACTG
**Seq_adsXRekomb-rev**	GCTACTCATACATGATATGCAGC
**Seq_icmXRekomb-fw**	CCTTTACCGCAGTCGGTCAT
**Seq_icmXRekomb-rev**	CTCCAGGCTGCGTGAAATC
**Seq_dhbXRekomb-fw**	GGCCCTGGATAGGCATTCGT
**Seq_dhbXRekomb-rev**	CGCTGTGCACAAACAACCGA
**Seq_hmoXRekomb-fw**	CCTGGTATGACTGTCCGAGC
**Seq_hmoXRekomb-rev**	CTAAAGGCCAGTGGTAATCG
**Seq_hmoXgRekomb-fw**	ATAGCCACTACCACCAGCACTG
**Seq_hmoXgRekomb-rev**	CGTATCAGTCACCGAACAGACC

Gene inactivation with the lambda red recombination system

Genes of interest on the *K. oxytoca* AHC-6 chromosome were disrupted using the lambda red recombination enzymes expressed by the pKOBEG vector. In each case a linear recombination cassette - containing a selection marker gene (*tetR* or *aphA*) flanked by homology arms (~500 bp) of the target gene replaced the wild type. The gene disruption cassettes were cloned in pBluescript II KS(-) vector using the strategies described below.

For mutagenesis, competent *K. oxytoca* cells containing pKOBEG were induced with 0.2 % arabinose and transformed with 100 ng of PCR-amplified linear recombination fragment after DpnI digestion. Transformants were selected with 8 µg/mL tetracycline or 50 µg/mL kanamycin and incubated at 32 °C overnight, then cured of the temperature-sensitive pKOBEG by growth at 42 °C. Successful disruption of the bacterial gene was verified via PCR and DNA sequencing.

Gene disruption in *K. oxytoca* AHC-6

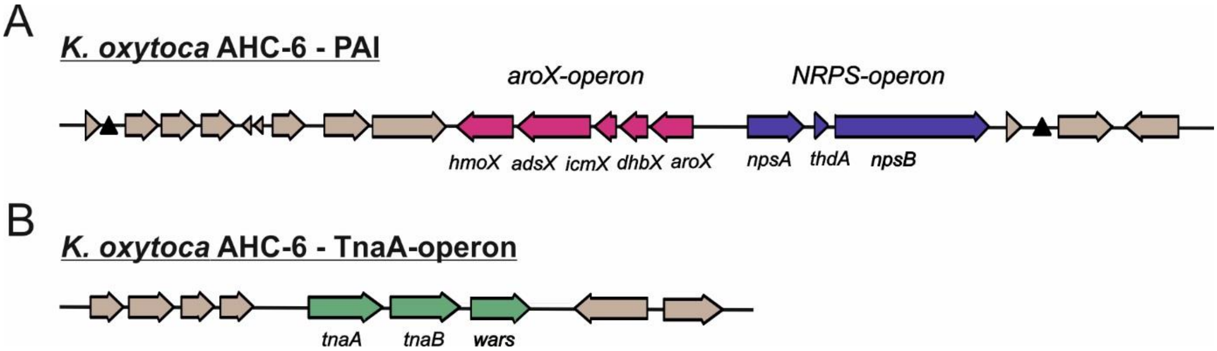

Figure S1. (A) Location and orientation of putative biosynthesis genes from the aroX-operon (pink): *hmoX:* 4-hydroxyphenyl acetate-3-monoxygenase, *adsX*: 2-amino-2-deoxy-isochorismate synthase, *icmX*: isochorismatase, *dhbX*: 2,3-dihydro-dehydrogenase, *aroX*: 2-keto-3-deoxy-D-arabino-heptolosonate phosphate synthase; and the tilivalline specific non-ribosomal peptide synthases *npsA*, *thdA* and *npsB* of the adjacent NRPS-operon (purple) on the *K. oxytoca* pathogenicity island (PAI) (Accession no.: HG425356). (B) Organization of genes from the *tnaA*-operon (green) on the chromosome of *K. oxytoca* AHC-6; *tnaA* (tryptophanase), *tnaB* (tryptophan permease) and *wars* (tryptophanyl-tRNA synthetase).

Our previous study showed that *aroX*, *npsA*, *thdA* and *npsB* are essential for tilivalline biosynthesis.[4] In this study the remaining genes *dhbX*, *icmX*, *adsX* and *hmoX* and the chromosomal tryptophanase gene *tnaA* were inactivated to elucidate their role in tilivalline biosynthesis (Figure S1). A double *hmoX* mutant disrupted the PAI gene and a homologous gene on the AHC-6 chromosome.

Recombination cassettes

***ΔtnaA*:** 500 bp of sequence upstream of the start codon of the *tnaA* gene was amplified with SacI_Tryp_up_fw and HindIII_Tryp_up_rev from genomic DNA and inserted upstream of a tetracycline resistance cassette (*tetA/R*) amplified from the *E. coli* CSH26Cm::LTL chromosome (SalI_TetR_fw and SalI_TetR_rev) and a *rrnB* terminator sequence amplified from pBAD18-Kan (HindIII_rrnB_fwd and SalI_rrnB_rev). The downstream flank (amplified with XhoI_Tryp_dw_fw and KpnI_Tryp_dw_rev) corresponds to the last 500 bp inside the adjacent *tnaB* coding sequence to preserve possible promoter sequences for the genes downstream of *tnaA*.

***ΔadsX*:** Both 500 bp recombination flanks were amplified from the genome (HindIII-adsX_up-fw and SacI-adsX_up-rev and KpnI-adsX_dw-fw plus SalI-adsX_dw-rev) and inserted up- or downstream of *tetA/R* and a *rrnB* terminator sequence.

***ΔicmX*:** Homology arms were amplified (SacI-IcmX_up-fw and HindIII-IcmX_up-rev and SalI-IcmX_dw-fw plus Kpn-IcmX_dw-rev) and inserted up- or downstream of a kanamycin resistance cassette (*aphA*) amplified from pAR80 (SalI_aphA_fw and SalI_aphA_rev) and a *rrnB* terminator sequence.

***ΔdhbX*:** Both 500 bp recombination flanks were amplified from the genome (SacI-dhbX_up-fw and HindIII-dhbX_up-rev and SalI-dhbX_dw-fw plus Kpn-dhbX_dw-rev) and inserted up- or downstream of *aphA* and an *rrnB* terminator sequence.

***ΔΔhmoX*:** For the PAI *hmoX* homology arms were amplified from the genome (SacI-hmoX_up-fw and HindIII-hmoX_up-rev and SalI-hmoX_dw-fw plus Kpn-hmoX_dw-rev) and inserted up- or downstream of *tetA/R* and a *rrnB* terminator sequence. For the second *hmoX* homologue the 500 bp recombination flanks were amplified (SacI-hmoXg_up-fw and HindIII-hmoXg_up-rev and SalI-hmoXg_dw-fw plus Kpn-hmoXg_dw-rev) and inserted up- or downstream of *aphA* and an *rrnB* terminator sequence.

SI-2. Isolation and characterization of metabolites of *K.oxytoca* AHC-6 strains

General Information

Analytical thin layer chromatography (TLC) was carried out on Merck TLC silica gel aluminum sheets (silica gel 60, F_{254}, 20 x 20 cm) and spots were visualized by UV light (λ = 254 nm and/or λ = 366 nm) and by staining with cerium ammonium molybdate solution (50 g $(NH_4)_6Mo_7O_{24}$ were dissolved in 400 mL H_2O and 50 mL conc. H_2SO_4 was added followed by 2.0 g $Ce(SO_4)_2$) and developed by heating with a heat gun.

Column chromatography was performed on silica gel 60 from Acros Organics with particle sizes 35-70 µm. A 30- to 100-fold excess of silica gel was used with respect to the mass of dry crude product, depending on the separation problem. The crude material was dissolved in MeOH and subsequently adsorbed on the 2.5-fold excess of Celite™. Afterwards the solvent was removed in vacuum and the adsorbed crude material was dried in oil pump vacuum. The dimension of the column was adjusted to the required amount of silica gel and formed a pad between 25 cm and 40 cm. In general, the silica gel was mixed with the eluent and charged into the column before equilibration. For smaller column dimensions the dry silica gel was filled into the column and was equilibrated by forcing an appropriate amount of eluent through by over-pressure. Subsequently, the dissolved or adsorbed crude material was loaded onto the top of the silica gel and the mobile phase was forced through the column by pressure exerted by a rubber bulb pump. The volume of each collected fraction was adjusted between 20 % and 30 % of the silica gel volume, according to the separation problem.

Instrumentation

^{1}H- and ^{13}C-NMR spectra were recorded on a Bruker AVANCE III 300 spectrometer (^{1}H: 300.36 MHz; ^{13}C: 75.53 MHz) with autosampler, a Varian Unity Inova 500 spectrometer (^{1}H: 499.88 MHz; ^{13}C: 125.69 MHz) and on a Bruker Avance III 700 MHz NMR spectrometer using a cryogenically cooled 5 mm TXI probe with z-axis gradients at 298 K. Chemical shifts were referenced to the residual proton and carbon signal of the deuterated solvent (DMSO-d_6: δ = 2.50 ppm (^{1}H), 39.52 ppm (^{13}C); MeOH-d_4: δ = 3.31 (^{1}H), 49.00 ppm (^{13}C)). Chemical shifts δ are given in ppm (parts per million) and coupling constants *J* in Hz (Hertz). 1D spectra (APT and NOESY) as well as 2D spectra (HH-COSY, HSQC and HMBC) were recorded for the identification and confirmation of the structure. Signal multiplicities are abbreviated as s (singlet), bs (broad singlet), d (doublet), dd (doublet of doublet), ddd (doublet of doublet of

doublet), td (triplet of doublet), t (triplet), dt (doublet of triplet) and m (multiplet). Additionally, quarternary carbon atoms are designated as C_q and aromatic carbon atoms bearing a hydrogen as CH_{arom}. Deuterated solvents for nuclear resonance spectroscopy were purchased from Roth, Armar Chemicals and Euriso-top®.

Melting points were determined on a Mel-Temp® melting point apparatus from Electrothermal with an integrated microscopical support. They were measured in open capillary tubes with a mercury-in-glass thermometer and were not corrected.

IR-spectra were recorded neat on a Bruker Alpha-P (ATR) instrument.

The specific optical rotation was determined on a Perkin Elmer Polarimeter 341 with an integrated sodium vapor lamp. All samples were measured in MeOH (Fluka, HPLC grade) at the D-line of the sodium light (λ = 589 nm) under non-tempered conditions between 22 °C and 27 °C.

Purification via preparative RP-HPLC of tilimycin (**2**), culdesacin (**3**) and 9-deoxy-tilimycin (**4**) was performed on a Thermo Scientific Dionex UltiMate 3000 system with UltiMate 3000 pump, UltiMate 3000 autosampler, UltiMate 3000 column compartment, UltiMate 3000 diode array detector (deuterium lamp, λ = 190–380 nm) and a UltiMate 3000 automatic fraction collector. The components were separated on a RP Macherey-Nagel 125/21 Nucleodur® 100-5 C18ec column (21 × 125 mm, 5.0 µm). Signals were detected at 210 nm and 254 nm. As mobile phase acetonitrile (VWR HiPerSolv, HPLC grade) and water (Barnstead NANOpure®, ultrapure water system) were used. The following method was used (Method A): column oven 26 °C, flow rate 15 mL/min; 0.0–7.0 min MeCN/H_2O = 2:98 (v/v), 7.0–82.0 min linear increase to MeCN/H_2O = 50:50 (v/v), 82.0–90.0 min linear increase to MeCN/H_2O = 95:5 (v/v,), 90.0 min–100.0 min hold MeCN/H_2O = 95:5 (v/v).

Isolation of *cis/trans*-tilimycin (2) from conditioned medium

K. oxytoca AHC-6 *ΔtnaA* was cultivated in 100 mL CASO medium in 300 mL Erlenmeyer flask (40x flasks were prepared in parallel) at 37 °C with orbital shaking at 180 rpm for 16 h. A total volume of 4 L culture broth was centrifuged at 8000 rpm for 30 min at 4 °C. The supernatant was filtered through a 0.45 µm cellulose-acetate filter followed by a 0.2 µm cellulose-acetate filter to remove residual bacterial cells. Metabolites were extracted from the conditioned medium with *n*-butanol (≥99.5%, for synthesis, Roth) applying a 3:2 mixing

ratio (two times). The organic extract was concentrated to dryness under high vacuum at 40 °C and was adsorbed on Celite™ and then purified via flash column chromatography (150 g silica gel, size: 420 x 30 mm, CH_2Cl_2/MeOH = 20:1 (v/v)). The obtained crude product (109 mg, colorless solid) was further purified by preparative RP-HPLC (Method A), followed by a second flash column chromatography (10 g silica gel, size: 210 x 11 mm, CH_2Cl_2/MeOH = 20:1 (v/v)).

Yield: 40.3 mg (172 µmol), off-white solid.

$C_{12}H_{14}N_2O_3$ [234.26 g/mol].

mp = 78–85 °C (decomp.).

$[\alpha]_D^{23}$ = +256 (c = 0.10, MeOH).

R_f = 0.31 (CH_2Cl_2/MeOH = 20:1 (v/v); CAM: orange).

IR (ATR): 2951, 2874, 2368, 2329, 2248, 2204, 1597, 1557, 1480, 1431, 1358, 1302, 1261, 1208, 1133, 1108, 1074, 1056, 979, 922, 897, 875, 852, 818, 744, 629, 607, 554, 493, 443, 418 cm^{-1}.

HR-MS (ESI): *m/z* $[M+H]^+$ calcd for $C_{12}H_{15}N_2O_3^+$: 235.1077; found: m/z 235.1069 (Δ = -0.8 mmu)

NMR-data of *cis/trans*-tilimycin (2)

Natural tilimycin (**2**) was obtained as an inseparable ~1:1 mixture of the *cis*- and *trans*-isomer. The assignment of the chemical shifts to the atoms in the mixture was made by 1D and 2D experiments (HSQC, HMBC) (Full spectra SI-8). Differentiation between the *cis*- and the *trans*-isomer of tilimycin (**2**) was obtained by NMR analysis of the *J*-coupling between H-8 to H-9. Only the *trans*-isomer gives a doublet (8.8 Hz) due to coupling between H-8 and H-9, whereas the protons in the *cis*-isomer show no coupling (Figure S2). Additional confirmation about the *cis*/*trans* geometry came from a 2D NOESY experiment. The F1 traces of protons H8/H8' are shown in Figure S3. The *trans*-isomer (H8') shows a stronger NOE to H9', while in the *cis*-isomer (H8) a stronger NOE to H10b is observed.

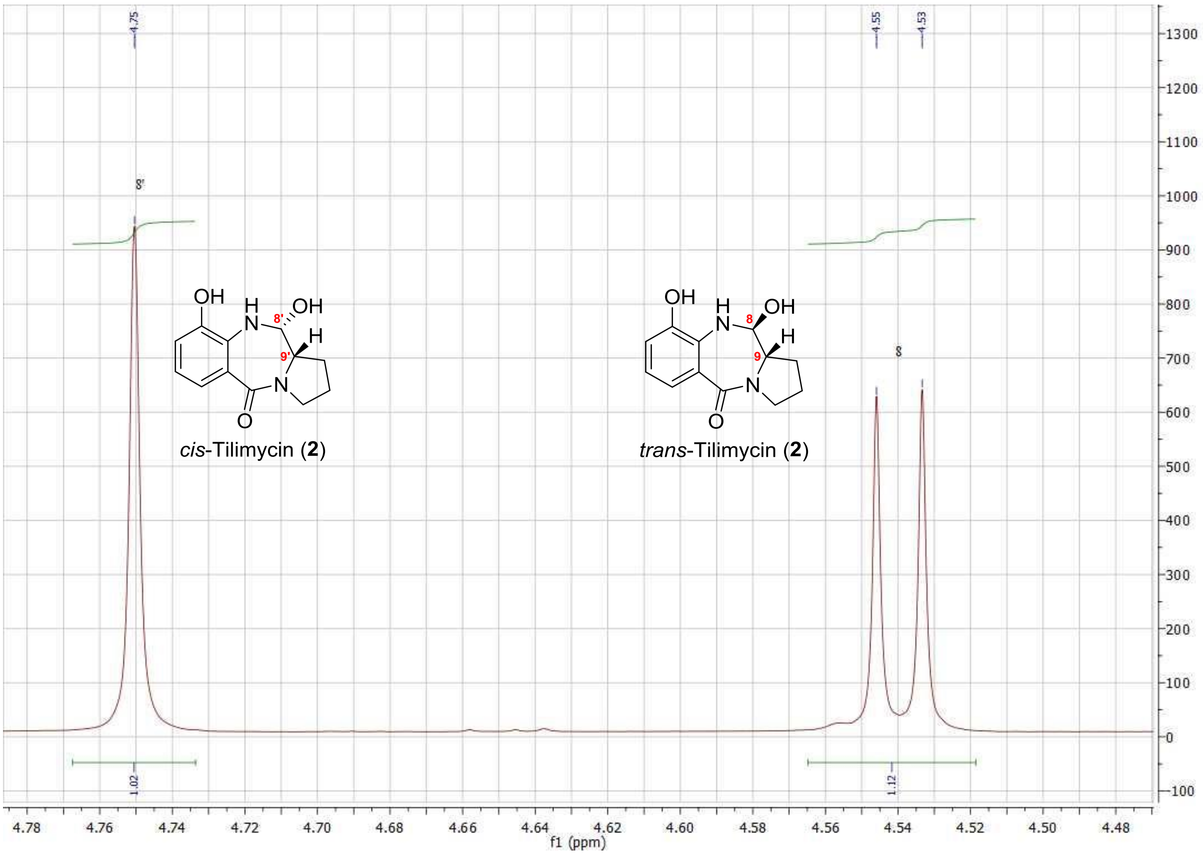

Figure S2: Enlargement of the 1H-NMR spectrum of natural *cis*/*trans*-tilimycin (**2**).

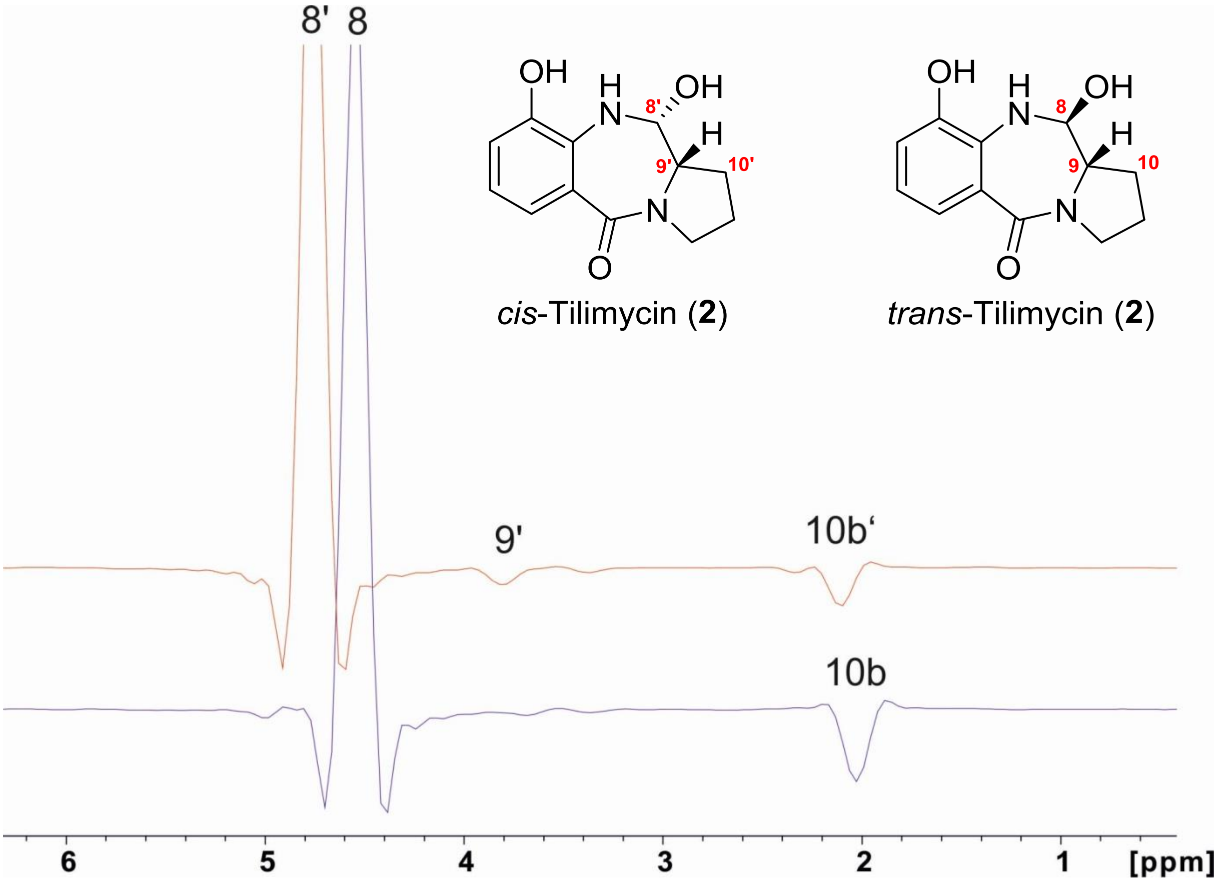

Figure S3: F1 traces of a 2D NOESY spectrum of natural *cis*/*trans*-tilimycin (**2**) taken at the H8 and H8' signals.

The ^{1}H- and ^{13}C-NMR signals of the *cis*- and *trans*-isomer of tilimycin (**2**) were assigned by their connectivity to the previously determined H-8 atoms (8 and 8') by 2D NMR experiments (HSQC, HMBC).

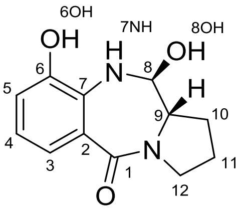

trans-Tilimycin (**2**)

Table S3: NMR data (^{1}H 700 MHz, ^{13}C 175 MHz) of the *trans*-isomer of natural tilimycin (**2**) in MeOD-d$_4$.

Atom #	δ_C, mult.	δ_H, mult. (Hz)
1	170.6, C_q	
2	130.2, C_q	
3	120.9, CH_{arom}	7.11, dd (7.7, 1.5)
4	123.1, CH_{arom}	6.87, t (7.8)
5	117.8, CH_{arom}	6.91, dd (7.9, 1.5)
6	150.2, C_q	
7	133.3, C_q	
8	96.8, CH	4.54, d (8.8)
9	60.6, CH	3.58-3.47, m
10	30.3, CH_2	2.21-1.98, m
11	23.8/23.9, CH_2	2.21-1.98, m
12	47.8, CH_2	3.74-3.67, m<br>3.58-3.47, m

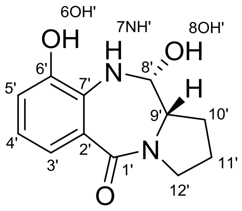

cis-Tilimycin (**2**)

Table S4: NMR data (^{1}H 700 MHz, ^{13}C 175 MHz) of *cis*-isomer of natural tilimycin (**2**) in MeOD-d$_4$.

Atom #	δ_C, mult.	δ_H, mult. (Hz)
1’	169.2, C_q	
2’	119.4, C_q	
3’	123.6, CH_{arom}	7.31, dd (8.2, 1.4)
4’	117.7, CH_{arom}	6.58, t (7.9)
5’	116.2, CH_{arom}	6.81, dd (7.6, 1.4)
6’	146.0, C_q	
7’	134.7, C_q	
8’	89.8, CH	4.75, s
9’	60.6, CH	3.79, dd (8.3, 4.9)
10’	31.6, CH_2	2.35-2.28, m<br>2.21-1.98, m
11’	23.8/23.9, CH_2	2.21-1.98, m<br>1.86-1.78, m
12’	50.3, CH_2	3.74-3.67, m

SI-3. Total Synthesis of *cis*/*trans*-tilimycin (2)

3-Hydoxy isatoic anhydride

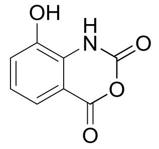

A dry 20 mL Schlenk tube with magnetic stirring bar was charged with 250 mg (1.63 mmol, 1.0 eq) 3-hydroxyanthranilic acid and 5.0 mL THF abs. in an Ar counter-stream. 165 mg (555 µmol, 0.3 eq) triphosgene were added to the red suspension. The reaction mixture was heated to 45 °C (oil bath) and stirred for 1.5 h until TLC indicated full conversion. The gray suspension was concentrated under reduced pressure and 10 mL *n*-hexane was added. The precipitate was collected by filtration and dried in vacuum to obtain the crude title compound as a grayish-red powder, which was used without further purification.[7]

Yield: 263 mg (1.47 mmol, 90 %), grayish-red powder.

$C_8H_5NO_4$ [179.13 g/mol].

(*S*)-(2-Amino-3-hydroxyphenyl)(2-(hydroxymethyl)pyrrolidin-1-yl)methanone

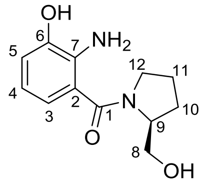

A dry 15 mL Schlenk tube with magnetic stirring bar was charged with 243 mg (1.36 mmol, 1.0 eq) 3-hydroxy isatoic anhydride and 2.2 mL DMSO abs. in an Ar counter-stream. After addition of 147 µL (1.49 mmol, 1.1 eq) L-prolinol, the brown solution was stirred for 16 h at 100 °C. The solvent was removed in oil-pump vacuum at elevated temperatures (50-60 °C). The obtained crude product was purified via flash column chromatography (100 g silica gel, size: 140 x 50 mm, CH_2Cl_2/MeOH = 20:1 (v/v)).

Yield: 148 mg (625 µmol, 46%), brown solid.

$C_{12}H_{14}N_2O_3$ [234.26 g/mol].

mp = 60-62 °C.

$[\alpha]_D^{23}$ = –184 (c = 0.69, MeOH).

R_f = 0.17 (CH_2Cl_2/MeOH = 20:1 (v/v); staining: CAM).

IR (ATR): 3194, 2967, 2876, 1616, 1589, 1552, 1474, 1429, 1371, 1319, 1276, 1211, 1187, 1157, 1130, 974, 951, 929, 844, 746, 603, 553, 508, 472, 431, 422, 406 cm^{-1}.

^{1}H NMR (300 MHz, MeOD-d_4): δ = 6.81–6.58 (m, 3H, H_{arom}), 4.29 (bs, 1H, H-9), 3.95–3.64 (m, 2H, H-8), 3.60–3.35 (m, 2H, H-12), 2.20–1.85 (m, 3H, H-10, H-11a), 1.85–1.64 (m, 1H, H-11b).

^{13}C NMR (75 MHz, MeOD-d_4): δ = 172.1 (C=O), 147.0 (C-6), 133.7 (C-7), 124.2 (C_q), 119.5 (C_{arom}), 119.3 (C_{arom}), 116.4 (C_{arom}), 63.4 (C-8), 60.4 (C-9), 51.4 (C-12), 28.6 (C-10), 25.6 (C-11).

HR-MS (ESI): *m/z* $[M+H]^+$ calcd for $C_{12}H_{17}N_2O_3^+$: 237.1234; found: 237.1224.

***cis/trans*-Tilimycin (2)**

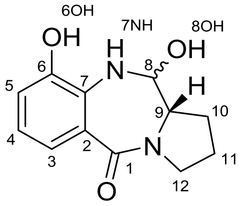

2

A dry 15 mL Schlenk tube with magnetic stirring bar was charged with a solution of 66.0 mg (279 µmol, 1.0 eq) (*S*)-(2-amino-3-hydroxyphenyl)(2-(hydroxymethyl)pyrrolidin-1-yl)methanone in 1.0 mL of a 1:1 mixture (v/v) of CH_2Cl_2/DMSO. After addition of 122 µL (698 µmol, 2.5 eq) DIPEA, the brown reaction mixture was cooled to 0 °C (ice bath) and 111 mg (698 µmol, 2.5 eq) SO_3·pyridine were added in one portion. The reaction mixture was stirred at 0 °C for 60 min until HPLC indicated full conversion. After warming to RT, CH_2Cl_2 was removed under reduced pressure at 23 °C (Schlenk line with preceding cooling trap). The crude orange residue was diluted with 200 µL DMSO and the unstable product was directly isolated via preparative RP-HPLC on the same day (Method A). Further purification of the resulting yellow solid (13.0 mg) via flash column chromatography (2 g silica gel, size: 130 x 6 mm, CH_2Cl_2/MeOH = 20:1 (v/v)) yielded the pure title compound as an off-white solid. Synthetic tilimycin (**2**) was obtained as an inseparable mixture of the *cis*- and *trans*-isomer (~1:1) and the NMR spectra were found to be identical to the natural *cis/trans*-tilimycin (**2**) (See Figure S2).

Yield: 10.3 mg (44.0 µmol, 16 %), off-white solid.

$C_{12}H_{14}N_2O_3$ [234.26 g/mol].

mp = 80–84 °C (decomp).

$[\alpha]_D^{23}$ = +300 (c = 0.48, MeOH).

R_f = 0.31 (CH_2Cl_2/MeOH = 20:1 (v/v); staining: CAM).

IR (ATR): 3086, 2927, 2875, 1602, 1559, 1509, 1432, 1368, 1257, 1195, 1107, 1065, 1003, 944, 893, 870, 823, 801, 744, 647, 629, 611, 562, 526, 509, 492, 475, 459, 439, 420, 410 cm^{-1}.

HR-MS (ESI): *m/z* $[M+H]^+$ calcd for $C_{12}H_{15}N_2O_3^+$: 235.1077; found: m/z 235.1069 (Δ = -0.8 mmu)

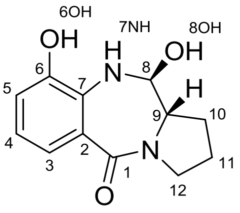

trans-Tilimycin (**2**)

Table S5: NMR data (^{1}H 700 MHz, ^{13}C 175 MHz) of the *trans*-isomer of synthetic tilimycin (**2**) in MeOD-d$_4$.

Atom #	δ_C, mult.	δ_H, mult. (Hz)
1	170.6, C_q	
2	130.2, C_q	
3	120.9, CH_{arom}	7.11, dd (7.7, 1.5)
4	123.1, CH_{arom}	6.87, t (7.8)
5	117.8, CH_{arom}	6.91, dd (7.9, 1.5)
6	150.2, C_q	
7	133.3, C_q	
8	96.8, CH	4.54, d (8.8)
9	60.6, CH	3.57-3.47, m
10	30.3, CH_2	2.20-1.97, m
11	23.8/23.9, CH_2	2.20-1.97, m
12	47.8, CH_2	3.74 – 3.66, m<br>3.57 – 3.47, m

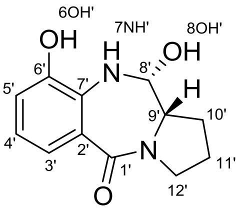

cis-Tilimycin (**2**)

Table S6: NMR data (^{1}H 700 MHz, ^{13}C 175 MHz) of the *cis*-isomer of synthetic tilimycin (**2**) in MeOD-d$_4$.

Atom #	δ_C, mult.	δ_H, mult. (Hz)
1’	169.2, C_q	
2’	119.4, C_q	
3’	123.6, CH_{arom}	7.31, dd (8.2, 1.5)
4’	117.7, CH_{arom}	6.59-6.56, m
5’	116.2, CH_{arom}	6.81, dd (7.6, 1.5)
6’	146.0, C_q	
7’	134.7, C_q	
8’	89.9, CH	4.75, s
9’	60.6, CH	3.82-3.76, m
10’	31.6, CH_2	2.35-2.27, m<br>2.20-1.97, m
11’	23.8/23.9, CH_2	2.20-1.97, m<br>1.86-1.78, m
12’	50.2, CH_2	3.74-3.66, m

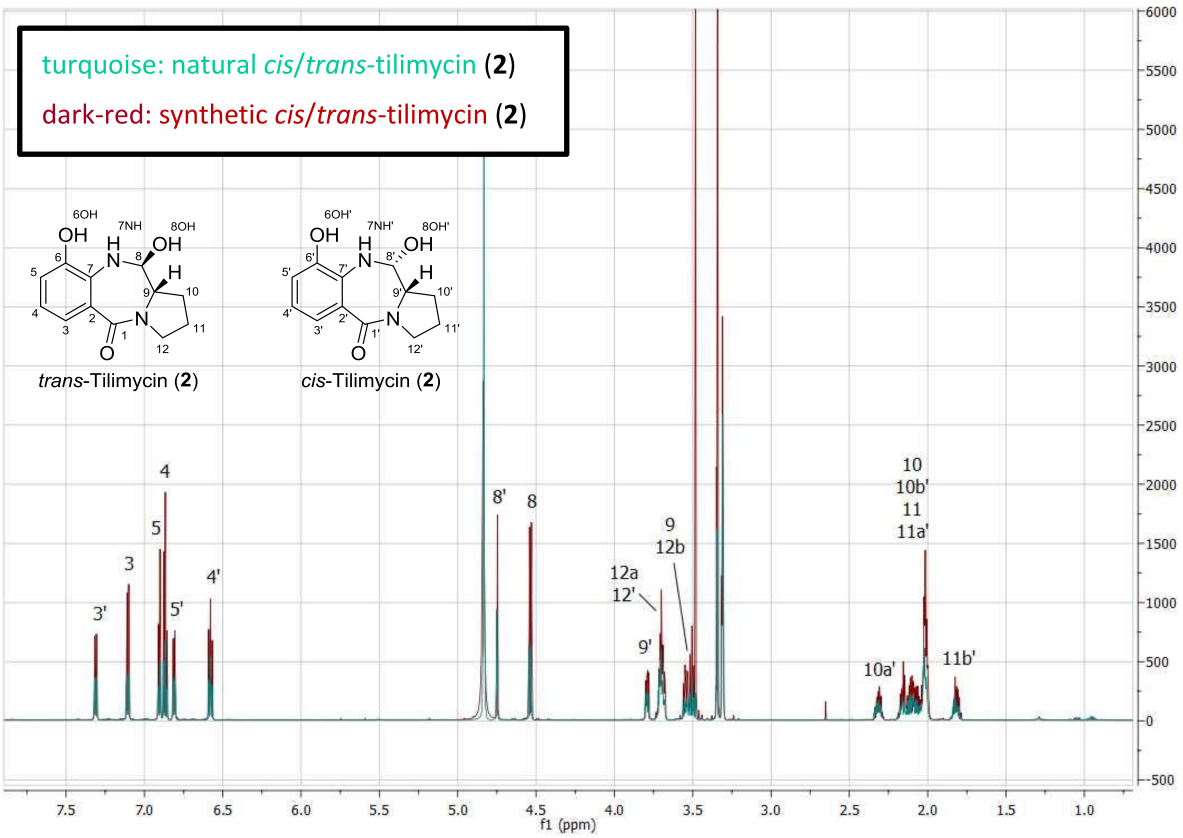

Figure S4: Superimposed proton NMR spectra (700 MHz, MeOH-d_4) of natural and synthetic *cis*/*trans*-tilimycin (**2**).

Isolation of culdesacin (3) from conditioned medium

K. oxytoca AHC-6 WT was cultivated in 100 mL CASO medium in a 300 mL Erlenmeyer flask (25x flasks were prepared in parallel) at 37 °C with orbital shaking at 180 rpm for 30 h. A total of 2.5 L of culture broth was centrifuged at 8000 rpm for 30 min at 4 °C. The supernatant was collected and filtrated through a 0.45 µm cellulose-acetate filter followed by filtration through a 0.2 µm cellulose-acetate filter to remove residual bacterial cells. Metabolites were extracted from the conditioned medium with 1-butanol (≥ 99.5 %, for synthesis, Roth) applying a 3:2 mixing ratio (two times). The organic extract was concentrated to dryness under high vacuum at 40 °C and was adsorbed on 2 g Celite™ (MeOH). The product was purified via two sequential flash column chromatographies (size: 36 x 1.8 cm, $CHCl_3$/MeOH = 20:1 (v/v), then 28 x 0.8 cm, $CHCl_3$/MeOH = 35:1 (v/v)) and further purified via preparative RP-HPLC (Method A). The solvent was removed on the rotary evaporator (bath temperature 40 °C) and the product was dried in high vacuum.

Yield: 5.9 mg (25.2 µmol), colorless, amorphous solid.

$C_{12}H_{14}N_2O_3$ [234.26 g/mol]

mp = 77-83 °C (decomp.).

$[\alpha]_D^{23}$ = +246 (c = 0.30, MeOH).

R_f = 0.11 (CH_3Cl/MeOH = 20:1 (v/v)) (254 nm, 366 nm, CAM: yellow).

IR (ATR): 3076, 2952, 2875, 1606, 1565, 1430, 1370, 1345, 1307, 1258, 1198, 1134, 1109, 1072, 1003, 921, 895, 876, 855, 821, 804, 745, 629, 608, 561, 494, 467, 420, 407 cm^{-1}.

HR-MS (ESI): *m/z* $[M+H]^+$ calcd for $C_{12}H_{15}N_2O_3^+$: 235.1077; found: m/z 235.1069 (Δ = -0.8 mmu)

NMR-data of culdesacin (3)

Culdesacin (**3**) was obtained as a single isomer and the signals were assigned to the atoms by 1D and 2D NMR experiments. The configuration of the hydroxyl group 8OH was determined by NOESY experiments (see SI-7).

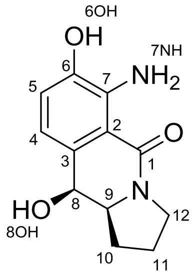

Culdesacin (**3**)

Table S7: NMR data (^{1}H 500 MHz, ^{13}C 126 MHz) of natural culdesacin (**3**) in DMSO-d$_6$.

Atom #	δ_C, mult.	δ_H, mult. (Hz)	HMBC
1	164.7, C_q		
2	109.6, C_q		8, 4
3	132.4, C_q		8, 5
4	114.3, CH_{arom}	6.34, d (7.6)	8
5	115.0, CH_{arom}	6.70, d (7.7)	
6	144.3, C_q		4, 5
6OH		9.41, bs	
7	139.1, C_q		5
7NH		6.23, bs	
8	66.1, CH	4.28, dd (5.3, 2.0)	4
8OH		4.96, bd (5.5)	
9	60.6, CH	3.65, t (7.6)	8OH
10	26.8, CH_2	2.15 – 2.04, m<br>2.00 – 1.88, m	
11	22.7, CH_2	2.00 – 1.88, m<br>1.82 – 1.70, m	
12	44.8, CH_2	3.53, t (9.8)<br>3.38-3.28, m	10/11

SI-4. Total Synthesis of tilivalline (1)

Tilivalline (**1**) used for this study was synthesized according to the following procedure. The transformations from **11** to **1** were adapted from a previous total synthesis by Nagasaka.[8]

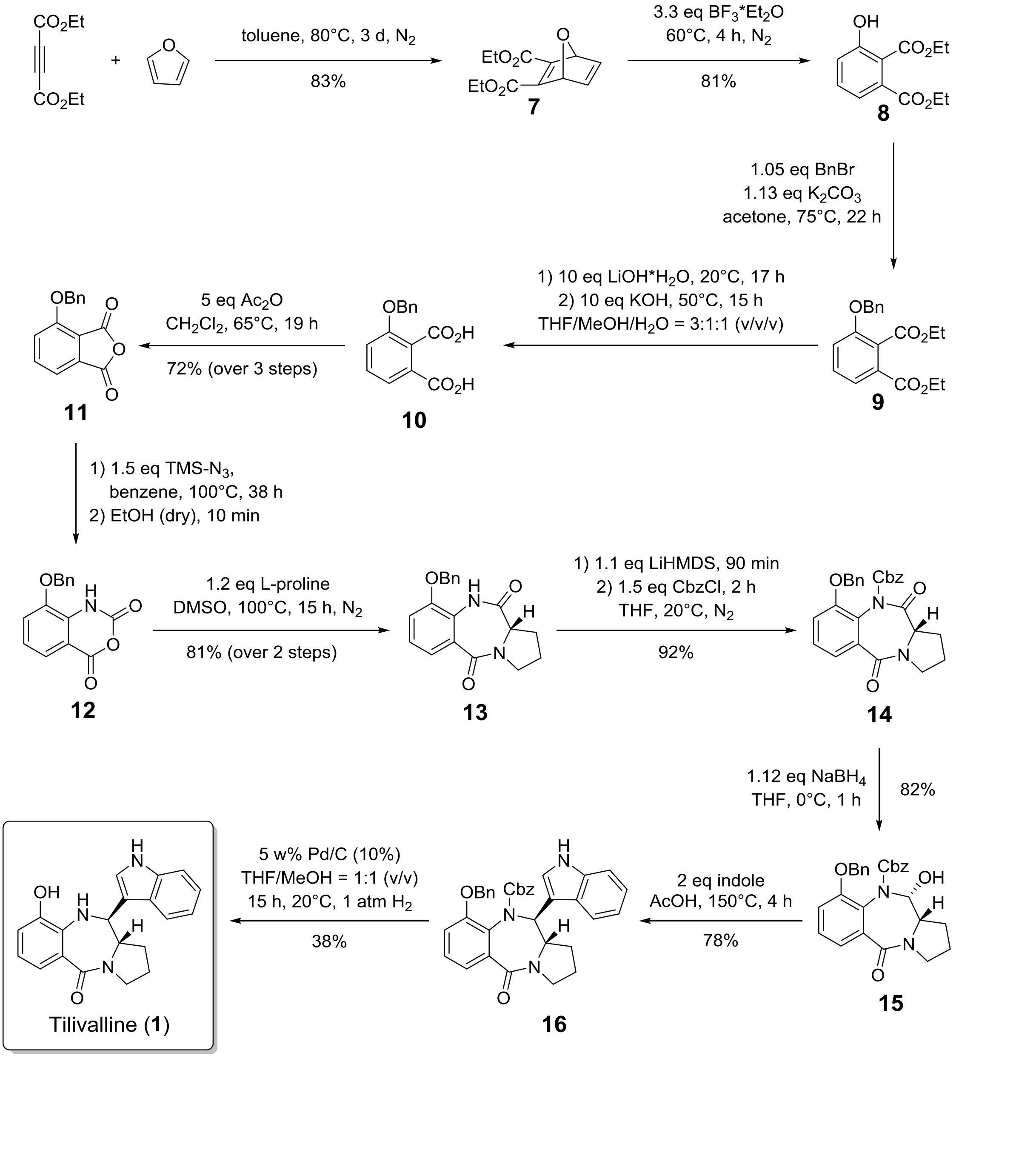

Diethyl 7-oxabicyclo[2.2.1]hepta-2,5-diene-2,3-dicarboxylate (7)

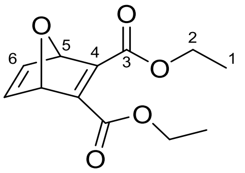

7

A dry 150 mL Schlenk flask with magnetic stirring bar was charged with 50 mL dry toluene, 14.1 mL (15.0 g, 83.7 mmol, 1.0 eq) diethyl acetylenedicarboxylate and 7.3 mL (6.84 g, 100 mmol, 1.2 eq) furan in an N_2 counter-stream. The light-yellow reaction mixture was heated to 80 °C for 3 d until full conversion of the starting material was observed by GC-MS. The solvent was removed in vacuum and the crude product was purified via column chromatography (300 g silica gel, size: 20 x 6 cm, cyclohexane/EtOAc = 3:1 (v/v), 200 mL fractions).[9]

Yield: 16.5 g (69.4 mmol, 83%) light-yellow liquid.

R_f = 0.23 (cyclohexane/EtOAc = 1:1 (v/v) (CAM: dark-blue).

^{1}H-NMR (300.36 MHz, $CDCl_3$): δ = 7.22 (s, 2H, H-6), 5.67 (s, 2H, H-5), 4.27 (q, $^3J_{HH}$ = 7.1 Hz, 4H, H-2), 1.32 (t, $^3J_{HH}$ = 7.1 Hz, 6H).

^{13}C-NMR (75.53 MHz, $CDCl_3$): δ = 163.2 (C-3), 152.8 (C-4), 143.3 (C-6), 85.2 (C-5), 61.5 (C-2), 14.2 (C-6).

The spectra are in accordance with previously reported data.[10]

Diethyl 3-hydroxyphthalate (8)

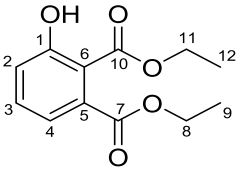

8

A dry 100 mL round-bottom flask with magnetic stirring bar, reflux condenser and pressure equilibrator was charged with 16.5 g (69.4 mmol, 1.0 eq) diethyl 7-oxabicyclo[2.2.1]hepta-2,5-diene-2,3-dicarboxylate (**7**) and 30 mL (32.8 g, 232 mmol, 3.34 eq) $BF_3 \cdot Et_2O$ in an N_2 counter-stream. The dark-brown solution was heated to 60 °C for 4 h until full conversion of

the starting material was detected by ^{1}H-NMR. The reaction mixture was poured on 200 mL cold saturated $NaHCO_3$ solution and solid Na_2CO_3 was added until neutral. The dark-brown suspension was extracted with Et_2O (3 x 100 mL, 1 x 150 mL). The combined organic phases were dried over Na_2SO_4, filtrated and the solvent was removed in vacuum. The brown liquid was dried in oil pump vacuum and was used in the following steps without further purification.[11]

Yield: 13.4 g (56.1 mmol, 81%).

R_f = 0.48 (cyclohexane/EtOAc = 3:1 (v/v)) (254 nm, CAM: brown).

^{1}H-NMR (300.36 MHz, $CDCl_3$): δ = 7.44 (t, $^3J_{HH}$ = 7.9 Hz, 1H, H-3), 7.07 (dd, $^3J_{HH}$ = 8.4 Hz, $^4J_{HH}$ = 1.0 Hz, 1H, H_{arom}), 6.94 (dd, $^3J_{HH}$ = 7.4 Hz, $^4J_{HH}$ = 1.0 Hz, 1H, H_{arom}), 4.45-4.28 (m, 4H, H-8, H-11), 1.37 (t, $^3J_{HH}$ = 7.1 Hz, 6H, H-9, H-12).

^{13}C-NMR (75.53 MHz, $CDCl_3$): δ = 169.2 (C_q), 169.0 (C_q), 136.0 (C_q), 134.6 (C-3), 119.7 (CH_{arom}), 119.1 (CH_{arom}), 62.3 (CH_2), 61.7 (CH_2), 14.2 (CH_3), 14.0 (CH_3).

4-(Benzyloxy)isobenzofuran-1,3-dione (11)

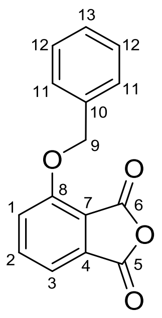

11

<u>Step 1</u>: A 250 mL round-bottom flask with magnetic stirring bar was charged with 13.3 g (55.9 mmol, 1.0 eq) diethyl 3-hydroxyphthalate (**8**), 8.82 g (63.2 mmol, 1.13 eq) K_2CO_3 and 100 mL distilled acetone. To the stirred suspension were added 7.16 mL (10.3 g, 58.7 mmol, 1.05 eq) benzyl bromide and the brown suspension was heated to 75 °C for 22 h until full conversion of the starting material was detected (TLC). The mixture was cooled to 22 °C and the solvent was removed in vacuum. The brown residue was dissolved in 150 mL THF/MeOH/H_2O (3:1:1 (v/v/v)) and was treated with 41.9 g (988 mmol, 17 eq) LiOH·H_2O. The mixture was stirred at 22 °C for 17 h (^{1}H-NMR: full mono-saponification) and was treated with 34.9 g (559 mmol, 10 eq) KOH. The mixture was heated to 50 °C for 15 h until complete

saponification was detected (^{1}H-NMR). The red-brown suspension was cooled to 0 °C and acidified with 12 M HCl (130 mL). The mixture was extracted with EtOAc (3 x 200 mL), the combined organic layers were dried over Na_2SO_4, filtrated and the solvent was removed in vacuum. The brown solid was used in the following step without further purification.

Step 2: A 200 mL Schlenk flask with magnetic stirring bar was charged with the crude 3-(benzyloxy)phthalic acid **10**, 26.5 mL (28.8 g, 279 mmol, 5 eq) Ac_2O and 75 mL CH_2Cl_2. The brown suspension was heated to 65 °C for 19 h and the brown solution was cooled to 22 °C and was left standing for 2 h to crystallize. The product was isolated via filtration through a sintered glass frit, washed with *n*-pentane (3 x 40 mL) and dried in oil pump vacuum (first fraction, 8.48 g, 60%). The filtrate was concentrated in vacuum and the brown/black solid was dissolved in 50 mL EtOAc. The mixture was treated with 40 mL *n*-pentane and the cloudy precipitate was collected via filtration through a sintered glass frit. The beige crystals (second fraction) were washed with *n*-pentane (2 x 20 mL) and were dried in oil pump vacuum (second fraction, 1.68 g, 12%).

Yield: 10.2 g (40.0 mmol, 72%) light-yellow crystals.

R_f = 0.36 (cyclohexane/EtOAc = 2:1 (v/v)) (UV: 254 nm, 366 nm, CAM: dark blue).

mp: 140 °C.

^{1}H-NMR (300.36 MHz, $CDCl_3$): δ = 7.76 (t, $^3J_{HH}$ = 7.9 Hz, 1H, H-2), 7.56 (d, $^3J_{HH}$ = 7.3 Hz, 1H, H_{arom}), 7.51-7.44 (m, 2H, H-11), 7.43-7.28 (m, 4H, H_{arom}), 5.38 (s, 2H, H-9).

^{13}C-NMR (75.53 MHz, $CDCl_3$): δ = 163.0 (C_q), 160.6 (C_q), 157.4 (C-8, C_q), 138.3 (C-2, C_q), 135.1 (C_q), 133.5 (C_q), 129.0 (C-12), 128.6 (C-13), 127.0 (C-11), 120.5 (C_q), 117.8 (C_q), 71.3 (C-9).

The spectra are in accordance with previously reported data.[12]

8-(Benzyloxy)-2*H*-benzo[*d*][1,3]oxazine-2,4(1*H*)-dione (12)

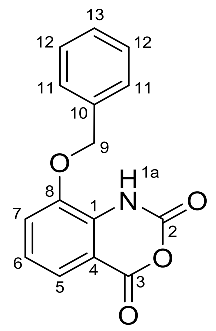

12

A dry 50 mL Schlenk flask with magnetic stirring bar was charged with 854 mg (3.36 mmol, 1.0 eq) 4-(benzyloxy)isobenzofuran-1,3-dione (**11**) and the solid was dried in oil pump vacuum for 10 min. 13 mL dry benzene were added under N_2 counter-stream followed by 740 µL (618 mg, 5.04 mmol, 1.5 eq) TMS-N_3.[Safety Note: Azides are potentially explosive reagent, which have to be handled with care. The use of explosion protection shields is strongly advised! The scale up of the reaction should be avoided, but parallel reactions should be performed instead.] The yellow suspension was heated to 100 °C for 16 h. The dirty-yellow solution was cooled to 22 °C and most of the solvent was removed using a cooling trap (~1 mL yellow viscous liquid left). The residue was heated to 100 °C for 22 h, cooled to 22 °C and 6 mL dry EtOH were added. The light brown suspension was stirred for 5 min, the solvent was removed with a cooling trap and the product was dried in oil pump vacuum to yield a light brown solid. The crude product (891 mg, brown solid) was used in the following step without further purification. The yield of the by-product 5-(benzyloxy)-2*H*-benzo[*d*][1,3]oxazine-2,4(1*H*)-dione was determined as <1% (^{1}H-NMR) and typically ranges from 1-28% in analogous experiments. The by-product can be removed by recrystallization from EtOAc.[8]

R_f = 0.53 (cyclohexane/EtOAc = 1:1 (v/v)) (UV: 254 nm, CAM: blue).

mp: 194-195 °C.

^{1}H-NMR (300.36 MHz, $CDCl_3$): δ = 8.10 (bs, 1H, H-1a), 7.67 (d, $^3J_{HH}$ = 7.8 Hz, 1H, H_{arom}), 7.50-7.34 (m, 5H, H_{arom}), 7.23-7.12 (m, 2H, H_{arom}), 5.18 (s, 2H, H-9).

^{13}C-NMR (75.53 MHz, $CDCl_3$): δ = 159.8 (C-3, C_q), 146.9 (C-8, C_q), 145.0 (C-2, C_q), 136.1 (C-10, C_q), 131.9 (C-1, C_q), 128.3 (C_{arom}), 128.0 (C_{arom}), 127.9 (C_{arom}), 123.3 (C_{arom}), 120.0 (C_{arom}), 119.0 (C_{arom}), 111.1 (C-4, C_q), 70.1 (C-9).

The spectra are in accordance with previously reported data.[8]

(*S*)-9-(Benzyloxy)-1,2,3,11a-tetrahydro-5*H*-benzo[*e*]pyrrolo[1,2-*a*][1,4]diazepine-5,11(10*H*)-dione (13)

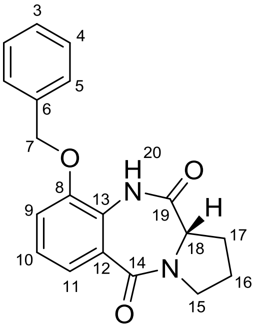

13

A dry 20 mL Schlenk flask with magnetic stirring bar was charged with 1.30 g (4.81 mmol, 1.0 eq) crude 8-(benzyloxy)-2*H*-benzo[*d*][1,3]oxazine-2,4(1*H*)-dione (**12**) from the previous step, 672 mg (5.78 mmol, 1.2 eq) L-proline and 6 mL dry DMSO in a N_2 counter-stream. The beige suspension was heated to 100 °C for 15 h until full conversion of the starting material was detected by TLC. The dark-yellow solution was poured into distilled H_2O (30 mL) and the colorless suspension was stirred for 2 h at 22 °C. The light-yellow solid was isolated via filtration, washed with H_2O (2 x 10 mL) and dried in oil pump vacuum. The solid was dissolved in boiling toluene (50 mL) and was filtrated through a (heated) sintered glass frit. The solvent was removed in vacuum and the resulting solid was dried in oil pump vacuum.[8]

Yield: 1.25 g (3.89 mmol, 81%) colorless, amorphous solid.

R_f = 0.42 (cyclohexane/CH_2Cl_2/acetone = 2:2:1 (v/v/v)) (UV: 254 nm, CAM: blue).

mp: 177-178 °C.

^{1}H-NMR (300.36 MHz, $CDCl_3$): δ = 7.98 (s, 1H, H-20), 7.58 (d, $^3J_{HH}$ = 7.5 Hz, 1H, H-11), 7.47-7.30 (m, 5H, H_{arom}), 7.17 (t, $^3J_{HH}$ = 7.8 Hz, 1H, H-10), 7.09 (d, $^3J_{HH}$ = 7.6 Hz, 1H, H-9), 5.12 (s, 2H, H-7), 4.02 (d, $^3J_{HH}$ = 5.1 Hz, 1H, H-18), 3.89-3.71 (m, 1H, H-15a), 3.68-3.50 (m, 1H, H-15b), 2.86-2.65 (m, 1H, H-17a), 2.16-1.89 (m, 3H, H-16, H-17b).

^{13}C-NMR (75.53 MHz, $CDCl_3$): δ = 170.5 (C-19, C_q), 165.2 (C-14, C_q), 147.8 (C-8, C_q), 135.6 (C-6, C_q), 129.0 (C_{arom}), 128.7 (C-3), 127.9 (C_{arom}), 127.6 (C-13, C_q), 125.7 (C-12, C_q), 124.8 (C-10), 122.9 (C-11), 114.6 (C-9), 71.5 (C-7), 57.0 (C-18), 47.5 (C-15), 26.4 (C-16), 23.5 (C-17).

The spectra are in accordance with previously reported data.[8]

Benzyl (*S*)-9-(benzyloxy)-5,11-dioxo-2,3,11,11a-tetrahydro-1*H*-benzo[*e*]pyrrolo[1,2-*a*][1,4]diazepine-10(5*H*)-carboxylate (**14**)

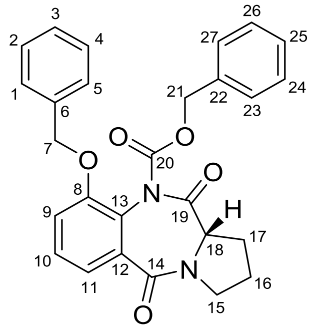

14

A dry 200 mL Schlenk flask with magnetic stirring bar was charged with 1.20 g (3.72 mmol, 1.0 eq) (*S*)-9-(benzyloxy)-1,2,3,11a-tetrahydro-5*H*-benzo[*e*]pyrrolo[1,2-*a*][1,4]diazepine-5,11(10*H*)-dione (**13**) and 72 mL dry THF in a N_2 counter-stream. To the colorless solution were added 4.1 mL (4.09 mmol, 1.1 eq) LiHMDS solution (1.0 M in hexanes) over a period of 5 min at 22 °C. After 90 min a thick colorless suspension has formed and 821 µL (982 mg, 5.58 mmol, 1.5 eq) CbzCl were added in one portion. The light-yellow solution was stirred at 22 °C for 150 min until full conversion of the starting material was detected (TLC). 2 mL saturated NH_4Cl solution were added and the mixture was stirred for 5 min. The mixture was diluted with CH_2Cl_2 (50 mL) and the solid was removed via filtration through a sintered glass frit and was rinsed with CH_2Cl_2. The combined organic phases were dried over Na_2SO_4, filtrated and the solvent was removed in vacuum. The product was purified via column chromatography (60 g silica gel, size: 20 x 3 cm, cyclohexane/CH_2Cl_2/acetone = 10:10:1 (v/v/v), 20 mL fractions).[8]

Yield: 1.56 g (3.42 mmol, 92%) faint-yellow foam.

R_f = 0.52 (cyclohexane/CH_2Cl_2/acetone = 2:2:1 (v/v/v)) (UV: 254 nm, CAM: blue).

mp: 50-52 °C.

^{1}H-NMR (300.36 MHz, $CDCl_3$): δ = 7.49 (dd, $^3J_{HH}$ = 7.8 Hz, $^4J_{HH}$ = 1.0 Hz, 1H, H_{arom}), 7.40-7.31 (m, 2H, H_{arom}), 7.30-7.25 (m, 2H, H_{arom}), 7.24-7.15 (m, 5H, H_{arom}), 7.14-7.03 (m, 3H, H_{arom}), 5.10 (d, $^3J_{HH}$ = 8.0 Hz, 1H, H-7a), 4.90-4.88 (m, 2H, H-7b, H21a), 4.78 (d, $^3J_{HH}$ = 12.0 Hz, 1H, H-21b), 3.92 (d, $^3J_{HH}$ = 6.7 Hz, 1H, H-18), 3.86-3.71 (m, 1H, H-15a), 3.58-3.41 (m, 1H, H-15b), 2.73-2.59 (m, 1H, H-17a), 2.20-1.83 (m, 3H, H-16, H-17b).

^{13}C-NMR (75.53 MHz, $CDCl_3$): δ = 169.0 (C-19, C_q), 164.7 (C-14, C_q), 152.4 (C_q), 151.8 (C_q), 135.7 (C_q), 134.8 (C_q), 133.1 (C_q), 129.0, 128.7, 128.5, 128.3, 128.2, 128.1, 127.3, 125.8 (C_q), 121.9 (C-11), 116.2 (C-9), 71.6 (C-21), 68.8 (C-7), 59.3 (C-18), 46.7 (C-15), 26.5 (C-17), 23.7 (C-16).

The spectra are in accordance with previously reported data.[8]

Benzyl (11*R*,11a*S*)-9-(benzyloxy)-11-hydroxy-5-oxo-2,3,11,11a-tetrahydro-1*H*-benzo[*e*]pyrrolo[1,2-*a*][1,4]diazepine-10(5*H*)-carboxylate (15)

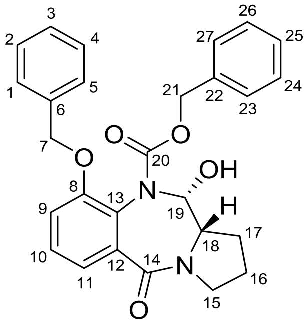

15

A dry 100 mL Schlenk flask with magnetic stirring bar was charged with 390 mg (854 µmol, 1.0 eq) benzyl (*S*)-9-(benzyloxy)-5,11-dioxo-2,3,11,11a-tetrahydro-1*H*-benzo[*e*]pyrrolo[1,2-*a*][1,4]diazepine-10(5*H*)-carboxylate (**14**) and 14 mL dry THF in a N_2 counter-stream. The colorless solution was cooled to 0 °C and 34.6 mg (957 µmol, 1.12 eq) $NaBH_4$ were added in one portion. The mixture was stirred at 0 °C until full conversion of the starting material was detected after 1 h (TLC). The solvent was carefully removed in vacuum (max. 35 °C) and the polar by-products were removed via silica gel filtration (3 g silica gel, 100 mL cyclohexane/CH_2Cl_2/acetone = 2:2:1). The solvent was removed in vacuum and the product was dried in oil pump vacuum to obtain pure product.[8]

Yield: 321 mg (700 µmol, 82%) colorless, amorphous solid.

R_f = 0.32 (cyclohexane/CH_2Cl_2/acetone = 2:2:1 (v/v/v)) (UV: 254 nm, CAM: blue).

mp: 170-174 °C.

^{1}H-NMR (300.36 MHz, $CDCl_3$): δ = 7.41-7.16 (m, 11H, H_{arom}), 7.09-6.98 (m, 2H, H_{arom}), 5.63 (d, $^3J_{HH}$ = 9.7 Hz, 1H, H-19), 5.09 (d, $^3J_{HH}$ = 11.2 Hz, 1H, H-7a), 5.04-4.89 (m, 2H, H- 7b, H-21a), 4.79 (d, $^3J_{HH}$ = 12.0 Hz, 1H, H-21b), 3.82-3.69 (m, 1H, H-15a), 3.62-3.39 (m, 2H, H-15b, H-18), 2.20-2.10 (m, 2H, H-17), 2.08-1.94 (m, 2H, H-16).

^{13}C-NMR (75.53 MHz, $CDCl_3$): δ = 166.9 (C_q, C-14), 156.8 (C_q), 154.6 (C_q), 136.5 (C_q), 135.9 (C_q), 135.2 (C_q), 128.9, 128.7, 128.4, 128.0, 127.9, 127.6, 126.7, 124.9 (C_q), 120.9 (C-11), 115.2 (C-9), 86.3 (C-19), 70.6/67.9 ($CH_{2,\ benzyl}$), 59.9 (C-18), 46.4 (C-15), 28.8 (C-17), 23.2 (C-16).

The spectra are in accordance with previously reported data.[8]

Benzyl (11*S*,11a*S*)-9-(benzyloxy)-11-(1*H*-indol-3-yl)-5-oxo-2,3,11,11a-tetrahydro-1*H*-benzo[*e*]pyrrolo[1,2-*a*][1,4]diazepine-10(5*H*)-carboxylate (16)

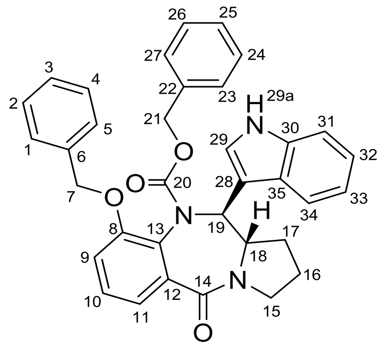

16

A dry 4 mL pressure tube with magnetic stirring bar was charged with 49.9 mg (109 µmol, 1.0 eq) benzyl (11*R*,11a*S*)-9-(benzyloxy)-11-hydroxy-5-oxo-2,3,11,11a-tetrahydro-1*H*-benzo[*e*]pyrrolo[1,2-*a*][1,4]diazepine-10(5*H*)-carboxylate (**15**), 25.5 mg (218 µmol, 2.0 eq) indole and 1.5 mL AcOH. The light-yellow solution was degassed by bubbling N_2 through the solution in an ultrasonic bath (10 min). The pressure tube was sealed and placed into a preheated oil bath (150 °C) and was stirred for 4 h until full conversion of the starting material was observed (TLC). The mixture was cooled to 22 °C, the light-brown solution was concentrated in vacuum and the redish-brown residue was purified via column chromatography (4 g silica gel, cyclohexane/CH_2Cl_2/acetone = 2:2:1, 4 mL fractions).[8]

Yield: 47.5 mg (85.2 µmol, 78%) light-red solid.

R_f = 0.38 (cyclohexane/CH_2Cl_2/acetone = 2:2:1) (UV: 254 nm, CAM: blue).

mp: 250-254 °C.

^{1}H-NMR (300.36 MHz, $CDCl_3$): δ = 8.23 (s, 1H, H-29a), 7.51-6.86 (m, 18H, H_{arom}), 6.34 (d, $^3J_{HH}$ = 7.3 Hz, 1H), 5.69 (d, , $^3J_{HH}$ = 11.6 Hz, 1H, H-19), 4.91 (AB_q, $^2J_{HH}$ = 12.9 Hz, 2H, H-7), 4.44 (d, $^2J_{HH}$ = 11.9 Hz, 1H, H-21a), 4.36-4.22 (m, 2H, H-18, H-21b), 3.95-3.80 (m, 1H, H-15a), 3.76-3.56 (m, 1H, H-15b), 2.24-2.06 (m, 1H, H-16a), 2.06-1.88 (m, 2H, H-16b, H-17a), 1.84-1.73 (m, 1H, H-17b).

^{13}C-NMR (75.53 MHz, $CDCl_3$): δ = 167.6 (C-14), 155.6 (C_q), 155.4 (C_q), 136.6 (C_q), 136.5 (C_q), 136.4 (C_q), 136.2 (C_q), 129.2 (C_{arom}), 128.2 (C_{arom}), 128.1 (C_{arom}), 127.5 (C_{arom}), 127.2 (C_{arom}), 127.1 (C_{arom}), 126.5 (C_q), 126.0 (C_{arom}), 123.8 (C_{arom}), 122.3 (C_{arom}), 120.7 (C_{arom}), 120.6 (C_{arom}), 119.7 (C_{arom}), 114.9 (C_{arom}), 113.8 (C_q), 111.3 (C_{arom}), 69.7 (C-21), 67.2 (C-7), 63.0 (C-19), 59.7 (C-18), 46.7 (C-15), 29.5 (C-17), 23.3 (C-16).

The spectra are in accordance with previously reported data.[8]

Tilivalline (1)

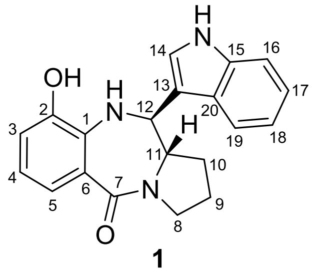

1

A dry 100 mL Schlenk flask with magnetic stirring bar was charged with 150 mg (269 µmol) benzyl (11*S*,11a*S*)-9-(benzyloxy)-11-(1*H*-indol-3-yl)-5-oxo-2,3,11,11a-tetrahydro-1*H*-benzo[*e*]pyrrolo[1,2-*a*][1,4]diazepine-10(5*H*)-carboxylate (**16**), 4 mL dry THF, 4 mL dry MeOH and 7.5 mg Pd/C (10% on activated charcoal) in a N_2 counter-stream. The flask was degassed by repeated vacuum/N_2-cycles (3 x) and was filled with H_2 (1 atm) the last time. The suspension was stirred at 22 °C for 15 h until full conversion of the starting material was detected by TLC. The catalyst was removed via filtration through a pad of Celite™ (2 cm, wetted with MeOH) and the pad was rinsed with MeOH (3 x 25 mL). The solvent was removed in vacuum and the product was purified via preparative RP-HPLC. [4],[8]

Yield: 33.8 mg (101 µmol, 38%) light-carnate solid.

R_f = 0.09 (cyclohexane/CH_2Cl_2/acetone = 2:2:1) (UV: 255/366 nm, CAM: orange).

mp = 179-182 °C.

^{1}H-NMR (499.89 MHz, DMSO-d_6): δ = 11.10 (bs, 1H, NH), 7.44-7.36 (m, 3H, H-14, H-16, H-19), 7.16 (d, $^3J_{HH}$ = 7.4 Hz, 1H, H-5), 7.09 (t, $^3J_{HH}$ = 7.3 Hz, 1H, H-17), 6.94 (t, $^3J_{HH}$ = 7.3 Hz, 1H, H-18), 6.77 (d, $^3J_{HH}$ = 7.1 Hz, 1H, H-3), 6.53 (t, $^3J_{HH}$ = 7.8 Hz, 1H, H-4), 5.09 (bs, 1H, OH), 4.73 (d, $^3J_{HH}$ = 9.1 Hz, 1H, H-12), 4.21-4.13 (m, 1H, H-11), 3.73-3.66 (m, 1H, H-8a), 3.60-3.51 (m, 1H, H-8b), 1.94-1.84 (m, 1H, H-9a), 1.75-1.65 (m, 2H, H-9b, H-10a), 1.58-1.49 (m, 1H, H-10b).

^{13}C-NMR (125.70 MHz, DMSO-d_6): δ = 166.4 (C-7, C_q), 145.2 (C-2, C_q), 136.5 (C-15, C_q), 134.7 (C-1, C_q), 125.1 (C-20, C_q), 123.7 (C-14, C_{arom}),121.6 (C-17, C_{arom}), 121.3 (C-5, C_{arom}), 120.1 (C-18, C_{arom}), 118.90 (C-19, C_{arom}), 118.87 (C-13, C_q), 116.4 (C-4, C_{arom}), 116.2 (C-6, C_q), 114.9 (C-3, C_{arom}), 111.8 (C-16, C_{arom}), 60.9 (CH, C-12), 59.5 (CH, C-11), 47.5 (CH_2, C-8), 30.2 (CH_2, C-10), 22.0 (CH_2, C-9).

$[\alpha]_D^{20}$= +178 (c = 0.29 in MeOH).

The spectra are in accordance with previously reported data.[8]

SI-5. Total Synthesis of *cis/trans*-deoxy-tilimycin (4)

(*S*)-(2-Aminophenyl)(2-(hydroxymethyl)pyrrolidin-1-yl)methanone

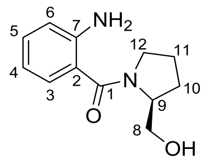

A dry 150 mL Schlenk flask with magnetic stirring bar was charged with 2.50 g (15.3 mmol, 1 eq) isatoic anhydride and 25 mL DMSO abs. in an Ar counter-stream. After addition of 1.66 mL (16.9 mmol, 1.1 eq) L-prolinol, the brown solution was stirred for 17 h at 100 °C. The reaction mixture was cooled to RT, transferred into a 250 mL separation funnel, and diluted with 100 mL H_2O. The product was extracted with EtOAc (3 x 50 mL). The combined organic layers were washed with H_2O (2 x 25 mL) and brine (25 mL), dried over Na_2SO_4 and filtered. The solvent was removed under reduced pressure and the crude product dried in oil-pump vacuum. Purification of the brown viscous mass (1.19 g) via flash column chromatography

(120 g silica gel, size: 160 x 50 mm, cyclohexane/CH_2Cl_2/acetone = 2:2:1 (v/v/v)) provided the title compound as a brown oil.

Yield: 820 mg (3.72 mmol, 24 %), brown oil.

$C_{12}H_{16}N_2O_2$ [220.27 g/mol].

R_f = 0.26 (cyclohexane/CH_2Cl_2/acetone = 1:1:1 (v/v/v); staining: CAM).

IR (ATR): 3348, 2967, 2875, 1610, 1581, 1493, 1451, 1418, 1370, 1310, 1266, 1245, 1209, 1157, 1085, 1044, 970, 944, 897, 749, 728, 655, 589, 543, 523, 469, 437, 403 cm^{-1}.

^{1}H NMR (300 MHz, MeOD-d_4, mixture of two rotamers): δ = 7.25–7.08 (m, 2H, H_{arom}), 6.79 (d, $^3J_{HH}$ = 7.8 Hz, 1H, H_{arom}), 6.70 (t, $^3J_{HH}$ = 7.1 Hz, 1H, H_{arom}), 4.40-4.16 (m, 1H, H-9), 3.95–3.62 (m, 2H, H-8), 3.60–3.37 (m, 2H, H-12), 2.15–1.89 (m, 3H, H-10, H-11a), 1.88–1.70 (m, 1H, H-11b).

^{13}C NMR (75 MHz, MeOD-d_4, mixture of two rotamers, major rotamer): δ = 172.3 (C=O), 146.4 (C-7), 131.8 (C_{arom}), 128.7 (C_{arom}), 122.8 (C_{arom}), 118.1 (C_{arom}), 117.5 (C_{arom}), 63.3 (C-8), 60.4 (C-9), 51.3 (C-12), 28.6 (C-10), 25.6 (C-11).

HR-MS (ESI): *m/z* $[M+H]^+$ calcd for $C_{12}H_{17}N_2O_2^+$: 221.1285; found: 221.1276.

***cis/trans*-Deoxy-tilimycin (4)**

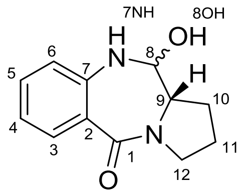

Deoxy-tilimycin (**4**)

A dry 15 mL Schlenk tube with magnetic stirring bar was charged with a solution of 125 mg (567 µmol, 1 eq) (*S*)-(2-aminophenyl)(2-(hydroxymethyl)pyrrolidin-1-yl)methanone in 2.0 mL of a 1:1 mixture (v/v) of CH_2Cl_2/DMSO. After addition of 248 µL (1.42 mmol, 2.5 eq) DIPEA, the brown reaction mixture was cooled to 0 °C (ice bath) and 226 mg (1.42 mmol, 2.5 eq) SO_3·pyridine were added in one portion. The reaction mixture was stirred at 0 °C for 60 min until HPLC indicated full conversion. The ice bath was removed and CH_2Cl_2 was removed under reduced pressure at 23 °C (Schlenk line with preceding cooling trap). The product was directly isolated via preparative RP-HPLC on the same day (method A). Further purification of

the resulting colorless solid (18.5 mg) via flash column chromatography (2 g silica gel, size: 130 x 6 mm, CH_2Cl_2/MeOH = 20:1 (v/v)) yielded the title compound **4** as a colorless solid. Deoxy-tilimycin (**4**) was obtained as an inseparable mixture of the *cis*- and *trans*-isomer.[13]

Yield: 12.8 mg (58.6 µmol, 10%), colorless solid.

$C_{12}H_{14}N_2O_2$ [218.26 g/mol].mp = 60–65 °C.

$[\alpha]_D^{23}$ = +328 (c = 0.59, MeOH).

R_f = 0.34 (CH_2Cl_2/MeOH = 20:1 (v/v); staining: CAM).

IR (ATR): 3308, 3061, 2974, 2949, 2877, 2825, 2460, 2202, 2061, 1612, 1487, 1453, 1417, 1378, 1357, 1308, 1297, 1243, 1205, 1187, 1163, 1136, 1102, 1069, 982, 951, 924, 868, 830, 792, 751, 712, 700, 649, 608, 528, 465 cm^{-1}.

HR-MS (ESI): *m/z* $[M+H]^+$ calcd for $C_{12}H_{15}N_2O_2^+$: 219.1128; found: m/z 219.1121 (Δ = -0.7 mmu)

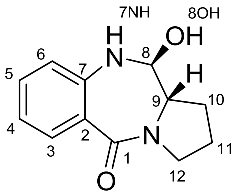

trans-Deoxy-tilimycin (**4**)

Table S8: NMR data (^{1}H 700 MHz, ^{13}C 175 MHz) of the *trans*-isomer of synthetic deoxy-tilimycin (**4**) in MeOD-d_4.

Atom #	δ_C, mult.	δ_H, mult. (Hz)
1	170.4, C_q	
2	129.8, C_q	
3	130.4, CH_{arom}	7.62, dt (7.8, 4.0)
4	123.1, CH_{arom}	7.05, dt (7.6, 1.0)
5	133.3/133.2/133.1, CH_{arom}	7.38-7.31, m
6	119.1/118.3, CH_{arom}	6.78-6.71, m
7	144.9, C_q	
8	96.7, CH	4.46, d (8.9)
9	60.5/60.3, CH	3.60-3.49, m
10	30.2, CH_2	2.23-1.97, m
11	23.84/23.82, CH_2	2.23-1.97, m<br>1.88-1.77, m
12	47.8, CH_2	3.75-3.65, m<br>3.60-3.49, m

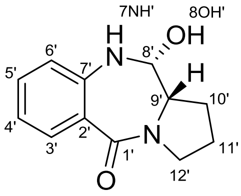

cis-Deoxy-tilimycin (**4**)

Table S9: NMR data (^{1}H 700 MHz, ^{13}C 175 MHz) of the *cis*-isomer of synthetic deoxy-tilimycin (**4**) in MeOD-d_4.

Atom #	δ_C, mult.	δ_H, mult. (Hz)
1’	169.0, C_q	
2’	119.0, C_q	
3’	133.3/133.2/133.1, CH_{arom}	7.78, dd (8.1, 1.6)
4’	124.1, CH_{arom}	6.98, dd (8.0, 0.8)
5’	133.3/133.2/133.1, CH_{arom}	7.23, ddd (8.5, 7.0, 1.7)
6’	119.1/118.3, CH_{arom}	6.78-6.71, m
7’	145.4, C_q	
8’	90.2, CH	4.60, s
9’	60.5/60.3, CH	3.83-3.78, m
10’	31.6, CH_2	2.37-2.2.26, m<br>2.23-1.97, m
11’	23.84/23.82, CH_2	2.23-1.97, m<br>1.88-1.77, m
12’	50.2, CH_2	3.75-3.65, m

SI-6. Feeding experiments for confirmation of biosynthetic pathway

Preparation of conditioned medium from bacterial cultures

K. oxytoca strains were cultured in 50 mL CASO-medium in 100 mL Erlenmeyer flasks for 24 h only or over a period of time from 0-48 h. Portions of 2 mL of conditioned culture broth were centrifuged at 10.000 rpm for 5 min, the supernatant was collected and sterilized through a 0.2 µm cellulose-acetate filter. The filtrate was stored at -20 °C and used for HPLC-MS analysis and cytotoxicity assays.

Extraction and HPLC-MS analysis of conditioned medium

700 µL of conditioned medium were mixed 1:1 with *n*-butanol by vortexing (20 sec) and centrifuged (10.000 rpm, 5 min). The organic extract was concentrated to dryness via vacuum centrifugation at 45 °C. Dried extracts were stored at -20 °C. For HPLC-MS analysis samples were re-extracted in MeOH (100 µL), transferred to sterile autosampler-vials (polypropylene, 250 µL) and held at 4 °C in the autosampler prior to measurement. Electrospray ionization mass spectrometry (ESI-MS) determinations were performed using an Agilent 6460 instrument after separation on an Agilent 1100 series high performance liquid chromatography (HPLC) instrument (Agilent Technologies, Waldbronn, Germany) equipped with a reversed-phase column (Shodex Asahipak ODP-50; 4.0 x 125 mm; 5 µm particle size) using mobile phases A) water incl. 0.1 % formic acid (v/v), and B) acetonitrile + incl. 0.1 % formic acid (v/v) under gradient elution conditions: 0-15 min, 10-14 % B; 15-15.5 min, 14-50 % B; 15.5-25 min, 50 % B; 25-25.1 min, 50-10 % B; and 25.1-32 min, 10 % B. ESI-MS was equipped with an Agilent Jet Stream ion source operated in positive ionization mode applying a capillary voltage of 3.5 kV; nozzle voltage of 1.0 kV; gas temperature was 350 °C, with a nitrogen gas flow of 12 L/min; sheath gas temperature of 300 °C, and nitrogen sheath gas flow of 10 L/min. Ions were recorded as protonated $[M+H]^+$ form in single ion monitoring mode where masses were set to m/z: 118.0 (indole), 138.0 (anthranilic acid), 154.0 (3-hydroxy-anthranilic acid), 219.1 (deoxy-tilimycin), 235.1 (tilimycin/culdesacin), 318.2 (deoxy-tilivalline), and 334.2 (tilivalline). For anthranilic acid and 3-hydroxy-anthranilic acid we further recorded the $[M+H-H_2O]^+$ ions due to their more efficient ionization at m/z: 136.0 (3-hydroxy-anthranilic acid – H_2O) and 120.0 (anthranilic acid – H2O). Column temperature was 40 °C; HPLC-flow was set to 0.5 mL/min; and injection volume was 1 µL.

Chemical complementation of *K. oxytoca* AHC-6 Δ*tnaA* with indole

Indole (≥99 %, Sigma Aldrich) was dissolved in water and added to 50 mL CASO-medium (500 µM final concentration). WT and Δ*tnaA* strains were cultivated in 100 mL Erlenmeyer flasks at 37 °C with shaking at 180 rpm for 24 h. Conditioned medium was extracted with *n*-butanol followed by HPLC-MS analysis and cytotoxicity assays as described in 0 and 0.

Conversion experiments - spiking of tilimycin (2), tilivalline (1) or culdesacine (3) to *K. oxytoca* AHC-6 ΔnpsA

Conversion experiments were performed to investigate the spontaneous reaction of tilimycin (**2**) to tilivalline (**1**) in the presence of indole and the degradation to culdesacin (**3**) (Figure S5).

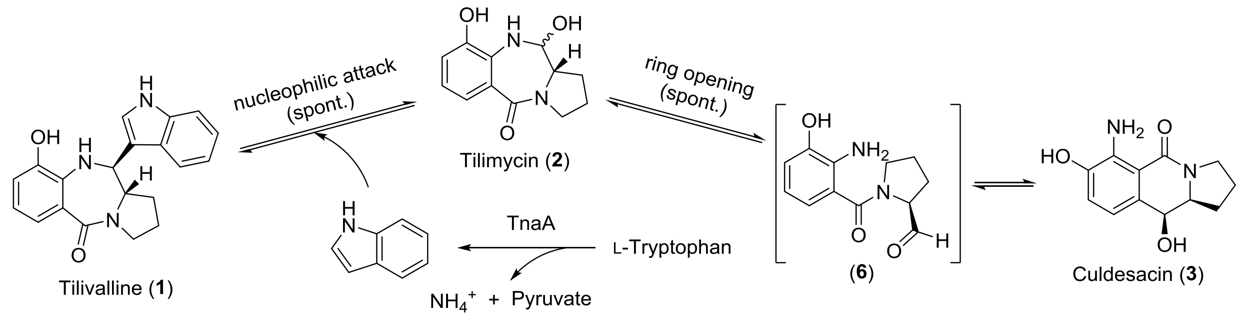

Figure S5: Proposed chemical reactions of tilimycin (**2**) to culdesacine (**3**) via spontaneous ring opening and to tilivalline (**1**) via a nucleophilic attack of free indole, released by the tryptophanase (TnaA)-catalyzed cleavage of L-tryptophan.

Metabolites were dissolved in DMSO (**1**, **3**) resp. *n*-butanol (**2**) to prepare stock solutions of 30 mM. Synthetic tilimycin (**2**) with or without indole (500 µM), tilivalline (**1**) or culdesacin (**3**) were added (final concentration of 50 µM) to either 10 mL CASO medium, a 16 h culture of AHC-6 Δ*npsA* or conditioned supernatant. The toxin-negative strain AHC-6 Δ*npsA* does not produce any of the three metabolites. All reactions were incubated at 37°C for 48 h. Samples were taken after 0, 6, 10, 24, 48 h to prepare *n*-butanol extracts for HPLC-ESI-MS to monitor the decrease or increase of **1**-**3**.

In simple CASO medium tilimycin (**2**) was degraded to culdesacin (**3**), whereas in Δ*npsA* supernatant and culture, tilivalline (**1**) was also formed due to presence of indole produced by the bacteria. The addition of synthetic indole (500 µM) and **2** to CASO medium, Δ*npsA* supernatant and culture led to similar levels of **1** and **3** for all three conditions. Spiking

experiments for **1** or **3** showed that a back reaction to **2** did not occur under biological conditions (culture or supernatant) or in simple medium (see Figure S6).

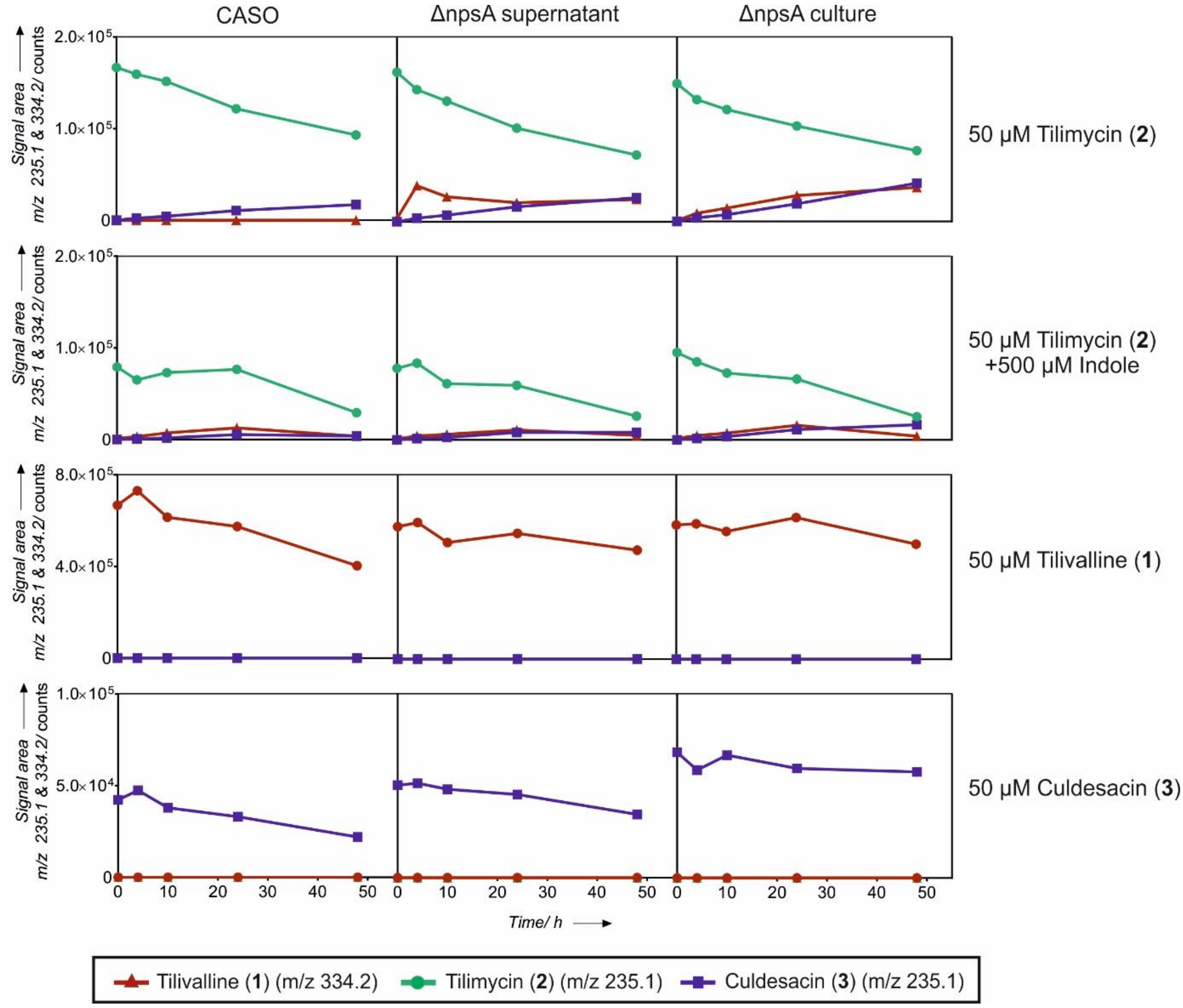

Figure S6: Spiking of 50 µM synthetic tilimycin (**2**), 50 µM synthetic tilimycin (**2**) + 500 µM indole, 50 µM synthetic tilivalline (**1**) or 50 µM culdesacin (**3**) to CASO-medium, conditioned supernatant and culture (*K.ox.* AHC-6 *ΔnpsA*, 16h). Abundance of **1**-**3** was monitored over time via HPLC-ESI-MS (m/Z 235.1 for **2** and **3**, m/z 334.2 for **1**.)

Epimerization of tilivalline (1) and conversion of tilimycin (2) and indole to tilivalline (1) *in vitro*

With the *in vitro* conversion experiments above, we could show the spontaneous conversion of tilimycin (**2**) and indole to tilivalline (**1**). Natural and synthetic tilimycin (**2**) were found to exist as a ~1:1 of *trans*/*cis*-isomers. In contrast tilivalline (**1**) was isolated from bacteria as the *trans*-isomer. In the published synthesis of tilivalline (**1**) by Shioiri et al., which used a strategy similar to the biosynthetic pathway proposed in this manuscript to access the PBD-

backbone, namely the nucleophilic attack of indole at the imine, also the *trans*-isomer was isolated as the exclusive product.[14].

In order to verify the hypothesis that tilivalline (**1**) is formed spontaneously in a stereoselective way we conducted the following experiment: The spontaneous *in vitro* conversion of tilimycin (**2**) and indole was repeated using a 200x excess of indole to increase the conversion rate. Extracts were analyzed with HPLC-MS for presence of tilivalline (**1**) and epi-tilivalline (**1a**). The HPLC-reference spectrum was achieved by epimerization of tilivalline (**1**) according the Matsumoto-protocol[15] and the tilivalline (**1**)/epi-tilivalline (**1a**) mixture was measured via HPLC-MS with the previously described method to detect the two distinct peaks for **1** and **1a**.

Epimerization was conducted according to Matsumoto et al.[15] A 10 mL Schlenk flask with magnetic stirring bar was charged with 5.2 mg (15.6 µmol, 1 eq) tilivalline (**1**) and 250 µL dry MeCN. To the solution were added 17.0 mg $ZnCl_2$ (125 µmol, 8 eq) (dried for 2 h at 160 °C in high vacuum), the flask was sealed and the colorless solution was heated to 55 °C. After 15 h the yellow solution was allowed to cool to room temperature and 750 µL saturated $NaHCO_3$ solution were added at 0 °C. The turbid mixture was diluted with 3.5 mL distilled water and extracted with EtOAc (10 mL). The organic phase was washed with H_2O (3.5 mL), dried over Na_2SO_4, filtrated, and the solvent was removed by using a rotary evaporator. The light-brown, amorphous solid obtained was stored at -22 °C and analyzed via HPLC-MS. Peaks for tilivalline (**1**) and epi-tilivalline (**1a**) could be separated and showed retention times of 21.0 min and 21.6 min, respectively (Figure S7).

For the spontaneous conversion of tilimycin (**2**) and indole to tilivalline (**1**) and epi-tilivalline (**1a**) 100 µM of **2** was added to CASO medium supplemented with 10 mM indole and incubated for 24 h at 37 °C. Metabolites were extracted with *n*-BuOH, dried under vacuum centrifugation at 45 °C and analyzed via HPLC-MS. Tilimycin (**2**) and indole spontaneously reacted to tilivalline (**1**) and culdesacin (**3**) as already described. At RT 21.6 min a small shoulder is visible which could indicate a small amount of produced epi-tilivalline (**1a,** <4 %). However, tilivalline (**1,** >96%) is the main isomer produced in the spontaneous conversion of tilimycin (**2**) with indole (Figure S7).

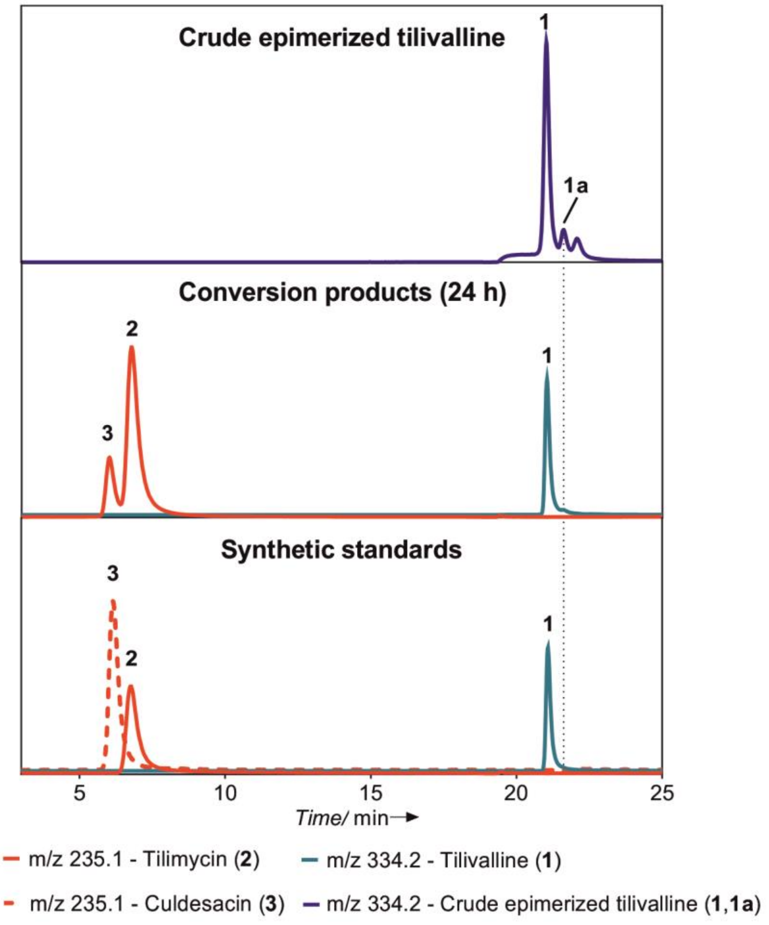

Figure S7: Spontaneous conversion of tilimycin (**2**) and indole to 96 % tilivalline (**1**), 4 % epi-tilivalline (**1a**) and culdesacin (**3**) in CASO-medium. HPLC-ESI-MS chromatograms of synthetic standards of tilivalline (**1**) – m/z 334.2 (light blue), crude epimerized tilivalline (**1**, **1a**) – m/z 334.2 (*cis/trans,* dark blue), tilimycin (**2**) and culdesacin (**3**) – m/z 235.1 (red).

Feeding of 3-hydroxyanthranilic acid (3HAA) and anthranilic acid (AA) to all *K. oxytoca* knockout strains

We hypothesized that 3HAA could be the substrate of the non-ribosomal peptide synthase NpsA. Pathway a, proposed the formation of 3HAA from 2-amino-2-deoxychorismate (ADIC) to *trans*-2,3-dihydro-3-hydroxaanthranilic acid (DHHA), whereas in pathway b, AA would be the precursor 3HAA (Figure S8).

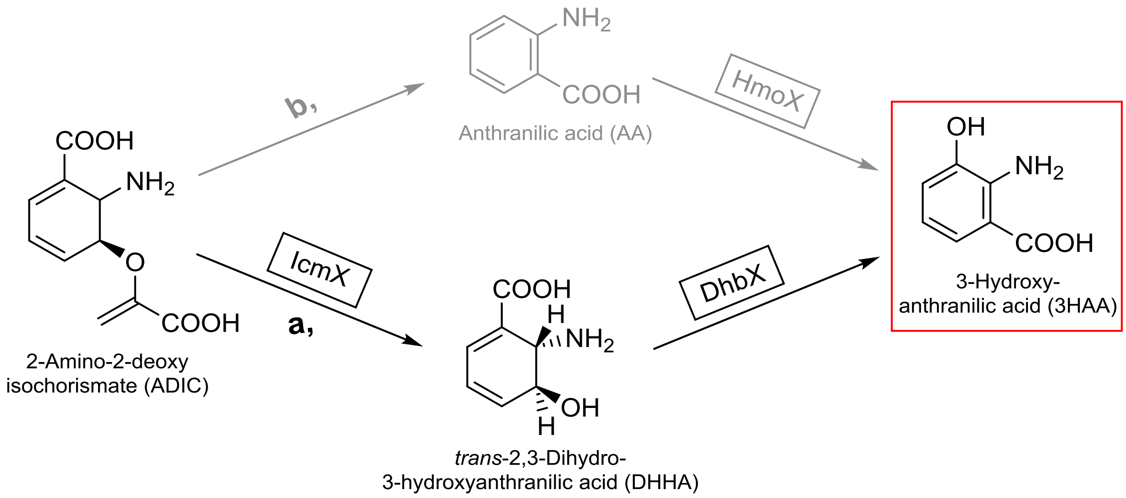

Figure S8: Proposed chemical reactions of 2-amino-2-deoxychorismate (ADIC) to 3HAA; pathway **a,** via trans-2,3-dihydro-3-hydroxaanthranilic acid (DHHA); pathway **b,** via AA; IcmX: isochorismatase, DhbX: 2,3-dihydro-dehydrogenase, HmoX: 4-hydroxyphenyl acetate-3-monoxygenase.

To test these hypotheses we performed feeding experiment adding 3HAA or AA to various mutant strains of *K. oxytoca* (AHC-6 WT, Δ*npsA*, Δ*aroB*, Δ*aroX*, Δ*adsX*, Δ*icmX*, Δ*dhbX,* ΔΔ*hmoX*). Stock solutions of 4 mM and 35 mM (H_2O with 1% DMSO) for 3HAA and AA were prepared, respectively. Final concentrations of 200 µM were added to 50 mL of bacterial cultures and incubated for 24h at 37°C with shaking at 180 rpm. After 24 h conditioned medium and *n*-butanol extracts were prepared for HPLC-ESI-MS and cytotoxicity assays.

SI-7. Cell culture assay

Culturing methods, growth conditions and media for cell cultures

Human cell lines used in this study were HeLa cells (American Type Culture Collection). The cells were cultivated in DMEM medium (4500 mg/L glucose), supplemented with 10 % FBS, 100 µg/mL penicillin and 100 µg/mL streptomycin, at 37 °C with 5 % CO_2. All media and additives were purchased from Invitrogen.

Cytotoxicity assay of bacterial supernatant and IC_{50} determination of metabolites 1-3

A modified MTT cytotoxicity assay applied conditioned medium of *K. oxytoca* strains to HeLa cells as described previously.[4, 16] Variation in cell viability was normalized to CASO medium as control.

For IC_{50} measurements HeLa cells were treated with serial dilutions of tilivalline (**1**), tilimycin (**2**) or culdesacin (**3**) in cell culture medium. Stock solutions (30 mM) were prepared in DMSO or *n*-butanol. For normalization of cell viability equivalent concentrations of DMSO or *n*-butanol were applied. IC_{50} values were obtained by sigmoidal curve fitting and values represent the mean ± SD of three independent experiments (see Figure S9).

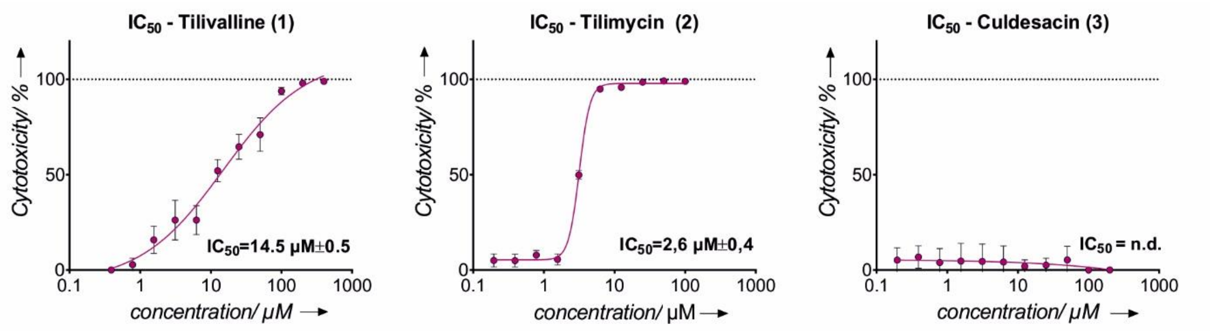

Figure S9: IC_{50} values for tilivalline (**1**), tilimycin (**2**), and culdesacin (**3**) (n=3 each).

SI-8. NMR and ATR-IR spectra

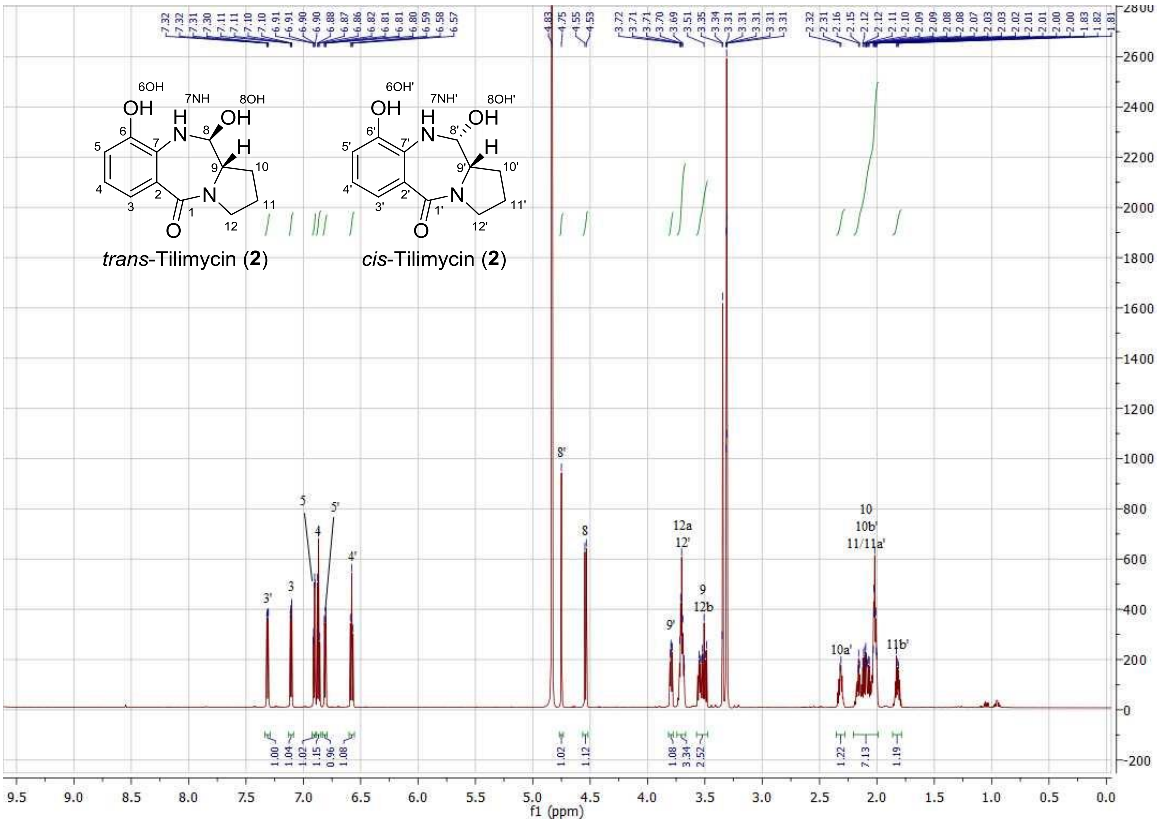

Proton NMR spectrum (700 MHz, MeOD-d_4) of natural *cis*/*trans*-tilimycin (**2**).

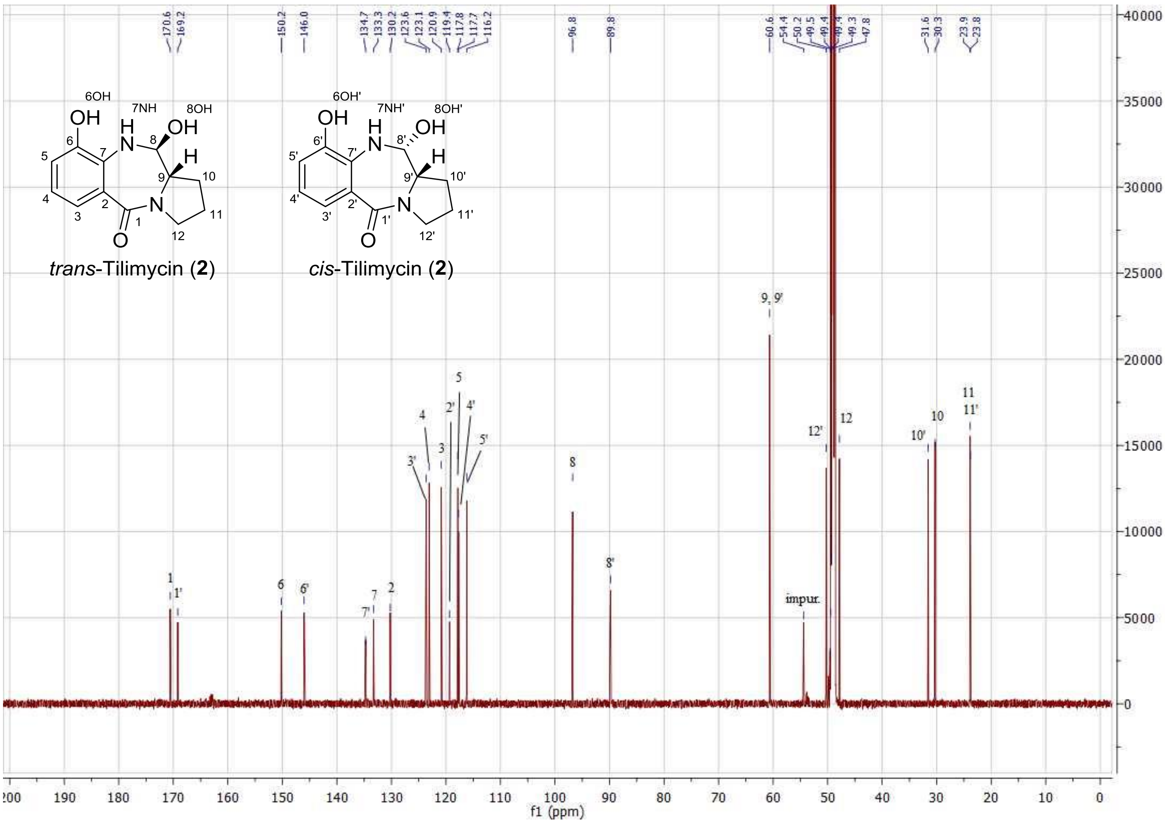

Carbon NMR spectrum (175 MHz, MeOD-d_4) of natural *cis*/*trans*-tilimycin (**2**).

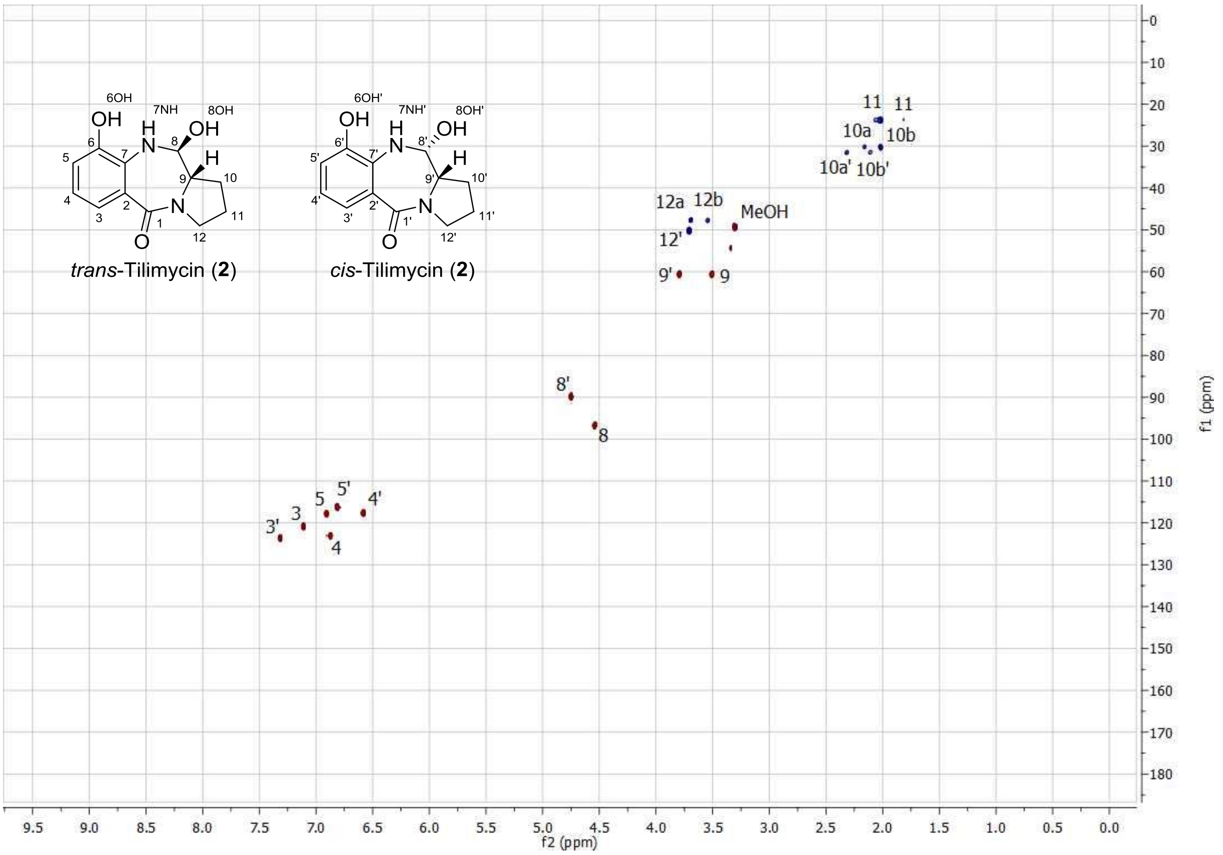

HSQC NMR spectrum (700 MHz, MeOD-d_4) of natural *cis*/*trans*-tilimycin (**2**).

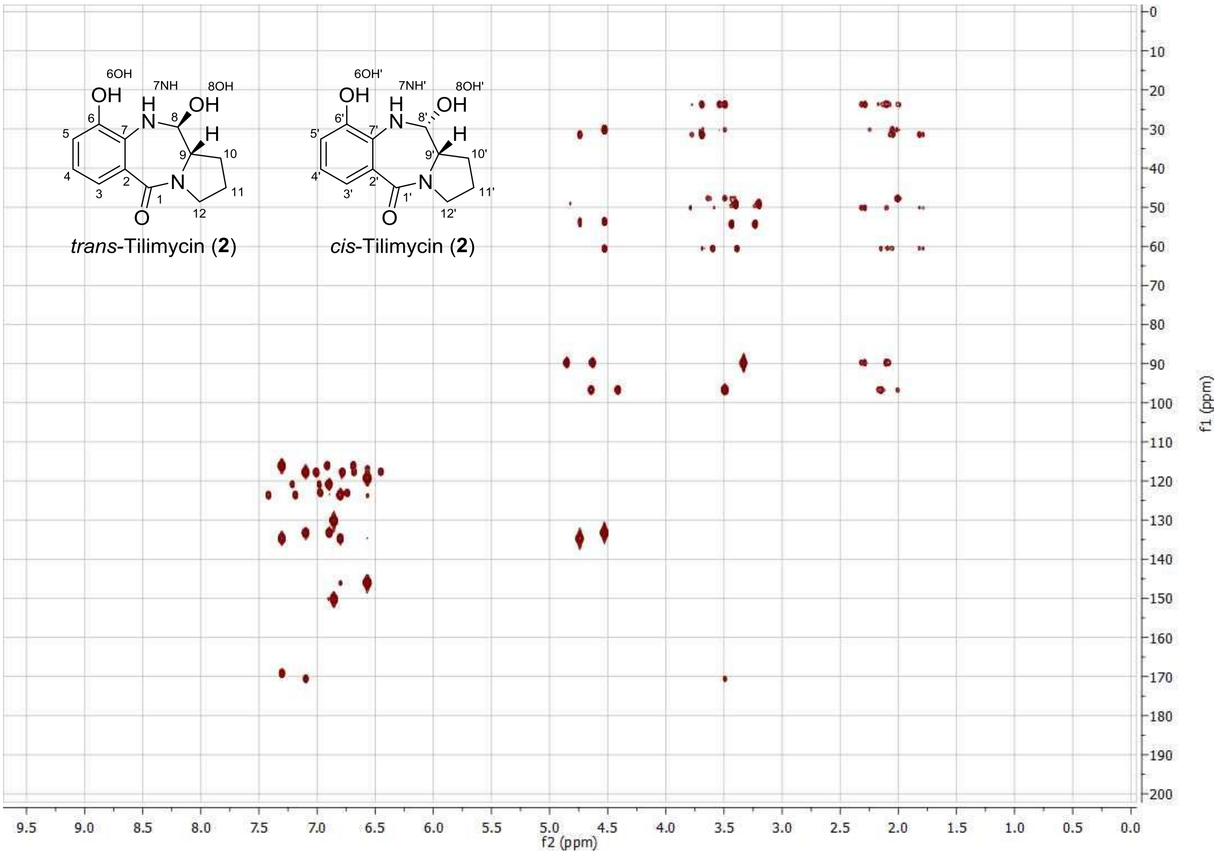

HMBC NMR spectrum (700 MHz, MeOD-d_4) of natural *cis*/*trans*-tilimycin (**2**).

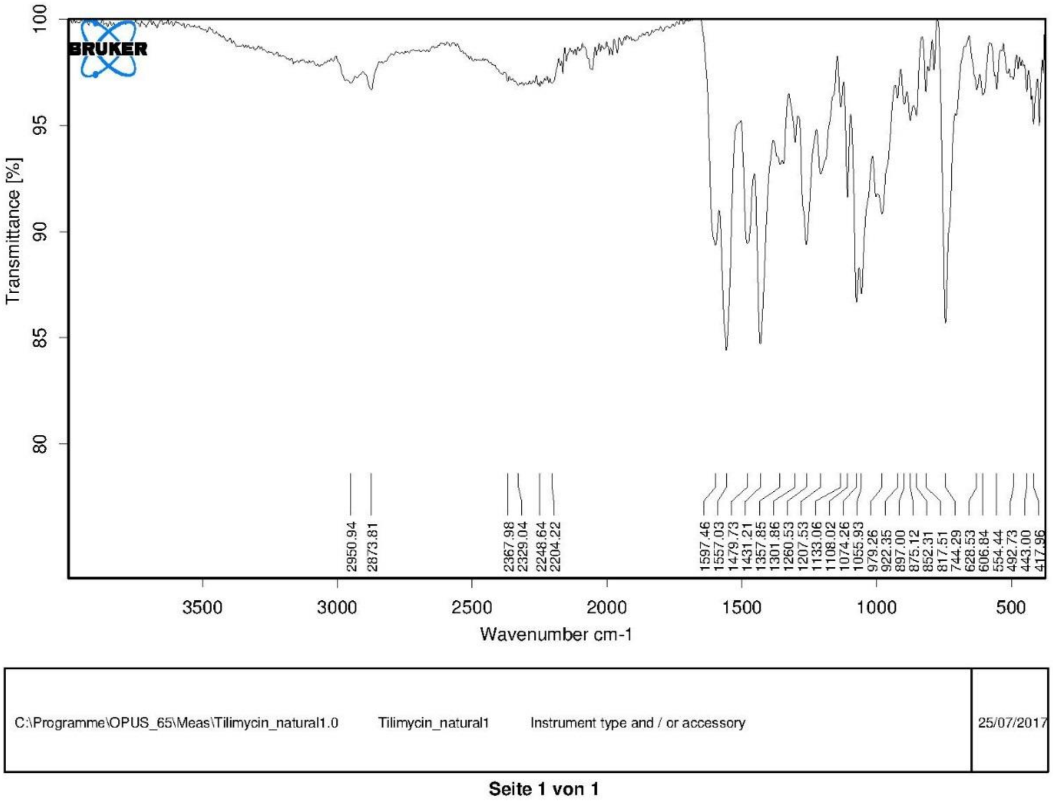

ATR-IR spectrum of natural *cis*/*trans*-tilimycin (**2**).

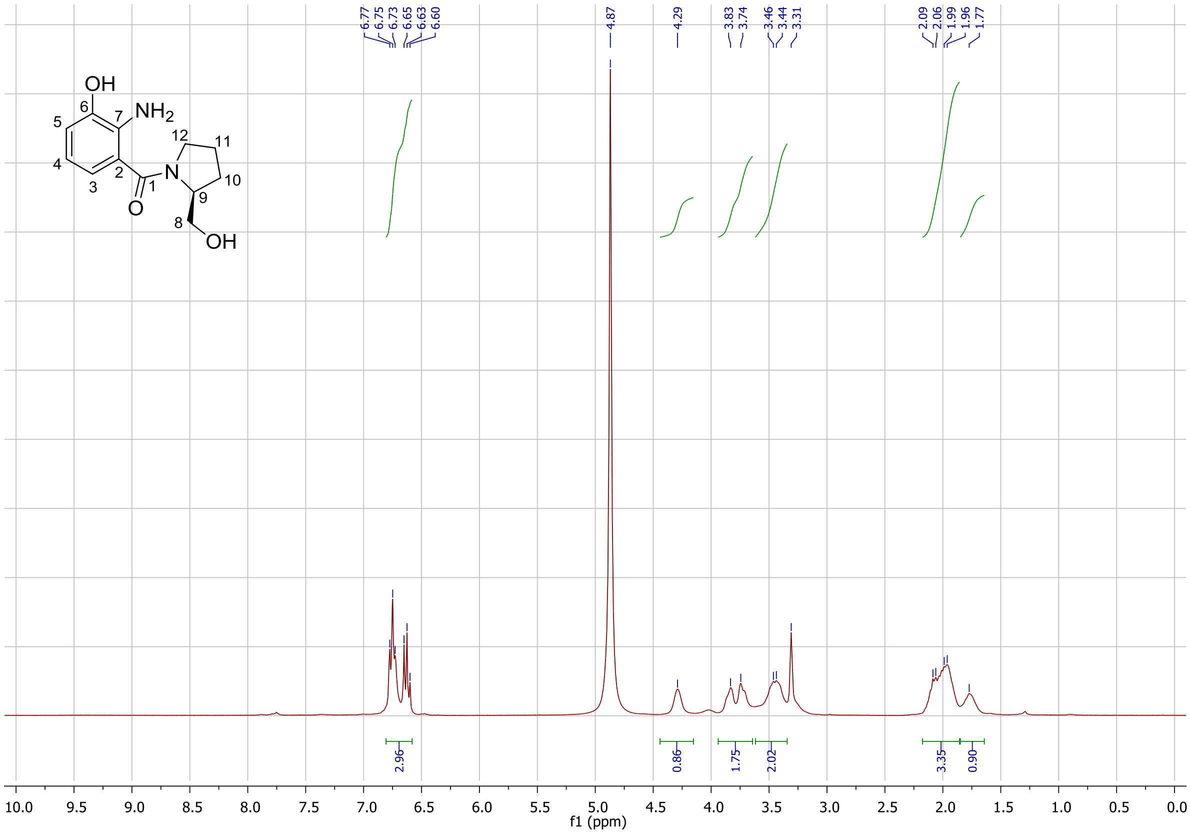

Proton NMR spectrum (300 MHz, MeOD-d$_4$) of (*S*)-(2-amino-3-hydroxyphenyl)(2-(hydroxymethyl)-pyrrolidin-1-yl)methanone.

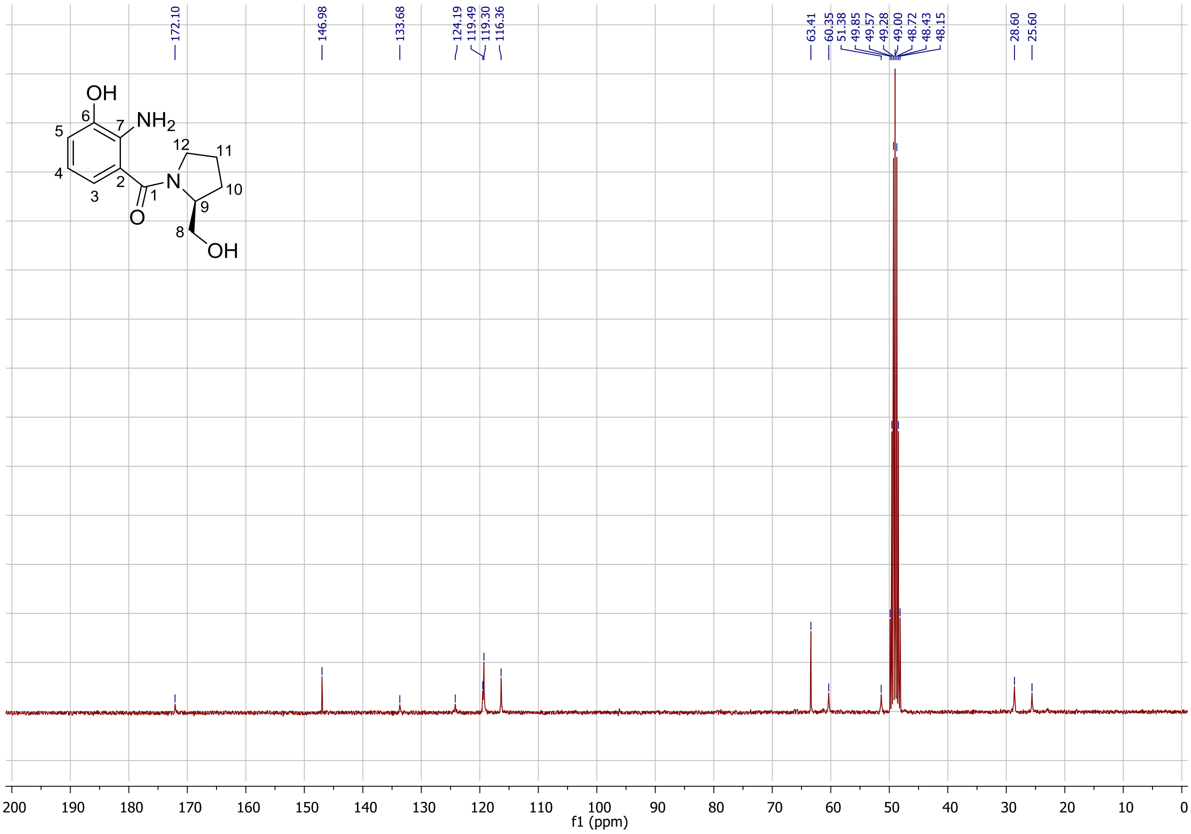

Carbon NMR spectrum (75 MHz, MeOD-d$_4$) of (*S*)-(2-amino-3-hydroxyphenyl)(2-(hydroxymethyl)-pyrrolidin-1-yl)methanone.

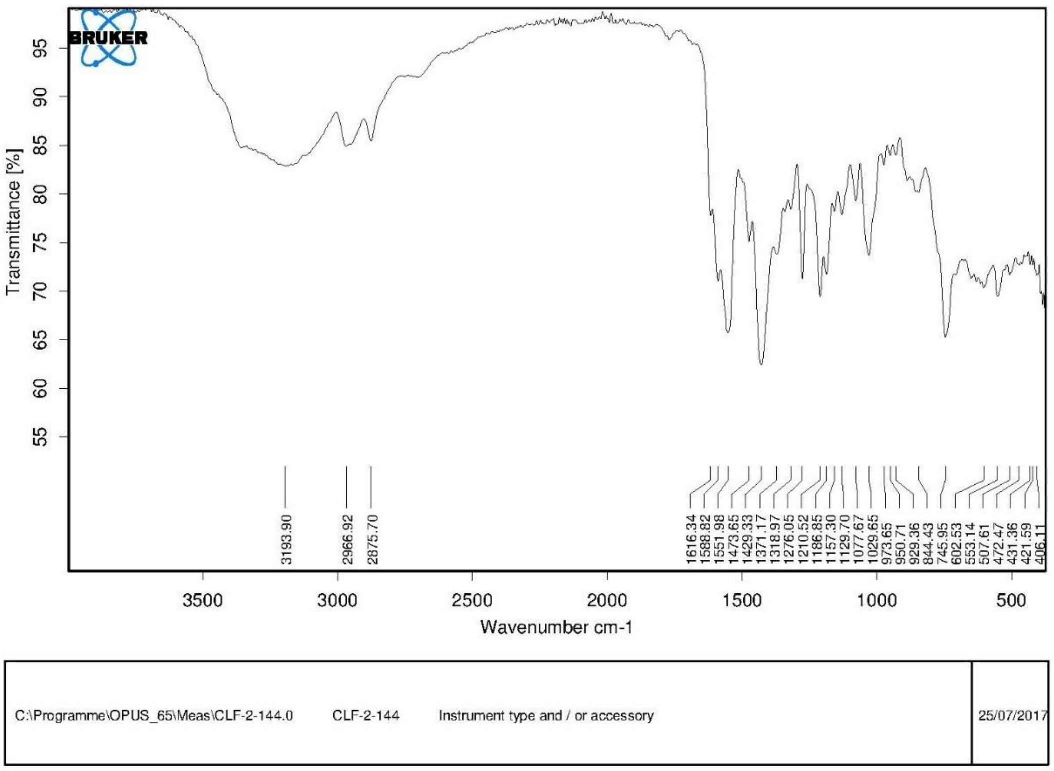

Seite 1 von 1

ATR-IR spectrum of (*S*)-(2-amino-3-hydroxyphenyl)(2-(hydroxymethyl)-pyrrolidin-1-yl)methanone.

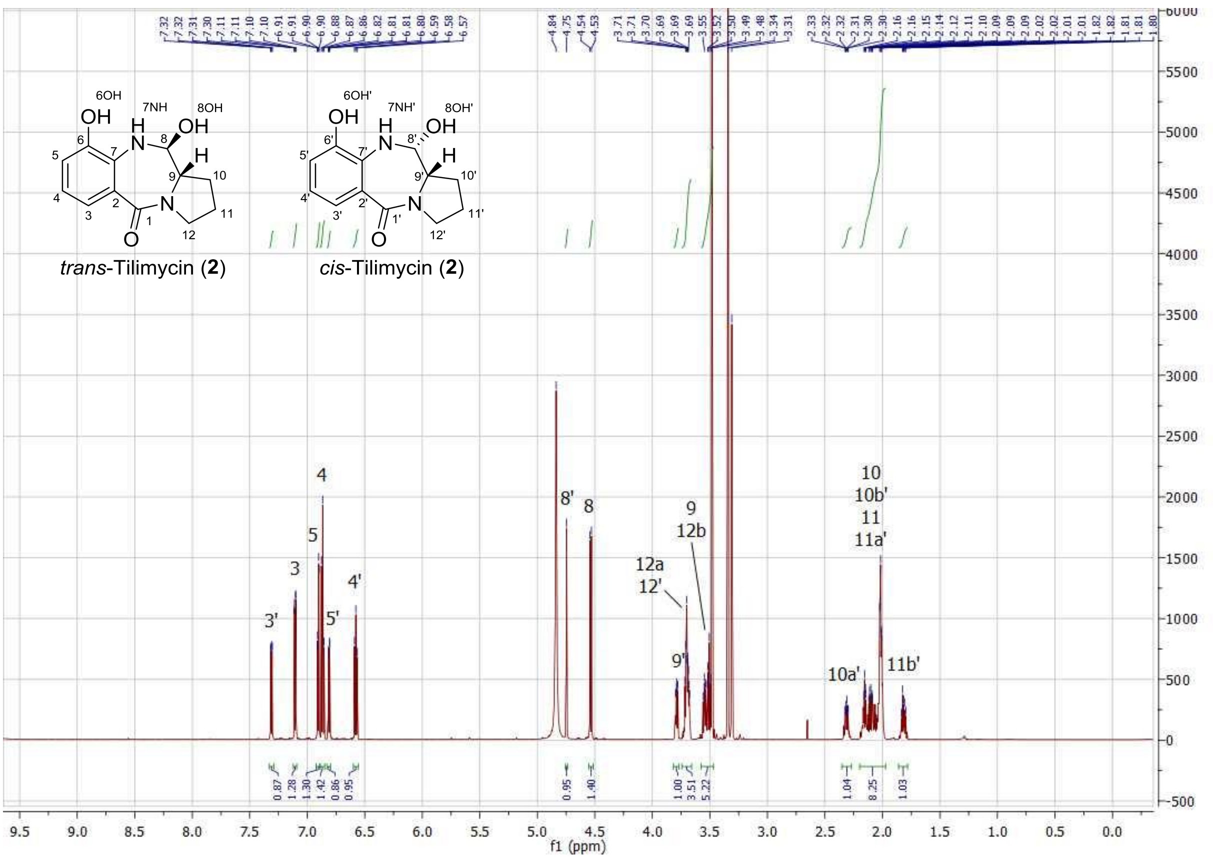

Proton NMR spectrum (700 MHz, MeOD-d$_4$) of synthetic *cis*/*trans*-tilimycin (**2**).

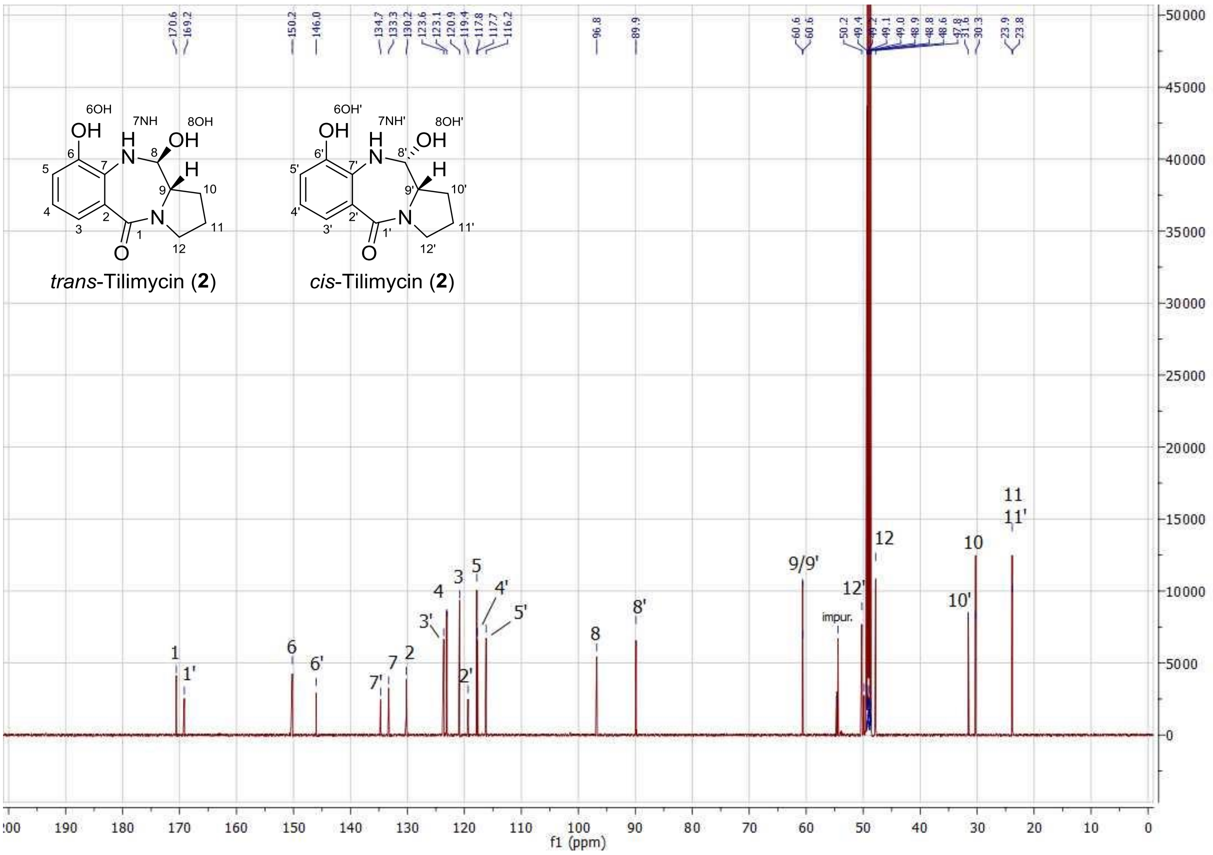

Carbon NMR spectrum (175 MHz, MeOD-d$_4$) of synthetic *cis*/*trans*-tilimycin (**2**).

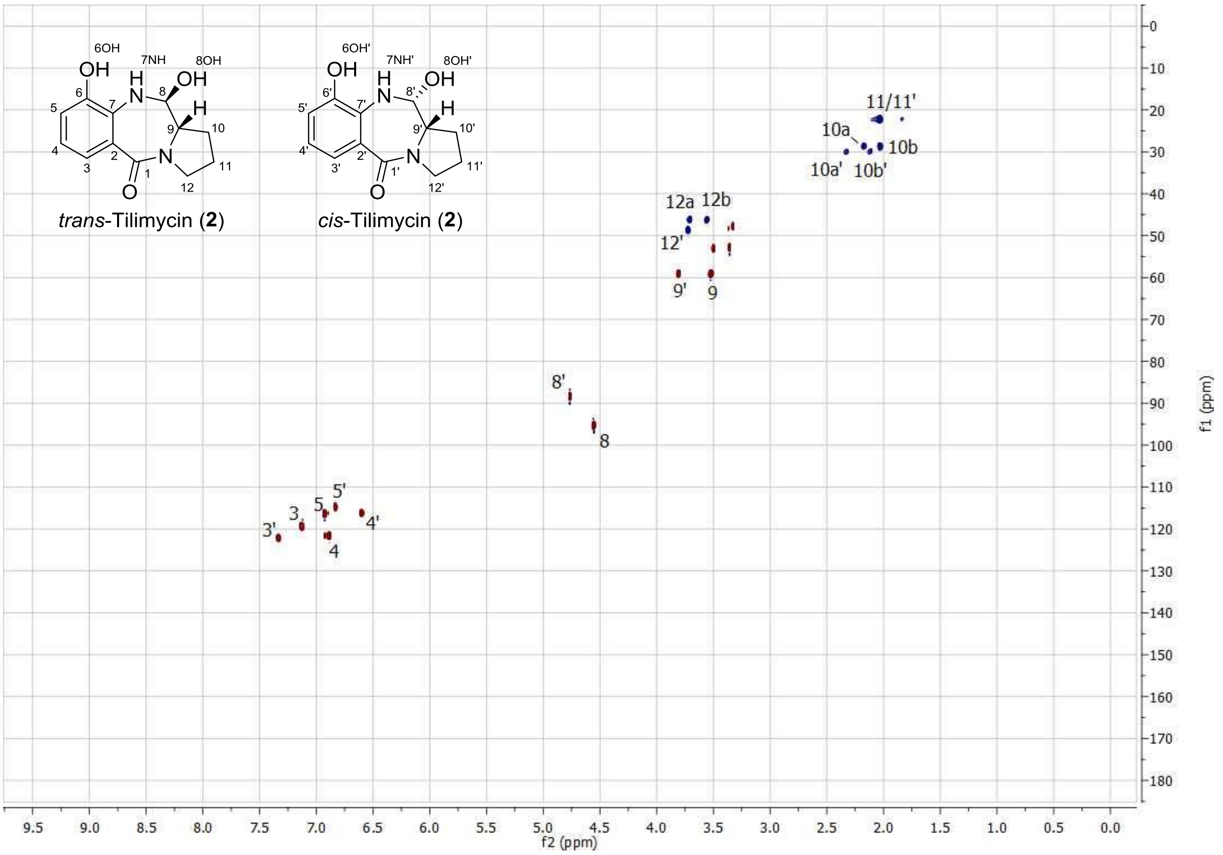

HSQC NMR spectrum (700 MHz, MeOD-d_4) of synthetic *cis*/*trans*-tilimycin (**2**).

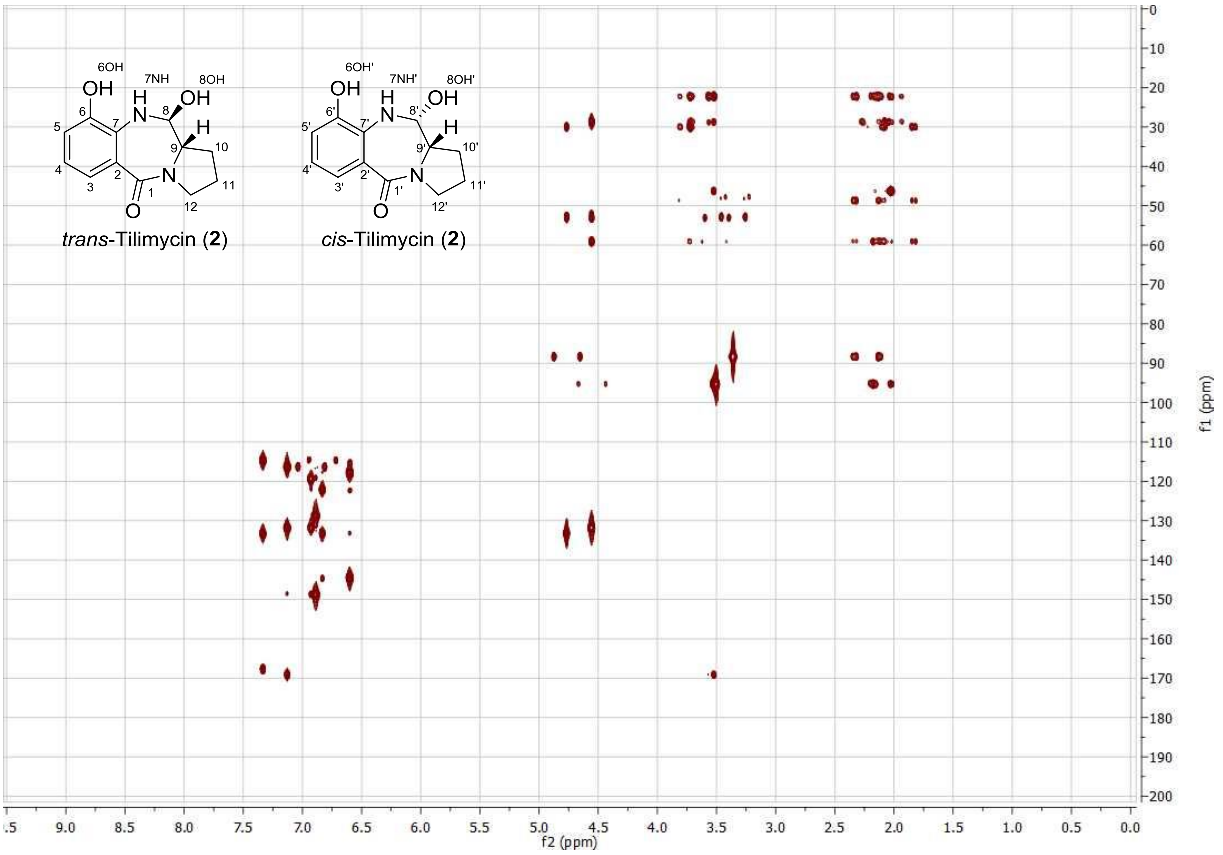

HMBC NMR spectrum (700 MHz, MeOD-d_4) of synthetic *cis*/*trans*-tilimycin (**2**).

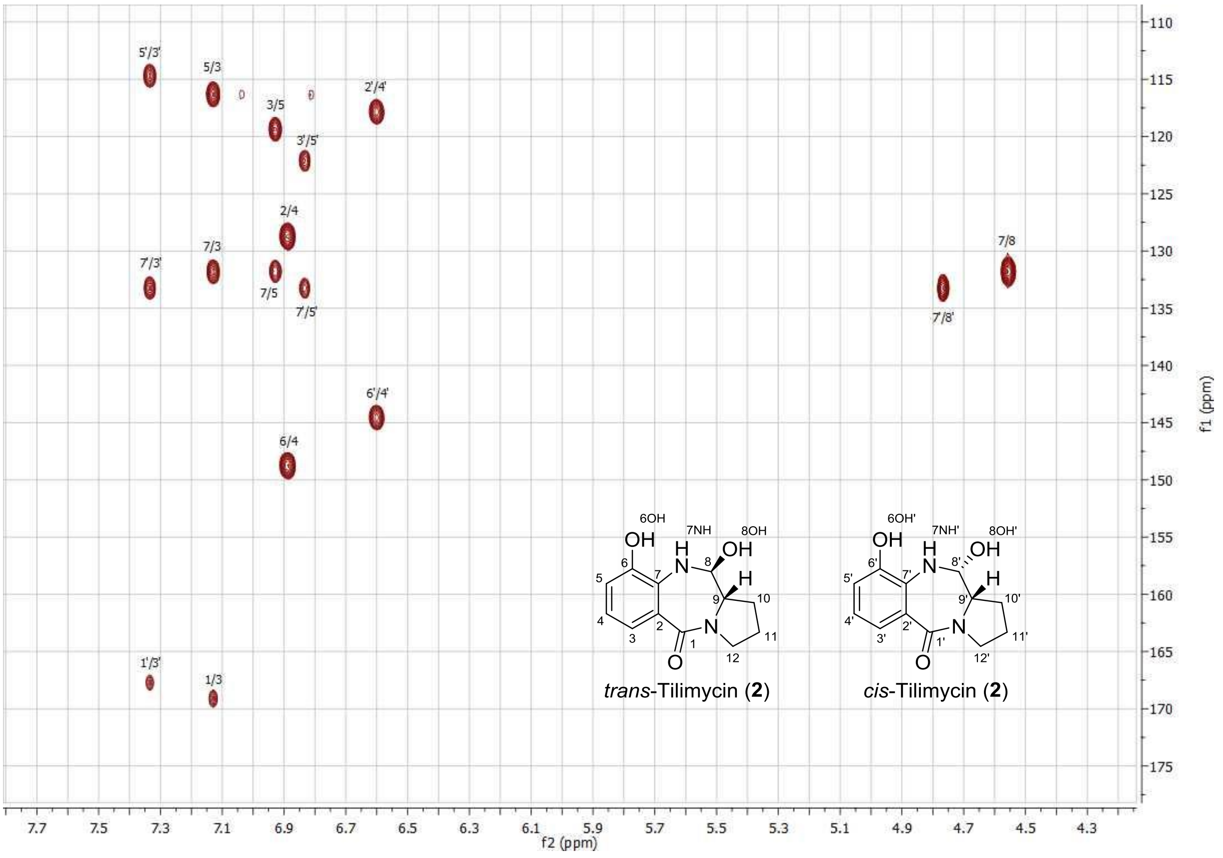

HMBC-NMR spectrum of synthetic *cis*/*trans*-tilimycin (**2**) (7.7 – 4.3 ppm).

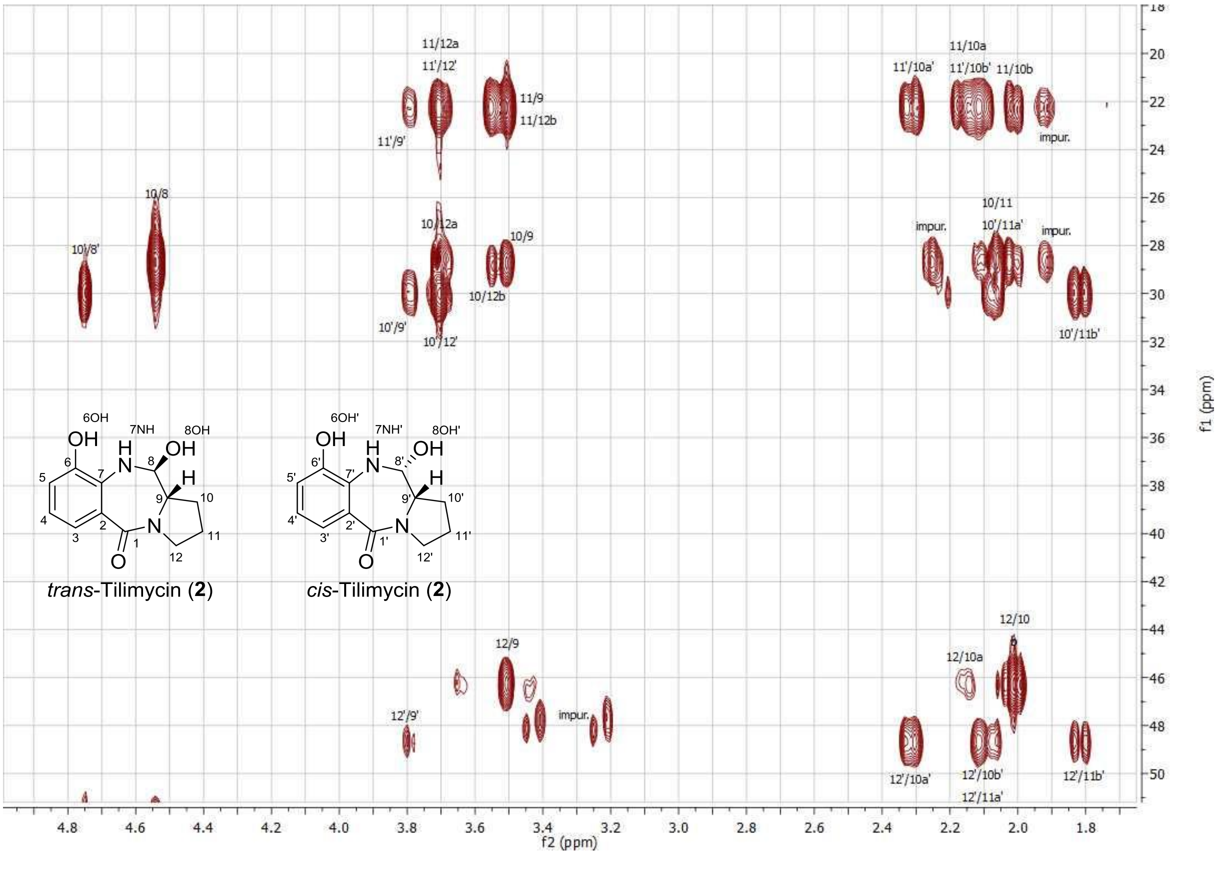

HMBC-NMR spectrum of synthetic *cis*/*trans*-tilimycin (**2**) (4.8 – 1.8 ppm).

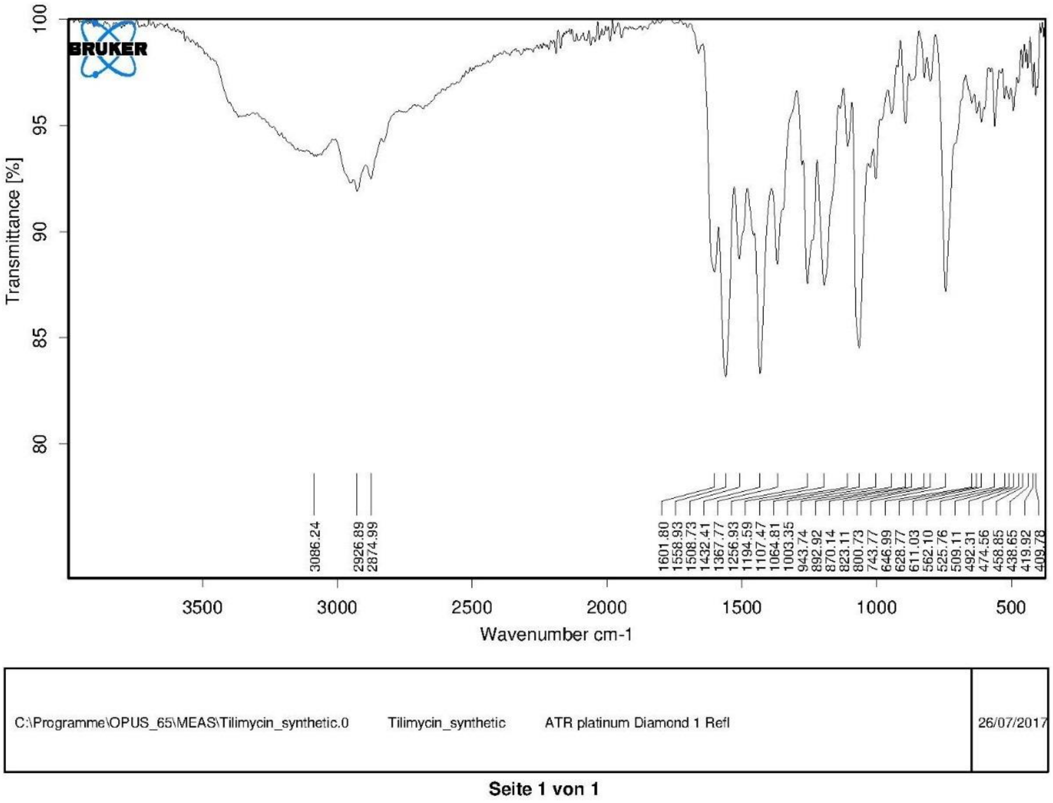

ATR-IR spectrum of synthetic *cis*/*trans*-tilimycin (**2**).

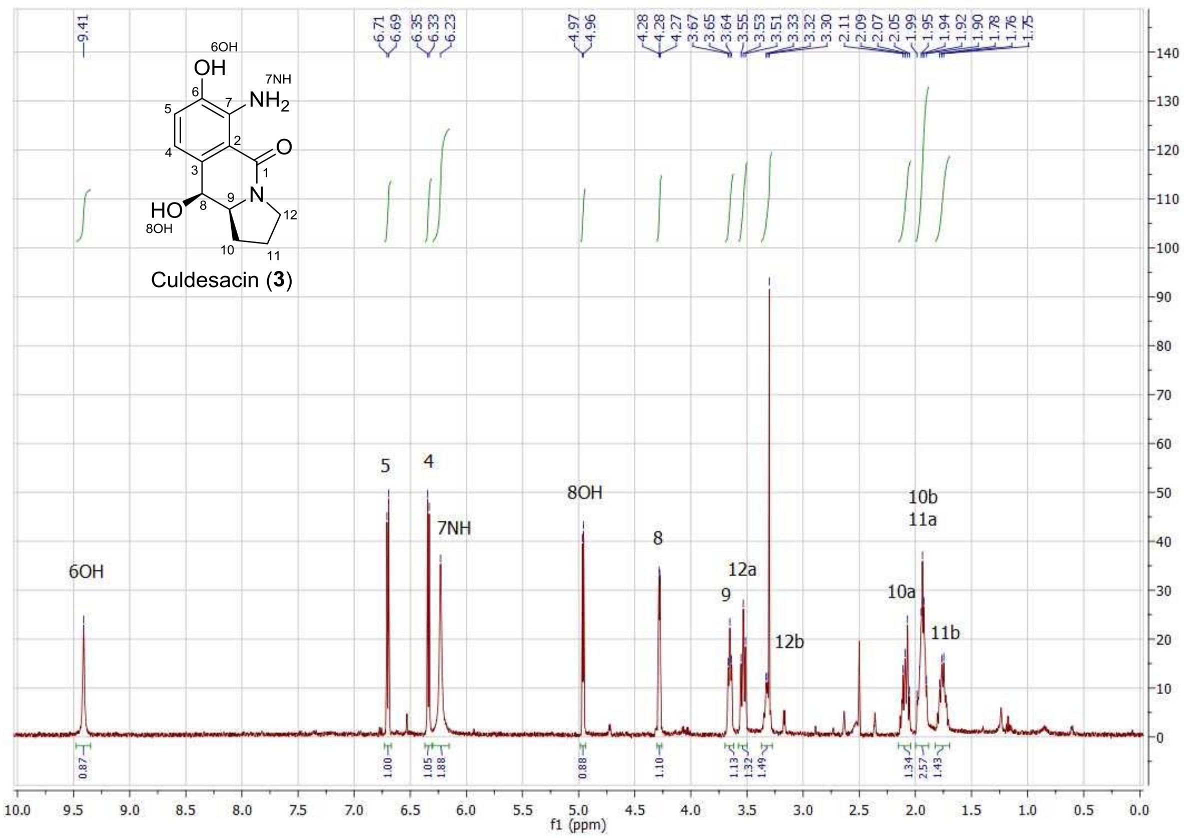

Proton NMR spectrum (^{1}H 500 MHz, DMSO-d$_6$) of natural culdesacin (**3**).

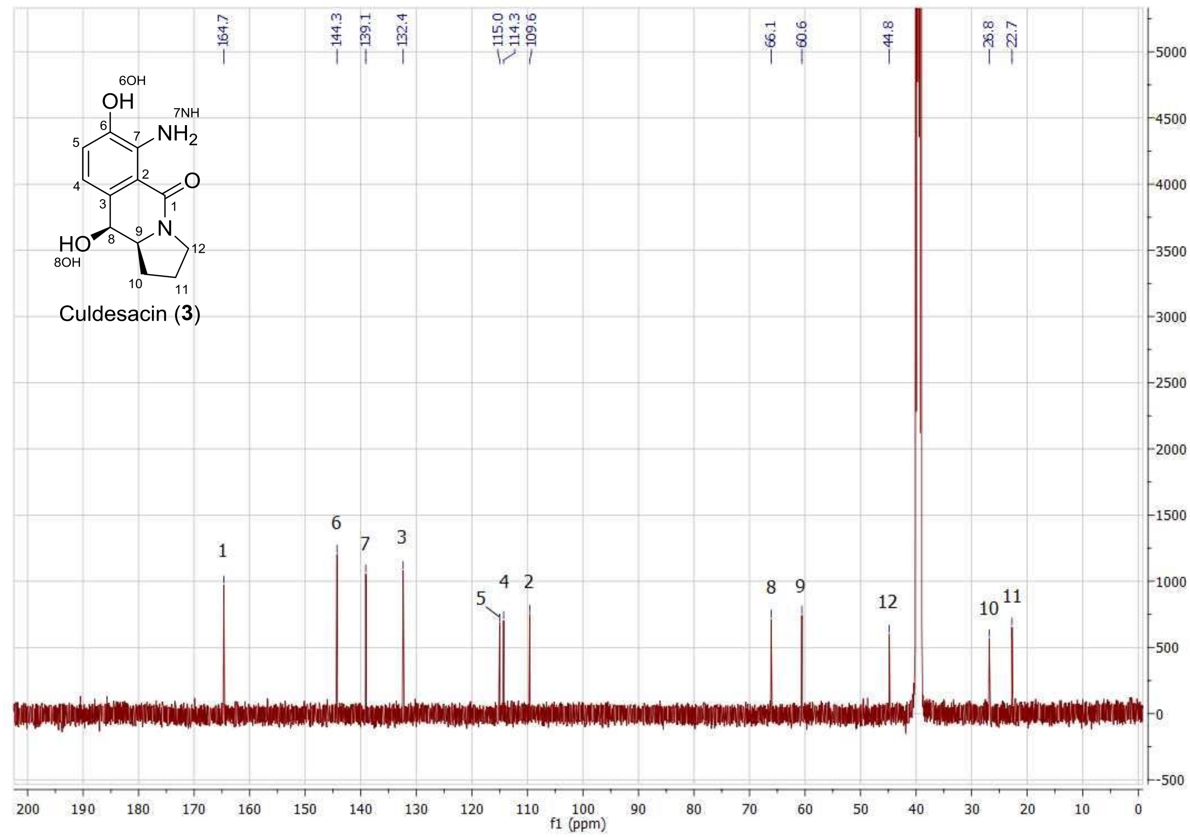

Carbon NMR spectrum (126 MHz, DMSO-d$_6$) of natural culdesacin (**3**).

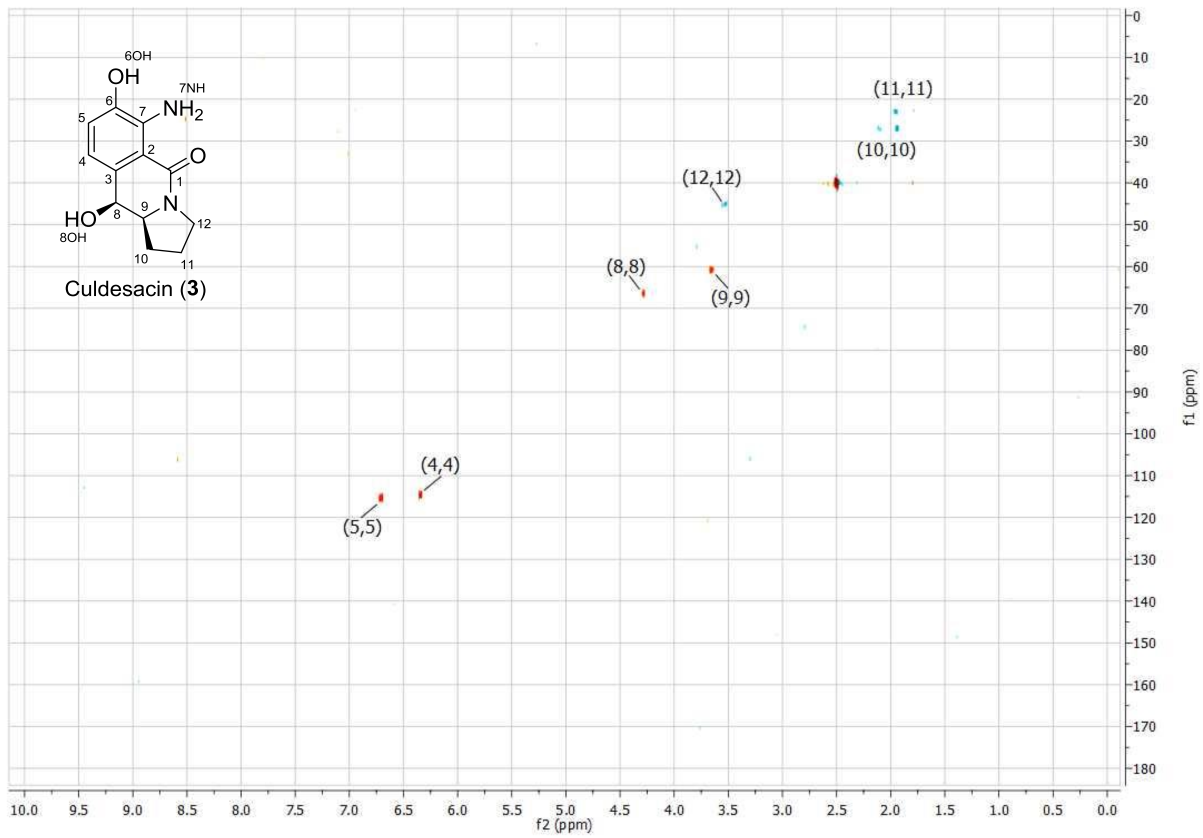

HSQC NMR spectrum (500 MHz, DMSO-d_6) of natural culdesacin (**3**).

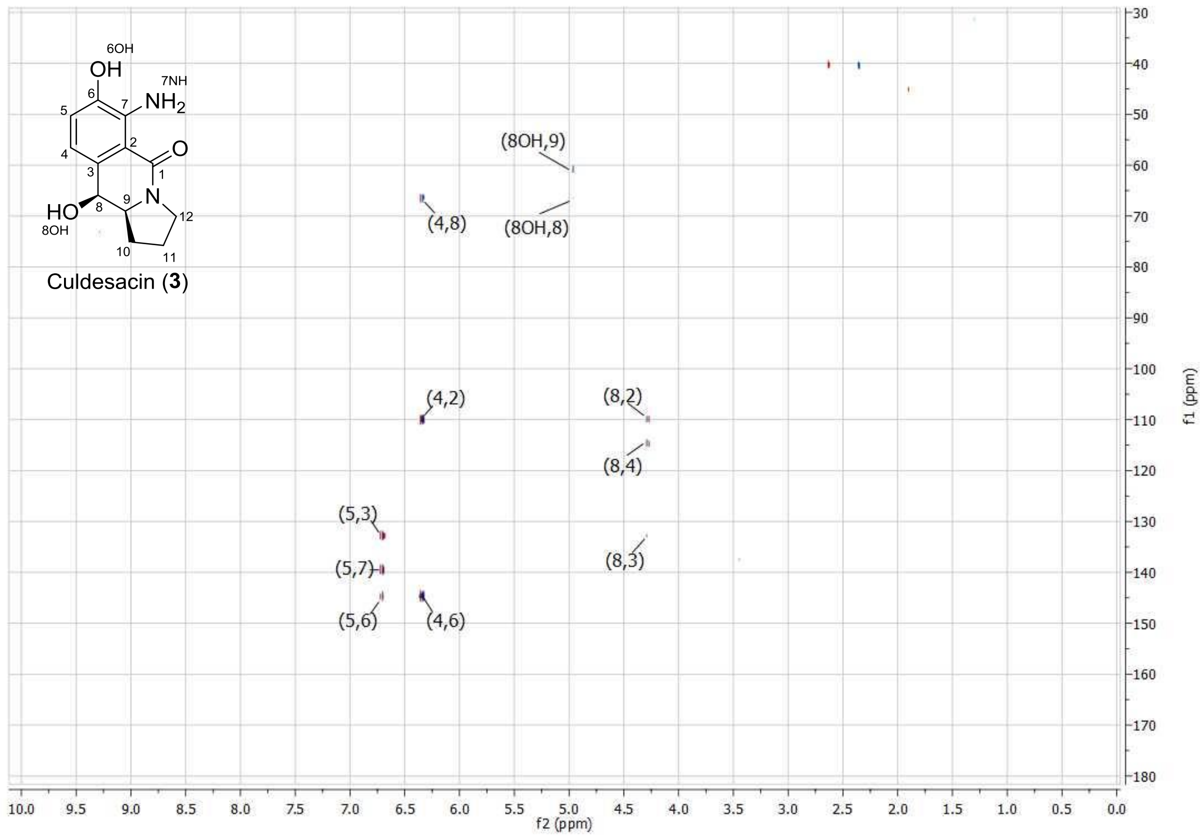

HMBC NMR spectrum (500 MHz, DMSO-d_6) of natural culdesacin (**3**).

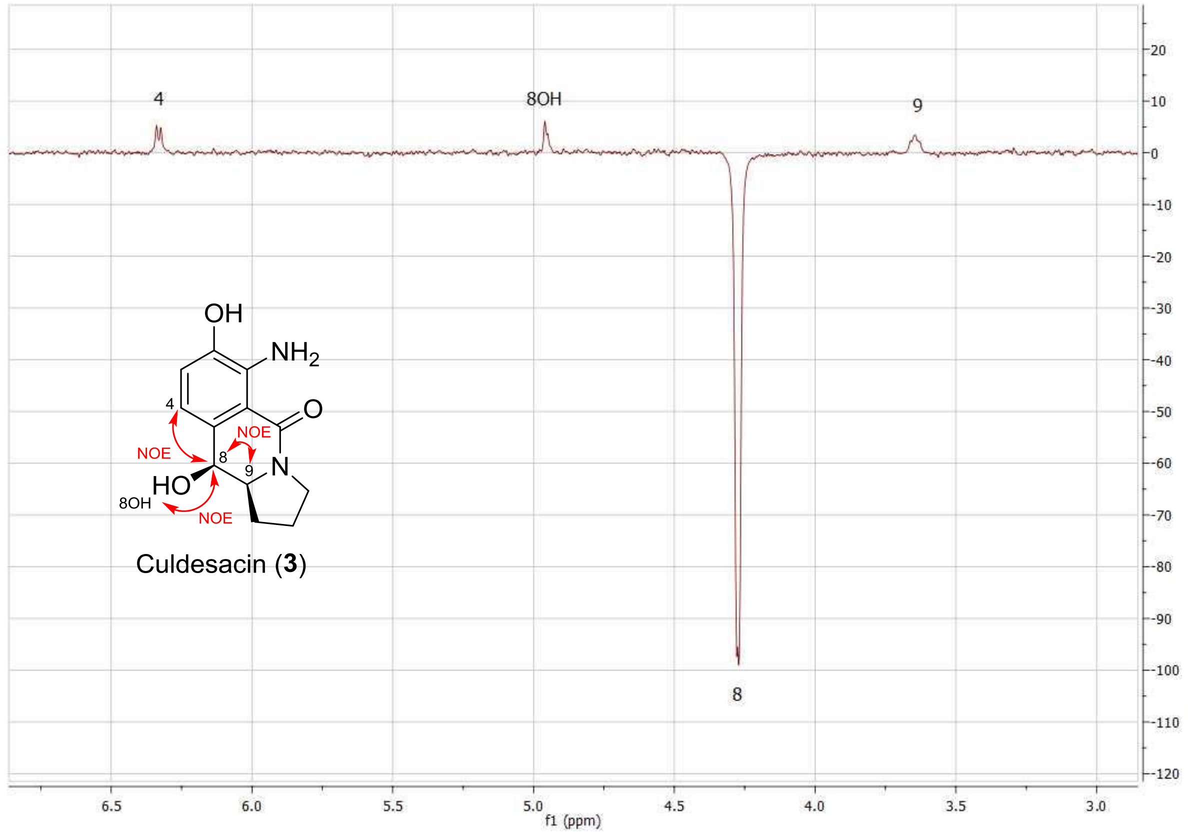

NOESY NMR spectrum (^{1}H 500 MHz, DMSO-d_6, selective band center: 4.29 ppm, width: 41.2 Hz) of natural culdesacin (**3**).

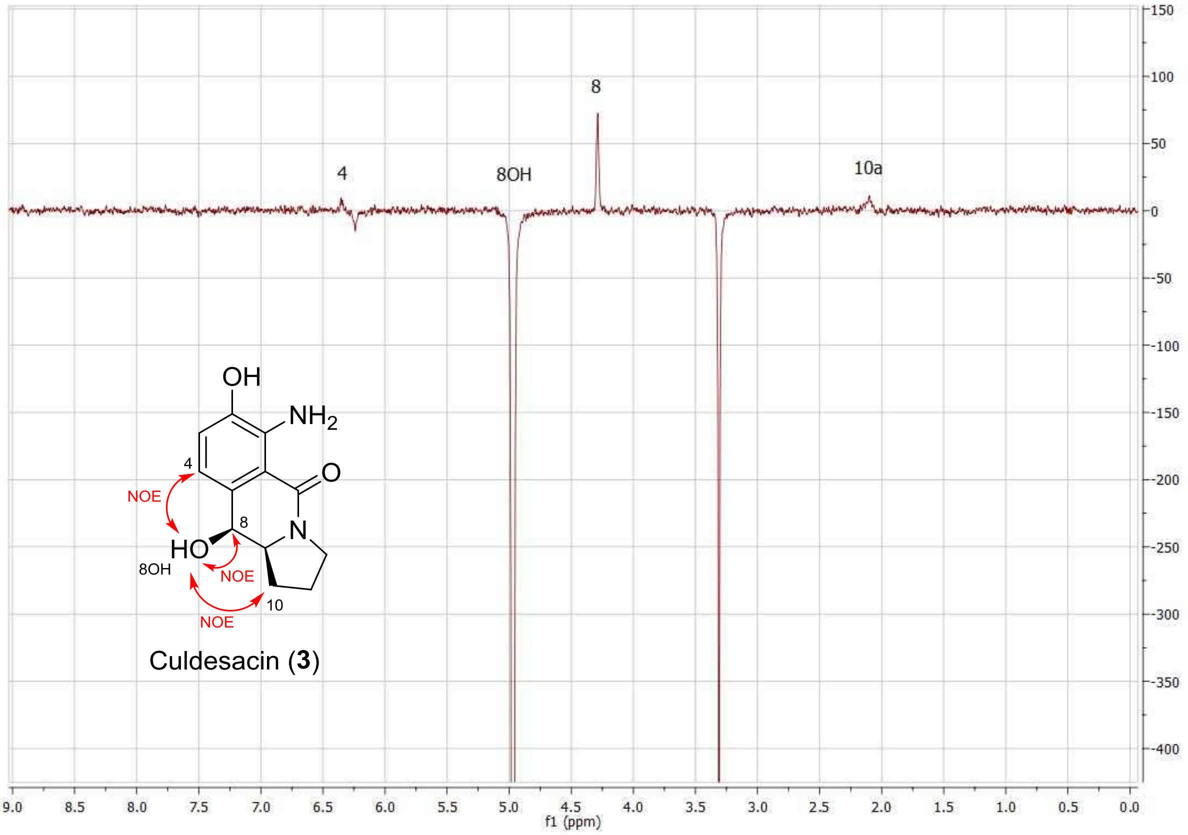

NOESY NMR spectrum (^{1}H 500 MHz, DMSO-d_6, selective band center: 4.97 ppm, width: 27.2 Hz) of natural culdesacin (**3**).

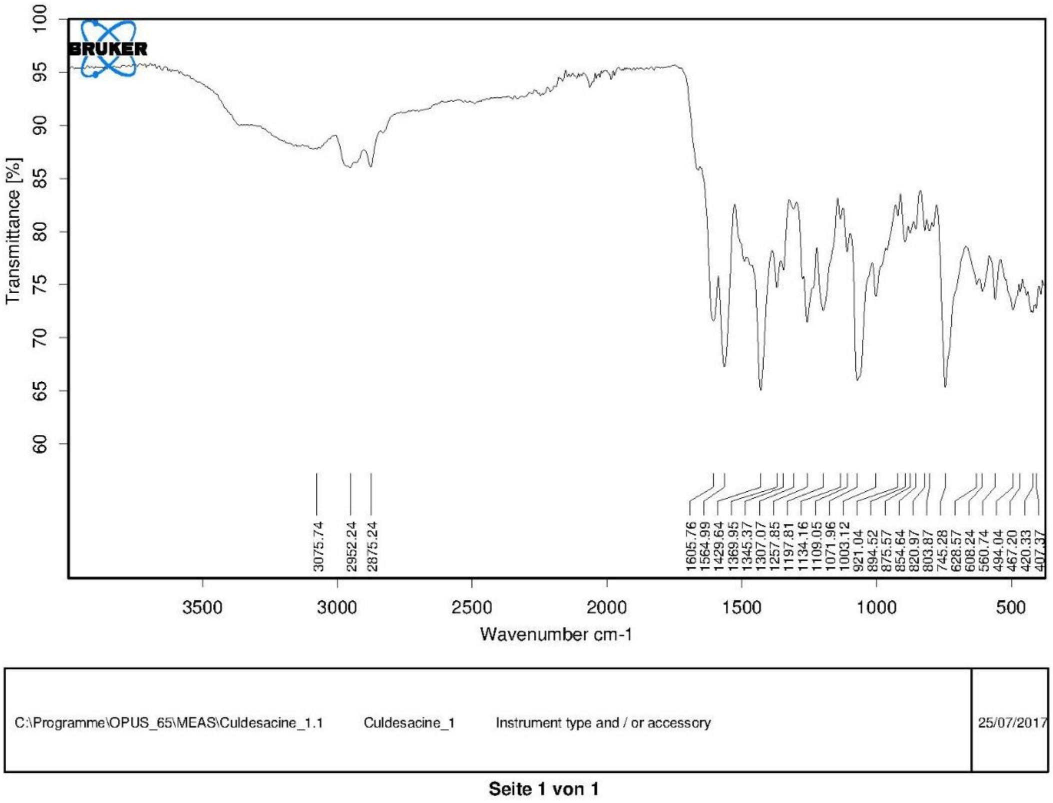

ATR-IR spectrum of natural culdesacin (**3**).

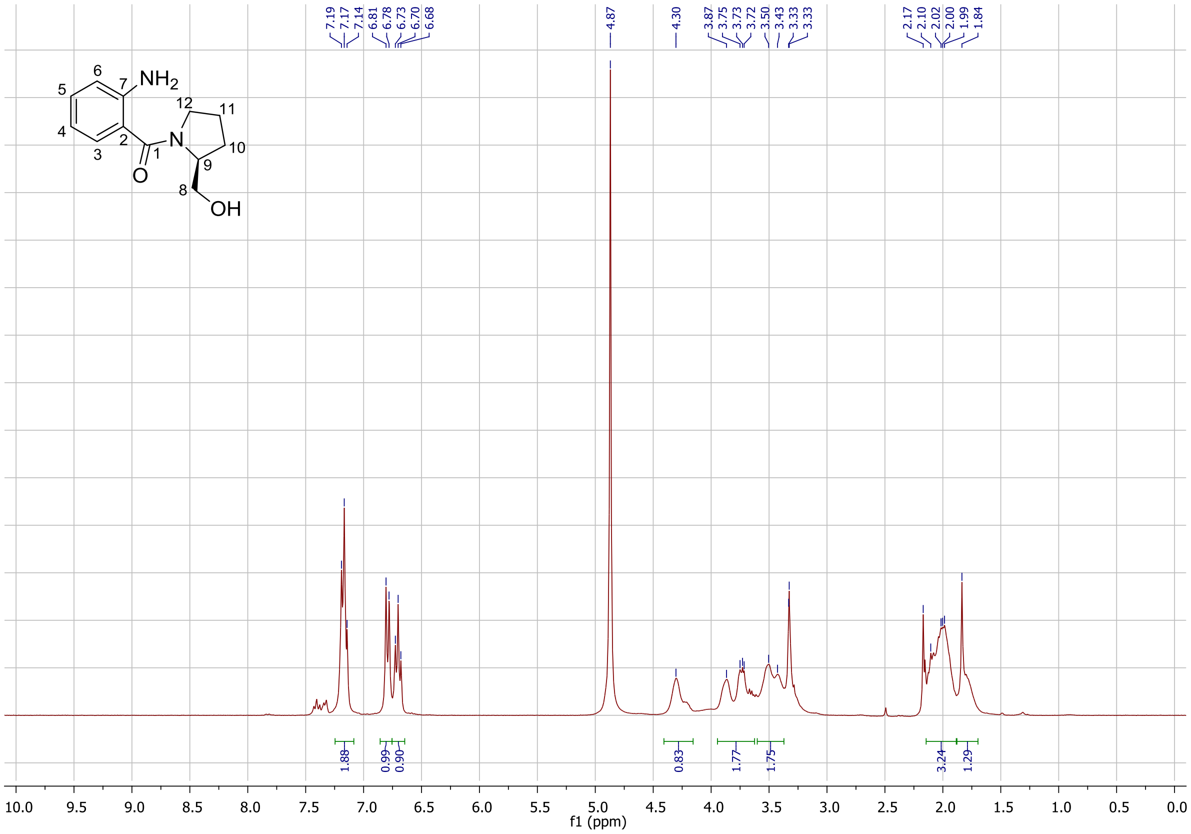

Proton NMR spectrum (300 MHz, MeOD-d$_4$) of (*S*)-(2-aminophenyl)(2-(hydroxymethyl)pyrrolidin-1-yl)methanone.

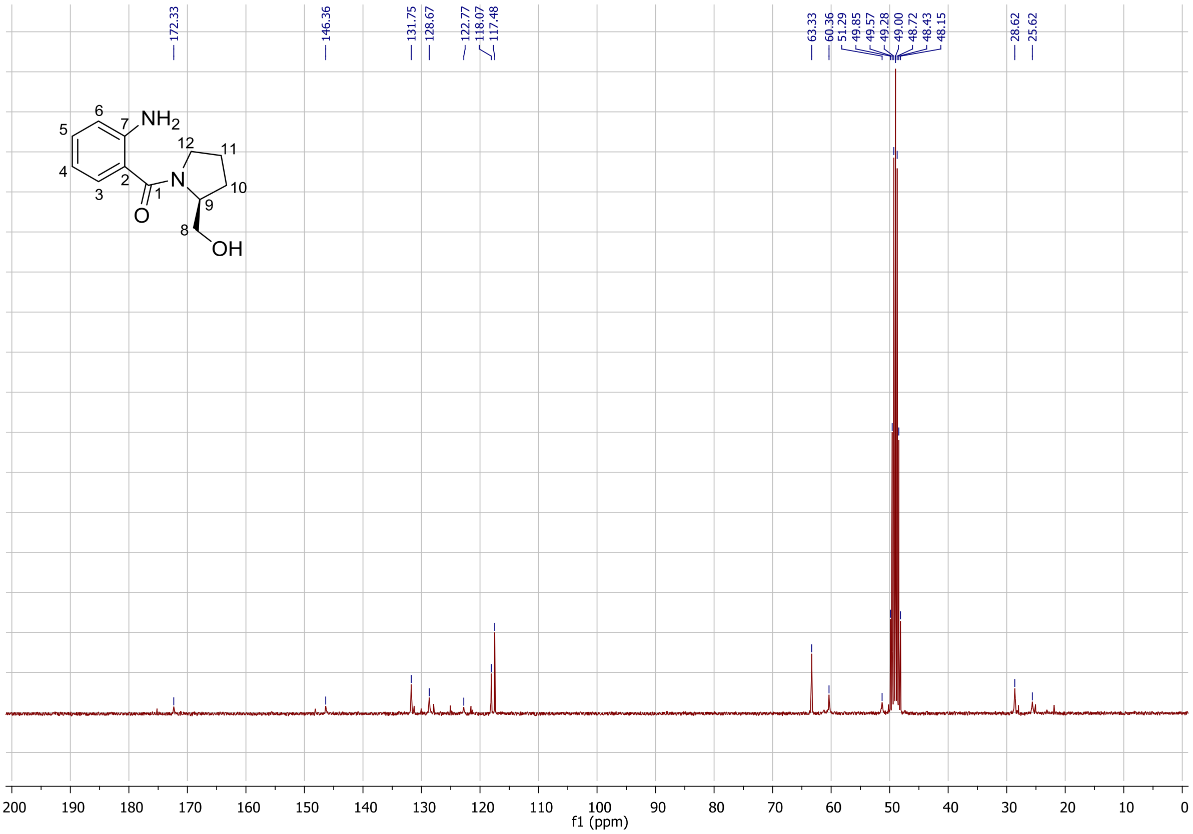

Carbon NMR spectrum (75 MHz, MeOD-d$_4$) of (*S*)-(2-aminophenyl)(2-(hydroxymethyl)pyrrolidin-1-yl)methanone.

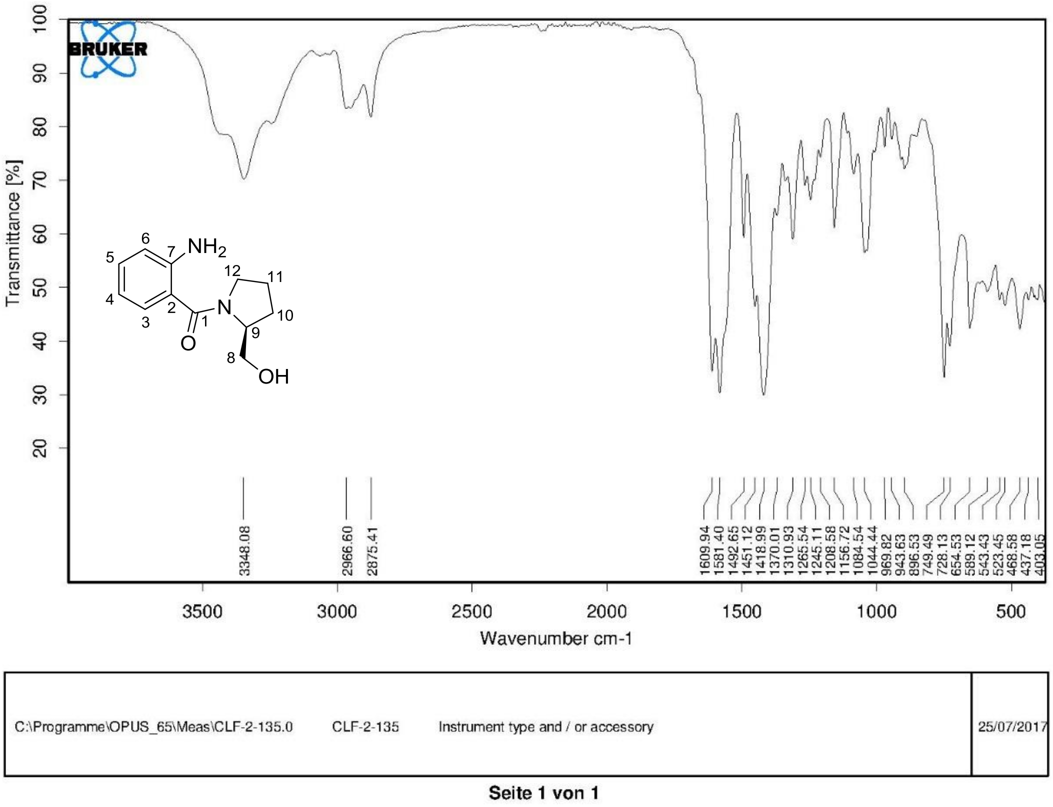

ATR-IR spectrum of (*S*)-(2-aminophenyl)(2-(hydroxymethyl)pyrrolidin-1-yl)methanone.

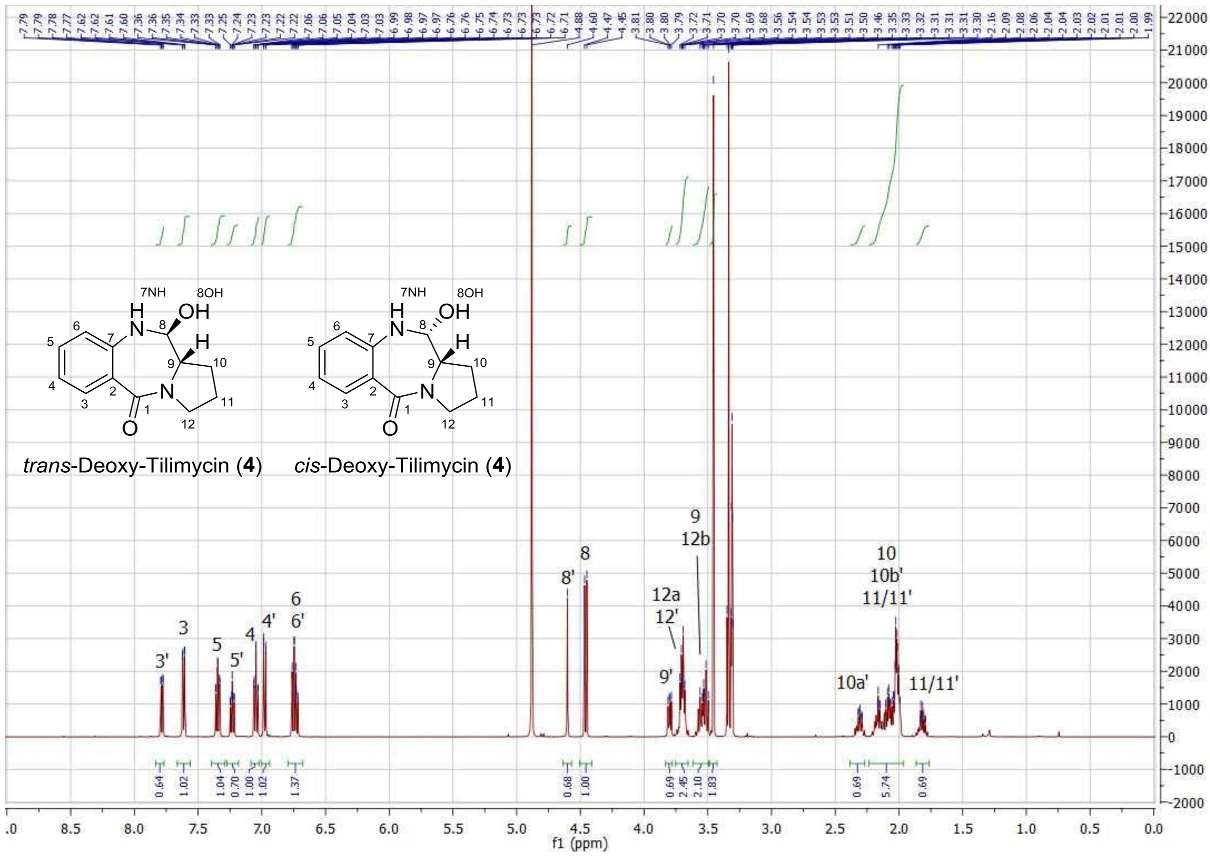

Proton NMR spectrum (500 MHz, MeOD-d$_4$) of synthetic *cis*/*trans*-deoxy-tilimycin (**4**).

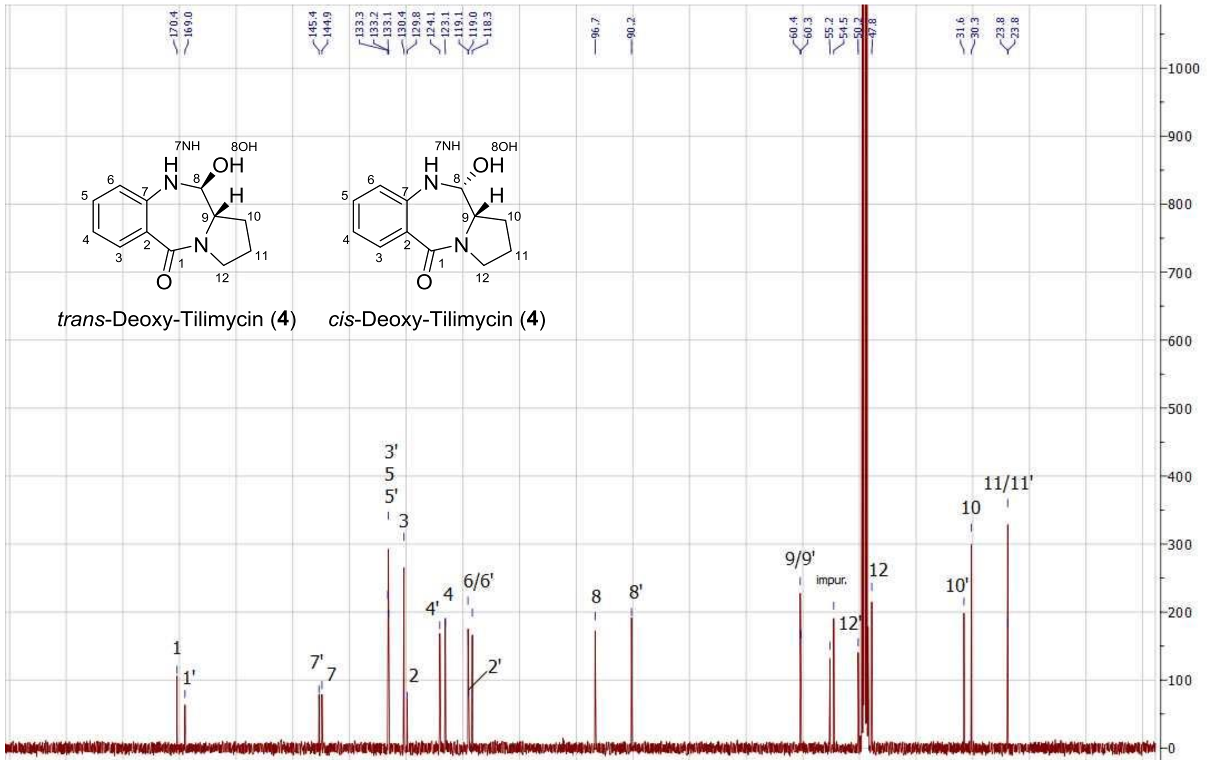

Carbon NMR spectrum (126 MHz, MeOD-d$_4$) of synthetic *cis*/*trans*-deoxy-tilimycin (**4**).

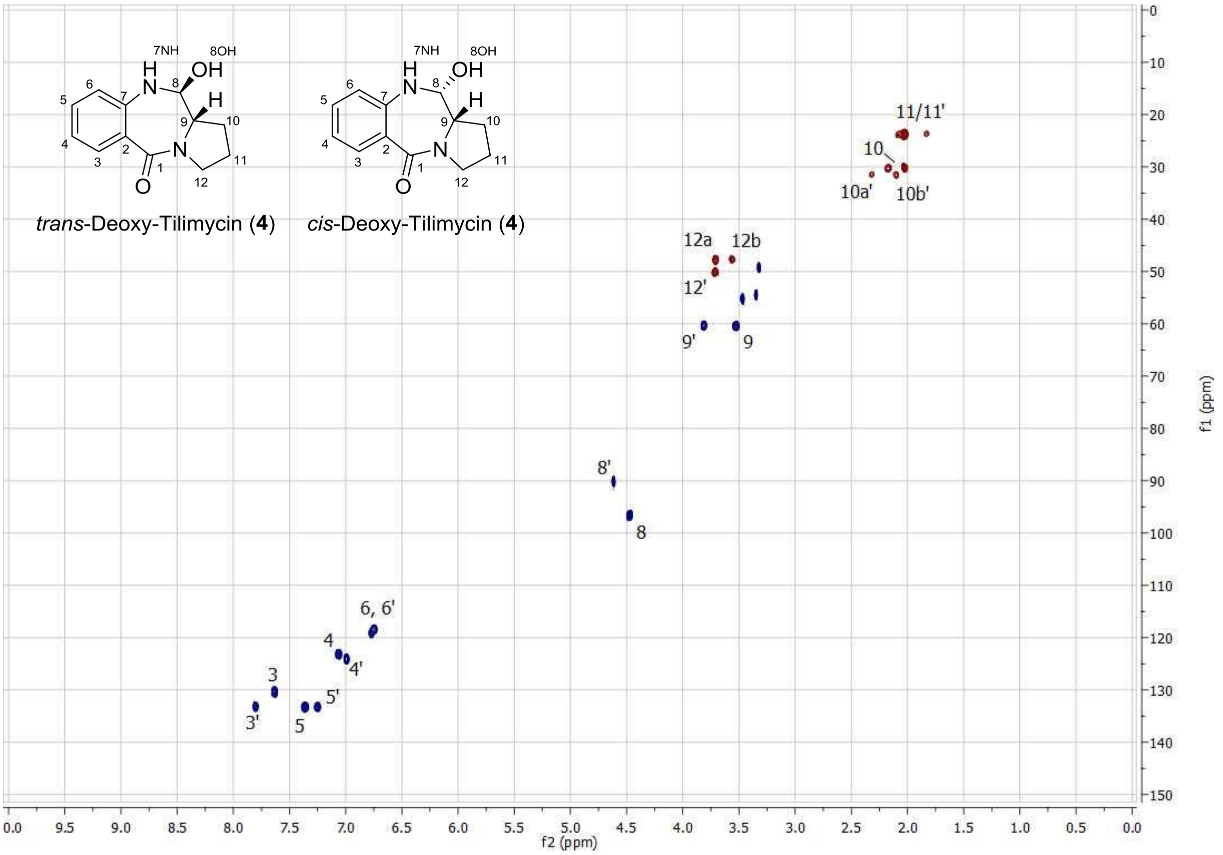

HSQC NMR spectrum (500 MHz, MeOD-d$_4$) of synthetic *cis*/*trans*-deoxy-tilimycin (**4**).

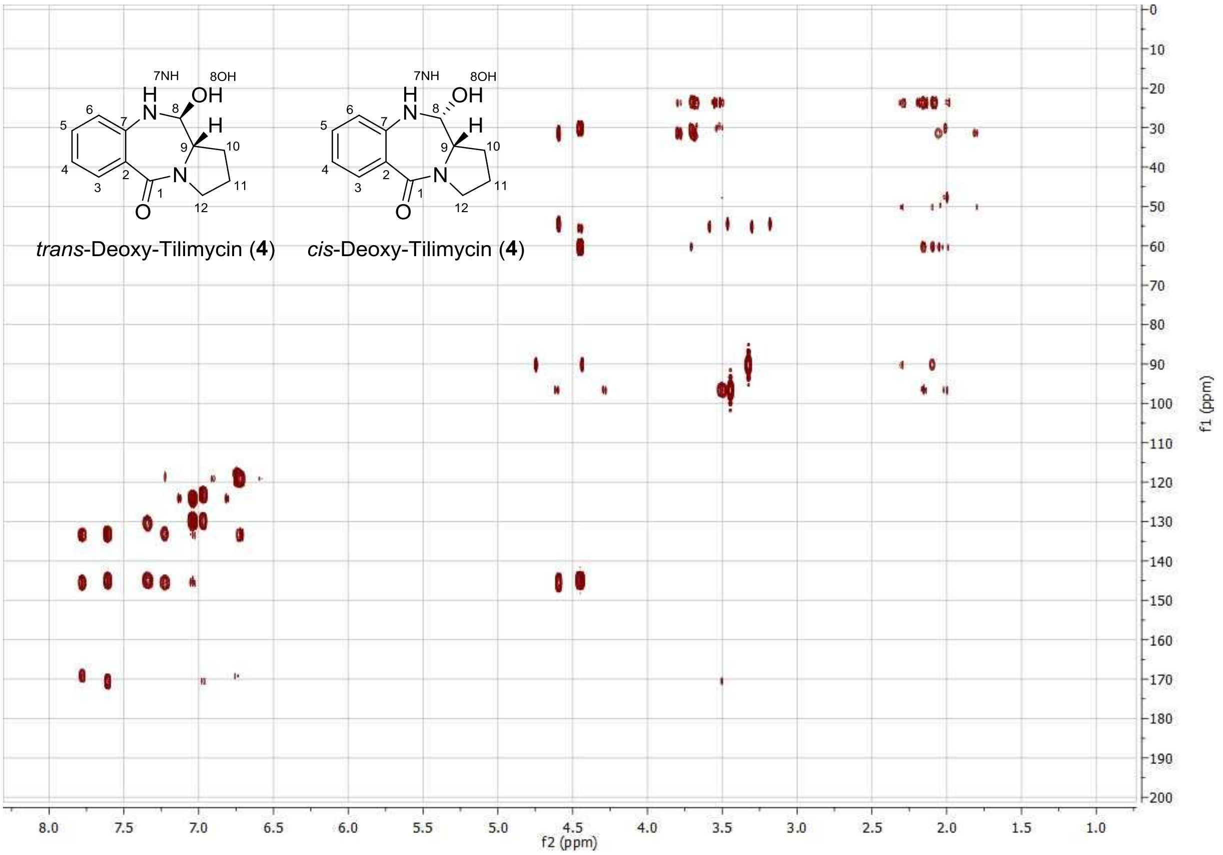

HMBC NMR spectrum (500 MHz, MeOD-d$_4$) of synthetic *cis*/*trans*-deoxy-tilimycin (**4**).

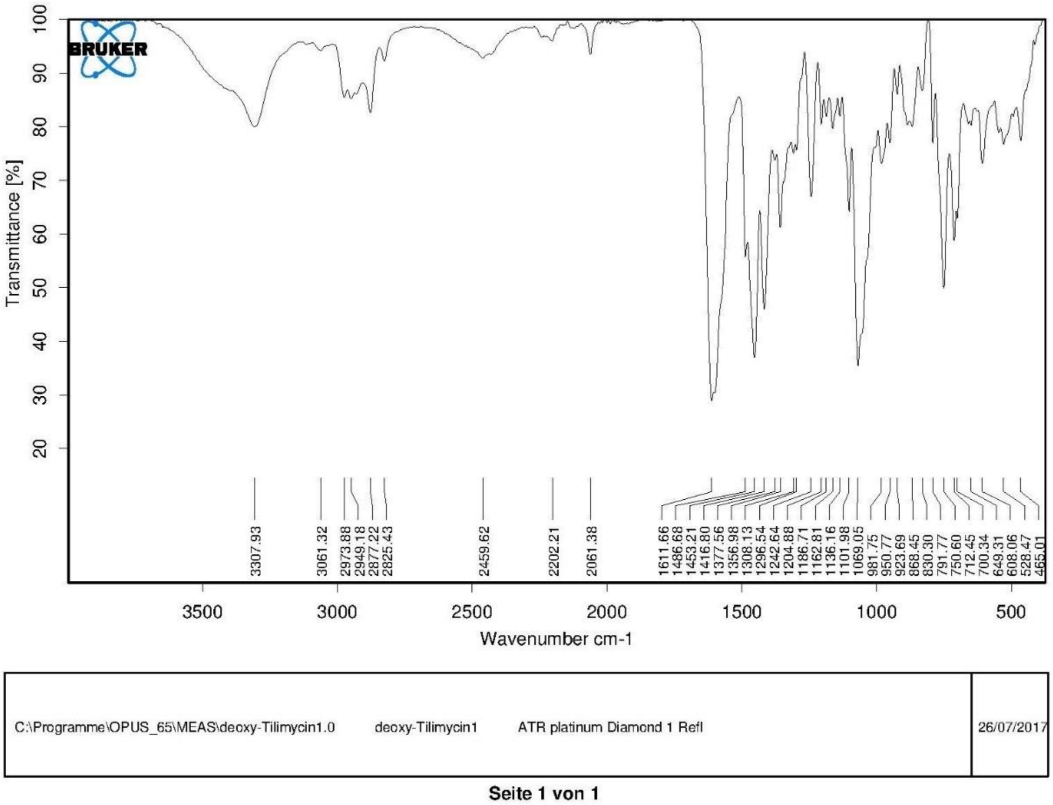

ATR-IR spectrum of synthetic deoxy-tilimycin (**4**).

References

[1] D. M. Woodcock, P. J. Crowther, J. Doherty, S. Jefferson, E. DeCruz, M. Noyer-Weidner, S. S. Smith, M. Z. Michael, M. W. Graham, *Nucleic Acids Res.* **1989**, *17*, 3469-3478.

[2] S. Lang, K. Gruber, S. Mihajlovic, R. Arnold, C. J. Gruber, S. Steinlechner, M. A. Jehl, T. Rattei, K. U. Frohlich, E. L. Zechner, *Mol. Microbiol.* **2010**, *78*, 1539-1555.

[3] C. Hogenauer, C. Langner, E. Beubler, I. T. Lippe, R. Schicho, G. Gorkiewicz, R. Krause, N. Gerstgrasser, G. J. Krejs, T. A. Hinterleitner, *N. Engl. J. Med.* **2006**, *355*, 2418-2426.

[4] G. Schneditz, J. Rentner, S. Roier, J. Pletz, K. A. Herzog, R. Bucker, H. Troeger, S. Schild, H. Weber, R. Breinbauer, G. Gorkiewicz, C. Hogenauer, E. L. Zechner, *Proc. Natl. Acad. Sci. U. S. A.* **2014**, *111*, 13181-13186.

[5] M. K. Chaveroche, J. M. Ghigo, C. d'Enfert, *Nucleic Acids Res.* **2000**, *28*, E97.

[6] A. C. Chang, S. N. Cohen, *J. Bacteriol.* **1978**, *134*, 1141-1156.

[7] F. H. Darras, B. Kling, J. Heilmann, M. Decker, *ACS Med. Chem. Lett.* **2012**, *3*, 914-919.

[8] T. Nagasaka, Y. Koseki, *J. Org. Chem.* **1998**, *63*, 6797-6801.

[9] A. Soret, C. Müller, R. Guillot, L. Blanco, S. Deloisy, *Tetrahedron* **2011**, *67*, 698-705.

[10] J. Oyamada, T. Kitamura, *Tetrahedron* **2009**, *65*, 3842-3847.

[11] R. N. Ram, N. Kumar, *Tetrahedron* **2008**, *64*, 10267-10271.

[12] T. Nakamura, T. Noguchi, H. Kobayashi, H. Miyachi, Y. Hashimoto, *Chem. Pharm. Bull* **2006**, *54*, 1709-1714.

[13] B. R. Lichman, M. C. Gershater, E. D. Lamming, T. Pesnot, A. Sula, N. H. Keep, H. C. Hailes, J. M. Ward, *FEBS J.* **2015**, *282*, 1137-1151.

[14] S. Mori, T. Ohno, H. Harada, T. Aoyama, T. Shioiri, *Tetrahedron* **1991**, *47*, 5051-5070.

[15] T. Matsumoto, N. Matsunaga, A. Kanai, T. Aoyama, T. Shioiri, E. Osawa, *Tetrahedron* **1994**, *50*, 9781-9788.

[16] T. Mosmann, *J. Immunol. Methods* **1983**, *65*, 55-63.
